# De-implementation of inappropriately tight control (of hypoglycemia) for health: protocol with an example of a research grant application

**DOI:** 10.1186/1748-5908-9-58

**Published:** 2014-05-19

**Authors:** David C Aron, Julie Lowery, Chin-lin Tseng, Paul Conlin, Leila Kahwati

**Affiliations:** 1 Interprofessional Implementation Research, Evaluation and Clinical Center, Louis Stokes Cleveland Department of Veterans Affairs Medical Center (EUL Room 5M677), 10701 East Boulevard, Cleveland, OH 44106, USA; 2 VA Health Services Research & Development Quality Enhancement Research Initiative-Diabetes, VA Ann Arbor Health Care System, 2215 Fuller Road, Ann Arbor, MI, USA; 3 Research Service, Department of Veterans Affairs, New Jersey Health Care System, 385 Tremont Ave., East Orange, NJ, USA; 4 Medical Service, VA Boston Healthcare System, Jamaica Plain Campus 150 South Huntington Avenue, Jamaica Plain, MA, USA; 5 RTI International, Research Triangle Park, NC, USA

## Abstract

**Background:**

Implementation of practice change is difficult and large scale implementation is particularly difficult. Among the challenges facing the healthcare system in general and healthcare organizations is the overuse of low value care. Improving medication safety also constitutes an attempt to reduce low value or potentially harmful care. Critical issues of overuse of low value practices and medication safety intersect in overtreatment of diabetes. Specifically, (over)intensive glycemic control increases hypoglycemia risk and morbidity without providing meaningful benefit. Our work indicates that among patients with diabetes who are at high risk for hypoglycemia, potential overtreatment is common. The Choosing Wisely Initiative to reduce low value care led by the American Board of Internal Medicine Foundation recommends not to treat most persons over 65 years of age with medications to reduce the A1c<7.5%. For most physicians this involves a change in practice. We will study the implementation of the Veterans Health Administration's Choosing Wisely Initiative (which includes hypoglycemic safety as a targeted condition) with three specific aims: (1) to assess the overall impact, both intended and unintended, of the Choosing Wisely Initiative to reduce overtreatment of diabetes in especially vulnerable populations; (2) to assess the impact of commitment to quality, teaching intensity, and safety culture on likelihood of overtreatment; and (3) to identify configurations of the implementation strategy, provider characteristics and organizational level factors that are associated with successful reduction of overtreatment rates by comparing high and low performers. Because focus on this initiative could have the unintended consequence of paying less attention to poor glycemic control (A1c>9%), we will also assess undertreatment.

**Methods/Design:**

We will take advantage of a natural experiment and use a Type III Hybrid Design that focuses on study of implementation while at the same time observing and gathering information on clinical interventions and outcomes. This mixed methods study will use longitudinal data and qualitative methods including Qualitative Comparative Analyses.

**Discussion:**

Our multi-paradigm approach to examining potential mechanisms to explain the variation in reduction of rates of overtreatment will contribute to a better understanding of implementation of national dissemination projects and multi-component interventions in complex systems.

## 

There are many guides to developing research proposals, and ingredients for implementation research grant proposals have been suggested by Proctor *et al.*[1]. However, among the challenges facing a new investigator in trying to get research funding is the relative paucity of model grant applications [2,3]. This is particularly true when the field being entered is a newly developing discipline; implementation science is such a discipline and it is still evolving [4]. Although the publication of protocols from such grant applications has become more common, the actual grant application and its iterations have not. Our goal is not only to provide an example of an implementation research grant application, but also to illustrate this process further by making available the different iterations and the critiques as well. In so doing, we take the process one step further by illustrating how the research team revised the application in response to the critiques. Each funding agency has its own application procedures. These procedures may differ in the details, but are similar. Some require ‘letters of intent’ or ‘concept papers’ while others do not. The revised application is in Additional file 1. We have included in the six other additional files labeled: Additional files 2, 3, 4, 5, 6 and 7.’ This process lasted from May 2013 to March 2014. Of note, in the middle of the process, the funding agency changed its requirements between initial and revised submissions in terms of the length of the narrative, reducing it from 25 to 15 pages.

Proctor *et al*. identified ten ingredients of a successful implementation research grant proposal [1]. These ingredients are listed in Table 1. All ten ingredients were included in our application to varying degrees. No claims are made that this is the optimal proposal that could be written on the subject, merely that it suffices, *i.e.*, it was good enough to have been funded successfully [5], and they are highlighted in Table 1. It is the hope of the authors that making this material available, with all its imperfections, will foster development of this crucially important discipline. It should also be stated that the process of submission and review resulted in what we believe is a much improved proposal.

**Table 1:** **Ten key ingredients for implementation research proposals (modified from Proctor EK et al.**[1]**)**

**Ten key ingredients**	**How ingredients are operationalized**
Evidence of a gap in quality	In addition to support from the literature, research team members were able to cite their own work about potential overtreatment of diabetes. Another gap is in knowledge about how to de-implement well-established practices.
Evidence-based treatment to be implemented	Discontinuing treatment that is of little benefit, but potentially harmful is valid on its face.
Conceptual model and theoretical justification	Both the original application and the revision modified an established conceptual model. However, in response to the reviews, the aspects of the model related to ‘unlearning’ were eliminated.
Stakeholder priorities, engagement in change	This project involved assessment of a natural experiment. The priorities were set by central administration. However, it occurred in the context of similar initiatives in the private sector.
Setting’s readiness to adopt new services/ treatments/programs	Preliminary data provided some support for the readiness of the settings, but variation is expected and is the focus of the proposal.
Implementation strategy/process	As a natural experiment, implementation strategy and process were outside the control of the research team.
Team experience with the setting, treatment, implementation process	The team members have had a long track record of working together in the general area of diabetes care delivery. They have special expertise in implementation research as well as operational implementation.
Feasibility of proposed research design and methods	Feasibility was a major factor in designing a multi-level (national and local facility) study based on different kinds of data.
Measurement and analysis section	This section was one of the largest in the application.
Policy/funding environment; leverage or support for sustaining change	It is clear that this topic of potential overtreatment of diabetes has gained considerable traction: In addition to the Choosing Wisely initiative itself, professional societies have adopted the concept of individualization of A1c targets and modified their practice guidelines accordingly.

## Competing interest

The authors declare that they have no competing interests. The views expressed are those of the author and do not represent the views of the Dept. of Veterans Affairs or any other agency.

## Authors’ contributions

DA drafted the introductory article. For the additional files constituting the grant application, DA (Principal Investigator) drafted the first version. JL (co-Investigator) drafted the section on qualitative methods. LK (co-Investigator) drafted the section on qualitative comparative analysis. C-LT (co-Investigator) drafted the statistical methods section. PC contributed to the writing. Multiple drafts of the grant application were reviewed and critically by all authors. The authors of the critiques were anonymous. All authors read and approved the final manuscript.

## Supplementary Material

Additional file 1 Specific aims. Click here for file

Additional file 2 Concept paper. Click here for file

Additional file 3 Critiques of the concept paper. Click here for file

Additional file 4 Initial submission. Click here for file

Additional file 5 Critiques of the initial submission. Click here for file

Additional file 6 Response to the critiques. Click here for file

Additional file 7 Critiques of the revision. Click here for file
