# Supplementary material for: De-implementation of inappropriately tight control (of hypoglycemia) for health: protocol with an example of a research grant application
Source: Implement Sci. 2014 May 19;9:58. doi: 10.1186/1748-5908-9-58 (PMC4046046; doi:10.1186/1748-5908-9-58)
Supplement: Additional file 1 — Specific aims. [file 1748-5908-9-58-S1.pdf]

2. Specific Aims. Implementation of practice change is difficult and large scale implementation is particularly difficult. Among the challenges facing the healthcare system in general and healthcare organizations such as VHA is the overuse of low value care. Reducing low value care has become a recent focus of VA's quality improvement efforts as evidenced by the VA's creation of a Choosing Wisely Taskforce to recommend how this national initiative can be implemented. Improving medication safety also constitutes an attempt to reduce low value or potentially harmful care. Critical issues of overuse of low value practices and medication safety intersect in overtreatment of diabetes (DM). Specifically, (over)intensive glycemic control increases hypoglycemia risk and morbidity without providing meaningful benefit. Our work indicates that among patients with diabetes who are at high risk for hypoglycemia – due to use of specific hypoglycemic agents, age, and/or significant comorbidities – up to 50% are potentially overtreated, as defined by an A1c <7%. National recognition for hypoglycemic safety is evidenced by the creation of a Health and Human Services Federal Interagency Workgroup (with representatives from VA and DoD) to address adverse drug events, including those from hypoglycemic agents. Moreover, the Choosing Wisely Initiative to reduce low value care led by the American Board of Internal Medicine Foundation includes an American Geriatric Society recommendation to "not treat most persons over 65 years of age with medications to reduce the A1c<7.5%." For most physicians this involves changes to their current clinical practice. VHA's soon to be announced response to the Choosing Wisely Initiative will include hypoglycemic safety as one of its targeted conditions (see letters of support from Drs. Schectman/Pogach). Our goal is to study the implementation of the Choosing Wisely Initiative as it plays out at the facility and practitioner levels. We will assess the concomitant implementation of processes of reducing clinically inappropriately tight glycemic control (de-implementation) as well as assess the effectiveness of a toolkit being promoted by this initiative. Because focus on this initiative could have the unintended consequence of paying less attention to poor glycemic control (A1c>9%), we will also assess undertreatment. In so doing, we will inform both VHA and the broader federal health community. We propose three specific aims and associated hypotheses (H) and research questions (RQ):

Aim 1. To assess the ***overall*** impact, both intended and unintended, of the Choosing Wisely Initiative.

H1.1. Patients treated in facilities that focus on hypoglycemia risk reduction will have a lower likelihood of overtreatment compared to those treated in other facilities.
H1.2. Patients treated in facilities that focus on hypoglycemia risk reduction will have the same likelihood of undertreatment compared to those treated in other facilities.

Aim 2. To assess the impact of commitment to quality, teaching intensity, and safety culture on patients' likelihood of overtreatment.

H 2.1a. Patients treated in facilities with higher levels of commitment to quality are less likely to be overtreated compared to those in facilities with lower levels of facility commitment to quality.

H2.1b. Patients treated in facilities with higher level of teaching intensity are less likely to be overtreated compared to those in facilities with lower level of teaching intensity.

H2.1c. Patients treated in facilities with higher level of safety culture are less likely to be overtreated compared to those in facilities with lower level of teaching intensity.

Aim 3. To identify configurations of the implementation strategy (Choosing Wisely toolkit), provider characteristics and organizational level factors that are associated with successful reduction of overtreatment rates by comparing high and low performers. Our sample size will be insufficient for formal hypothesis testing, but we will examine the following research questions using matrix and qualitative comparative analyses:

RQ3.1. Which configurations of the Choosing Wisely toolkit are associated with greater reduction in overtreatment rates?

RQ3.2. Which configurations of provider characteristics and organizational factors are associated with greater reduction in overtreatment rates?

We propose a Type III Hybrid Design that focuses on study of implementation while at the same time observing and gathering information on clinical interventions and outcomes.[1, 2] We take advantage of a VA national initiative (Choosing Wisely) with its associated toolkit) that will roll out ~April 1, 2014. This constitutes a natural experiment in which "events, interventions or policies which are not under the control of researchers, but which are amenable to research which uses the variation in exposure that they generate to analyse (sic) their impact."[3] This project will advance implementation science by using an innovative mixed methods multi-paradigm approach to examine potential mechanisms to explain the variation in reduction of rates of overtreatment and to contribute to a better understanding of implementation of national dissemination projects and multi-component interventions in complex systems.

2a. Research Plan.

2.1 Background. This time-sensitive project takes advantage of a national initiative to reduce adverse drug events with a specific focus on preventing hypoglycemia by reducing rates of inappropriately intense glycemic control (overtreatment) in vulnerable individuals. The VA's commitment to the Choosing Wisely Initiative (CWI) was established by the Deputy Under Secretary for Policy and Services, and the chartered taskforce is co-led and sponsored by VA Patient Care Services (both Specialty and Primary Care) with the support of Pharmacy Benefits Management (PBM) and other VA offices, including the Office of Informatics and Analytics. This initiative will initially involve a recommendation for two topics for VISNs to consider for adoption: reducing risk of adverse drug effects related to diabetes overtreatment and reducing MRI use in low back pain evaluation. Choice of focus may be at the VISN or facility level. Toolkits to facilitate action will be provided by VA. PBM has committed its field-based clinical pharmacy specialists with prescription privileges to supporting the hypoglycemia risk reduction initiative. The rationale for the initiative and the proposed project is as follows:

Hypoglycemia in diabetes is common, serious, and multifactorial in origin. In a study of over 14,000 patients in the Kaiser Permanente Northern California Health Plan, at least one significant hypoglycemic episode (losing consciousness or requiring outside assistance) in the prior 12 months was reported by 11% of patients with the highest risk for those on insulin; about 30% of those receiving insulin reported at least one serious event.[4] Of the participants who reported at least 1 significant hypoglycemic episode, 8% had evidence of a documented ER visit or hospitalization for hypoglycemia in the prior year, compared to 1.6% of the participants who reported not having significant hypoglycemia. Insulin is the second most common drug associated with ER visits for adverse drug effects. Hypoglycemia rates based on administrative data are underestimates because of under-coding, especially in the ambulatory setting. In one VA study 10% of patients with diabetes had a relevant ICD-9 code or other indication of hypoglycemia.[5] In addition to use of insulin or sulfonylureas, risk factors for hypoglycemia include intensive glycemic control,[6-8,9] chronic kidney disease (CKD), and cognitive impairment.[6] Both CKD and cognitive impairment are common in VA patients and many are (potentially) overtreated.[5] A recent study suggested an increasing healthcare burden of hypoglycemia in the United States from 1999-2010.[9] Rates of hospital admissions for hypoglycemia among Medicare beneficiaries increased by 22.3% (94 to 115 per 100,000 person-years) compared to a 39.5% decrease in the rate of hyperglycemia admission (114 to 69 per 100,000 person-years). Severe hypoglycemia was common among patients with type 2 diabetes across all levels of glycemic control. Risk tended to be higher in patients with either near-normal glycemia or very poor glycemic control.

Overtreatment (inappropriately intensive glycemic control) is common. We have developed a measure of "*potential* over-treatment" operationally defined as: A1c< specific threshold (<7%, <6.5%, or <6%) representing increasingly intensive glycemic control, treatment with insulin and/or sulfonylurea, and one of the following: (a) >75 years of age, (b) serum creatinine >2 mg/dl, or (c) cognitive impairment or dementia. Our study indicates that up to 50% of patients in VA meeting these criteria are potentially being overtreated. Moreover, there is evidence of considerable facility-level variation: Rates of overtreatment ranged from 6.1 to 23.0%, 20.4 to 45.9%, and 39.7 to 65.0% for A1c thresholds of <6%, <6.5%, and <7%, respectively. These measures are less "sensitive" than the American Geriatrics Association recommendation for the *Choosing Wisely Campaign,* which is, "Avoid using medications to achieve hemoglobin A1c <7.5% in most adults age 65 and older."[10] After excluding A1c<7% in the < 65 year old population and other conditions recommended by VA/DoD guidelines, ~65% receiving hypoglycemic agents would be considered at high risk for serious hypoglycemia. Tackling such a problem presents challenges at multiple levels of the healthcare system.

Many factors have contributed to the frequency and persistence of overtreatment. For more than 15 years the American Diabetes Association (ADA) guidelines have supported a target A1c of <7% for "most" adults aged 18-75. Similarly, performance measures reflect perspectives of different stakeholders, but form an important part of the organizational context of healthcare.[11] The National Committee for Quality Assurance's Healthcare Effectiveness Data and Information Set (NCQA-HEDIS®)'s A1c<7% measure for all patients aged 18-74 years was introduced in 2006 despite the unanimous opposition of the National Quality Improvement Alliance's Technical Advisory Panel.[11] New measures were introduced only after the premature termination of the ACCORD trial. Nevertheless, current measures now include <7% measure for persons less than 65 years of age without advanced diabetes complications, cardiovascular or ischemic disease, or dementia; and <8% measure was introduced for all other persons 18-75 years of age.[11, 12] We note that VHA did NOT endorse any of these measures, including the <8% measure, because of concern over hypoglycemia.

Unintended consequences of these dichotomous measures have included misplaced focus of clinical efforts, including inappropriate intensive glycemic control (overtreatment) and more frequent hypoglycemia.[11-14]

Changing practice has been difficult. Even in VA, which never adopted the HEDIS glycemic control measures, we find that the guidelines most commonly cited are those of the ADA and not VA/DoD,[15] and VA providers are influenced by the more general marketing efforts of professional societies and pharmaceutical companies. Moreover, "unlearning" an outmoded practice (de-implementation), especially one that has been well established and highly promoted, may be particularly difficult.[16, 17,18, 19] De-implementation has been observed to varying degrees for practices such as traditional therapy for duodenal ulcer, hormonal therapy for menopausal symptoms and prevention of heart disease, and use of antibiotics for uncomplicated sore throats.[15,20, 21,20,21] Sometimes a practice has been abandoned quickly; other times, old practices persist. In fact, Kale et al. showed that efforts to address underuse were more successful than those for misuse and overuse.[22] Whether or not de-intensification of glycemic control in appropriate patients will prove to be more difficult than changing other practices, it is clear that interventions to promote practice change will have to involve multiple levels of the healthcare system down to the provider and patient levels. Therefore, we have chosen to nest a model of provider behavior change (Theory of Healthcare Professionals' Behavior and Intention[23-25]) within the more global Greenhalgh et al. Model of Innovation Dissemination/Diffusion[26] (Fig.1). The Board of Internal Medicine Foundation's Choosing Wisely strategy of de-intensification of glycemic control in most patients >65 years for any medication other than metformin provides support for the focus upon patients at high risk for hypoglycemia as the innovation. Dissemination will be promoted by VA Knowledge Purveyors (disseminators) along with ongoing campaigns outside VA led by the Dept. of Health and Human Services for hypoglycemic safety, which was co-led by VA and specifically addresses the need for individualized targets. The dissemination package includes a toolkit, which targets different aspects of clinical decision making and other factors related to provider knowledge, self-efficacy, and systems operations. Many VA initiatives have been accompanied by performance measures; consideration is being given both in and outside VA to such a measure (see Pogach letter). The Type III Hybrid design recognizes not only that the focus of the project is the study of implementation, but also that initiatives may be undertaken without completing the portfolio of efficacy and effectiveness studies,[1, 2] reflecting real world practice.

Figure 1. *Integrated Conceptual Framework based on* **Greenhalgh et al. Model of Innovation dissemination/diffusion (rounded boxes)**; Theory of Healthcare Professionals' Behavior and Intention (square boxes) *is nested and impacts Adoption/Assimilation*

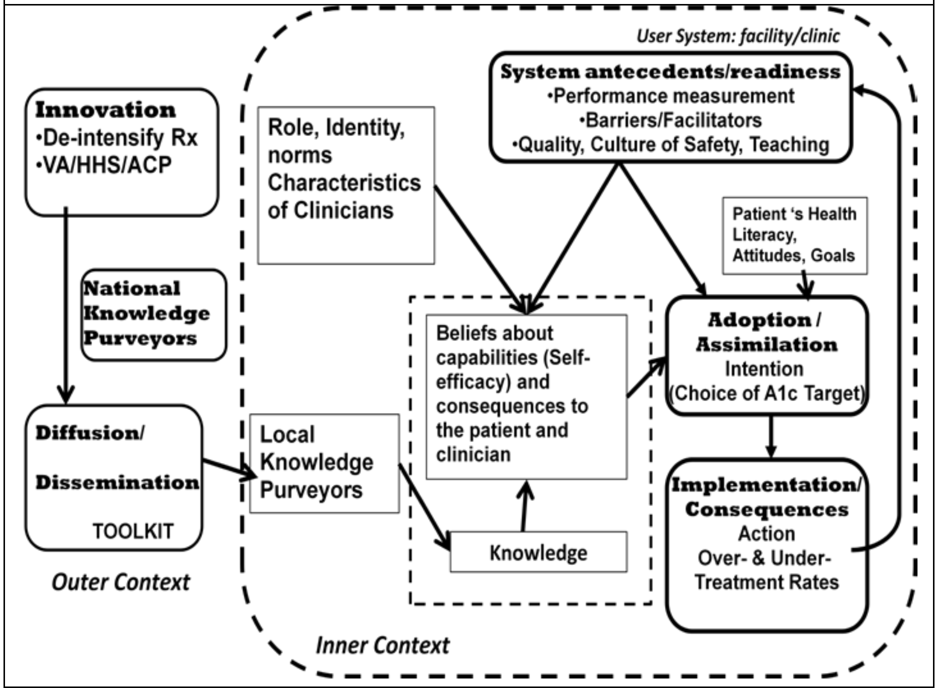

2.2. Significance. The critical issues of overuse of low value practices and medication safety intersect in overtreatment of diabetes (DM). Our project, which focuses on the intersection, has implications for policy (population health level), clinical practice (individual patient level), and research. Our project is also aligned with and supports efforts of VA Patient Care Services (both Specialty and Primary Care) and Pharmacy Benefits Management related to VA's Choosing Wisely Initiative. VHA provides care to about 5.6 million of the enrolled 8 million Veterans; and approximately 20-25% of patients have DM, half of whom are over 65 years of age (the target age for Choosing Wisely). Since 1997 it has issued clinical practice guidelines that have recommended individualized targets and warned against tight control in Veterans with significant comorbid conditions or decreased life expectancy. As a consequence of landmark clinical trials (ACCORD, ADVANCE, and VADT), increasing attention is now being paid to the risks and the need to individualize glycemic targets, and new guidelines have been issued by professional societies and advocacy groups.[26, 27] In contrast, for >15 years the VA/DoD DM clinical practice guidelines included targets stratified by life expectancy and diabetes complications, but their adoption within VA has been hampered, in part, due to strong marketing of other guidelines by professional societies and pharmaceutical manufacturers, e.g., the American Diabetes

Association (ADA), that promoted a single A1c<7% target.[27] In a recent review of NHANES data, about half of all adults with diabetes ≥20 years of age would have personalized targets ≥7%.[28] Moreover, recent publications on individualization of targets and "systematic" reviews of guidelines have tended to ignore those from VA/DoD.[27, 29-31] Our project will inform policy related to marketing VA/DoD guidelines and interventions to reduce hypoglycemia. The planned initiative is consistent with recent efforts to reduce the use of low value practices. A *Choosing Wisely Campaign* recommendation is, "Avoid using medications to achieve hemoglobin A1c <7.5% in most adults age 65 and older; moderate control is generally better."[10] Overtreatment is of low value not only because it lacks benefit, but also because it is potentially harmful.[32] This harm results from hypoglycemia, which has both individual and population health implications. Hypoglycemia is common, especially in patients treated with insulin and/or sulfonylurea drugs, and is underestimated and serious. Hypoglycemia is associated with morbidity, mortality, decreased health-related quality of life, and increased health services utilization (emergency department visits and hospitalization) and healthcare costs.[4,5,4,5,] Our recent publication indicates that up to ~65% of older Veterans with diabetes on hypoglycemic agents are at high risk for hypoglycemia or adverse outcomes because of advanced diabetes complications, serious medical and neurological conditions, decreased life expectancy, substance use, and cardiovascular disease. Though there is evidence that some practitioners take these factors into account,[33] there remains a large population at risk.

The Institute of Medicine has long proposed that prevention of adverse drug events (ADEs) should be a national patient safety goal. In addition, there is increasing recognition of hypoglycemia reflected in position papers from professional societies. Changing the paradigm for diabetes population health assessment (and management) by emphasizing the importance of potential overtreatment and harms will require a concerted effort. Underscoring the importance of hypoglycemic safety, the Office of the Assistant Secretary for Health and Human Services (Office of Disease Prevention and Health Promotion, ODPHP) recently included these agents in an interagency partnership to develop a National Action Plan for ADE Prevention, modeled after the National Action Plan to Prevent Healthcare-Associated Infections. This plan was published in the Federal Register on September 3rd for public comment; and will be published in Spring 2014 (see draft in Appendix; L. Pogach is co-chair of the workgroup on Hypoglycemic Safety). Our data evaluation plan informed this committee's formal recommendations, as summarized in a recent presentation to the HHS Health Informatics Technology Policy, Quality Measures Workgroup. We note that the presentation on the ambulatory recommendations for hypoglycemic safety included our HSRD-funded work.[32, 34, 35]

We expect that the aims will provide information about how interventions for decreasing hypoglycemia, particularly in the ambulatory setting, can be conducted at the organizational and provider levels. For example, this initiative will test our measure of "potential over-treatment" in real world settings. There are large knowledge gaps concerning effectiveness of national initiatives that promote "de-implementation." Although implementation of a new practice that is considered superior may, by necessity, require de-implementation of an old practice, it is not clear whether or how these processes differ. In particular, intensive glycemic control has been promoted as an essential best practice for many years. Changing the mental models of individual practitioners may be more involved than merely implementing something new, e.g., a new drug or surgical procedure. This will likely require different improvement strategies than those used for implementation. Finally, from a manager's perspective, the question is not "does some strategy have efficacy under "ideal" conditions?", but rather "what works when or for whom?" Addressing these gaps will be necessary in order to design more effective interventions that support campaigns such as Choosing Wisely.

We also note that the project objectives are mentioned specifically in the DM-QUERI strategic plan, "Goal 2: To work with operations partners to promote evidence-based approaches to improve treatment and reduce complications of diabetes (Diabetes Management/ Secondary and Tertiary Prevention)." DM QUERI relies "on our relationships with PCS to ensure that our projects and activities are aligned with key operational priorities, such as implementation of the diabetes guidelines, current concerns related to hypoglycemia" (p48). Ensuring the safety of insulin and other glycemic control regimens, and avoiding hypoglycemia, particularly in the elderly, is an important focus of the Diabetes QUERI because of the adverse consequences of severe or recurrent hypoglycemia (p10). There is considerable DM-QUERI involvement. The PI is a member of the Executive Committee and former Co-Clinical Coordinator. J. Lowery is Co-Implementation Coordinator and P. Conlin is Chair of the Executive Committee. In addition, L. Pogach and S. Kirsh, members of the Steering Committee, are co-Clinical Coordinators. DM-QUERI supports this research not only for its contribution to the accomplishment of DM-QUERI Goal 2, but also for its anticipated contribution to implementation science. Aim 2's results will inform the DM-QUERI's efforts to use the Consolidated Framework for Implementation

Research (CFIR) for conducting a synthesis of findings across QUERI implementation studies. Of note, Implementation Science Synthesis was one of the program topics at the 2013 Global Implementation Conference (August 19-21, Washington, DC), where the use of CFIR for implementation science synthesis was presented and was well-received. Thus, our project will address the needs of implementation researchers, clinicians, managers, and policy makers.

Our study is also consistent with the Department of Health and Human Services Strategic Framework for Optimum Health and Quality of Life for individuals with multiple chronic conditions. Our use of individual longitudinal data can inform the work of the HHS Health Information Technology Policy Committee's Quality Measures Workgroup to address longitudinal measurement/delta(change) measurement.

The need to reverse poorly supported but established practices is being increasingly recognized, as evidenced by the American Board of Medicine Choosing Wisely campaign and analogous efforts in the UK. Moreover, reversal of recommendations is common. For example, a recent study reviewed 1344 articles in the New England Journal of Medicine over a 10-year period that examined new or established medical practices; 363 articles looked at established practices. Of these 363 articles, 146 (40.2%) found the current medical practice to be inferior to a lesser or prior standard, whereas 138 (38.0%) reaffirmed the current practice.[36] In summary, our study is designed to address gaps in our understanding of the de-implementation process, particularly at organizational levels – healthcare system and practice site.

2.3. Impact. There is considerable potential impact on improving the quality, effectiveness, and efficiency of health care for hundreds of thousands of Veterans on hypoglycemic agents (prescribed by VA providers) who are at higher risk for serious hypoglycemia because they are receiving inappropriately intense glycemic control. For example, in 2009, of the 205,857 Veterans taking insulin and/or sulfonylureas who were >75 years age, had a creatinine >2.0 mg/dl or had cognitive impairment/dementia, > 20,000 had a last HbA1c value <6.0% and >100,000 had a last A1c<7%.[37] Including other serious co-morbid conditions could increase the number of Veterans at risk to more than 450,000 individuals, or nearly two-thirds of all Veterans on these agents. It is likely that incentives to reduce low value care will be a prominent part of health care in the future, both in VA and in the private sector; high value care has been promoted as the preferred strategy for healthcare organizations including accountable care organizations.[38, 39] As one of VA's initial efforts to reduce low value care, there is great potential to learn lessons that can be applied to other such initiatives, thereby promoting more rapid translation of research into practice.

As in all emerging disciplines, implementation science has a rich and varied research agenda. Among the agenda items are research on the important attributes of new knowledge and how these influence its uptake (or not). This would include the attributes of and applicability of what is regarded as evidence by different individuals and in different contexts. This may have particular relevance to initiatives such as Choosing Wisely's focus on low value care, because value determination is affected by an individual's particular perspective. Moreover, our use of the CFIR will add to our knowledge of measuring contextual factors. Assessing efficacy by means of determining average effect size, preferably in an RCT, is an integral part of implementation science. However, critical to the effective implementation of context dependent and socially complex interventions is determining what works for whom in what circumstances. This involves mixed methods and multi-paradigm approaches, e.g., traditional positivist and realist evaluation.[40] Among the crossover methods is qualitative comparative analysis (QCA), which while well accepted in some disciplines, e.g., political science, has had relatively little use in health care. Our project will add to the knowledge about QCA and its use within healthcare. Finally, the proposed study is value-added beyond that likely to occur without the project. This value-added includes timely evaluation of a VA initiative that can inform both sustainability and future initiatives. Of note, our work has already informed this initiative as well as the HHS joint task force. We first reported at the 2012 ADA Scientific Meeting upon the development of a glycemic overtreatment indicator and updated the analysis at the 2013 ADA Scientific Meeting to include regional variation in overtreatment rates. The National Hypoglycemic Action Plan referenced key conceptual elements of our ambulatory care measure for possible hypoglycemic overtreatment, as well as the key technical specifications. Additionally, the measure was endorsed by the Office of the National Coordinator for Health Information Technology, and was presented to that Office'sQuality Measures Workgroup in 6/13. Within VHA, the Deputy Under-Secretary for Policy and Services has chartered a VA Choosing Wisely Workgroup (co-chaired by Drs. G. Schectman, Chief Consultant for Primary Care, and L. Pogach, National Director for Medicine, Specialty Care Services). One of the first two topics prioritized for implementation will be glycemic overtreatment. This also has the potential to inform the American Board of Internal Medicine Foundation's Choosing Wisely measurement workgroup. Finally, since ~70% of our diabetes population is enrolled in Medicare because of age (≥65) or disability

(especially mental health disorders), and ~10% of VHA patients are enrolled in Medicaid, our results will be of interest to the Centers for Medicare and Medicaid Services.

2.4. Overview of Study Design and Timeline. We propose a 3-year mixed methods study (timeline shown in Figure 2) of the Choosing Wisely Initiative and its implementation, which constitutes a natural experiment. We have chosen a Type III Hybrid design in order to focus the assessment of the initiative on implementation while at the same time providing information about the effectiveness of different elements of the initiative's toolkit.[1, 2] We recognize that this initiative is being undertaken without prior studies of the toolkit's efficacy. However, we also recognize that toolkits have been a prominent part of VA's quality improvement efforts along with quality metrics.[41] Our mixed-methods analysis employs both prospective and retrospective elements, as well as multiple integration approaches to address both formative and summative assessments. It relies upon quantitative data for statistical inference validity of the overall impact of the initiative using both patient-level longitudinal data as well as facility-level aggregate data. Qualitative data will not only provide richness to the assessment, it will also contribute to interpretative rigor.[42, 43] Modes of mixing include triangulation (assessment of convergence) and sequential use (quantitative methods to identify low and high performers followed by qualitative assessment).

Figure 2

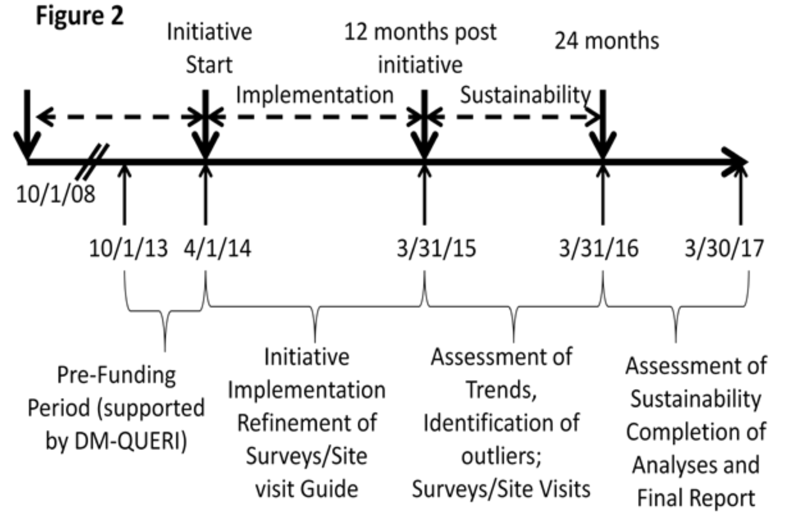

The Choosing Wisely Initiative, which will roll out April 1, 2014, will allow facilities to choose one of two overuse/low value care issues to address: reducing risk of adverse drug effects related to diabetes overtreatment and reducing MRI use in low back pain evaluation. Thus, for Aim 1, we will compare outcomes across four different groups of facilities: those who choose (1) overtreatment for diabetes; (2)MRI overuse for low back pain; (3) both; or (4) neither. Randomization is impractical, and implementation will vary among facilities both in terms of what is implemented and when it is implemented. Because intervention exposure will not be random, we will use special care in design, reporting and interpretation of evidence in accordance with MRC Guidance and draw causal inferences cautiously.[3] As such, this observational study will assess effectiveness rather than establish causality. For the purposes of the study, we consider the first year of the initiative to be the implementation phase. This will be followed by a 12 month sustainability phase.

For Aim 1 we will use a hierarchical ordinal logistic regression approach using patient-level longitudinal data (4/1/14 to 3/31/2015) to model the probability of overtreatment (and undertreatment) in which Choosing Wisely Initiative participation status (focus on overtreatment of diabetes, overuse of MRI for low back pain, both, or neither) is the key predictor variable of interest. Using patient-level data will provide power to identify even modest differences between groups. Aim 2 will utilize similar methods to assess associations with the facility characteristics of commitment to quality, teaching intensity, and safety culture. Aim 3 will utilize facility-level (cross-sectional) aggregate data (4/1/13 to 3/31/2016). High and low performers will be identified based on changes in overtreatment rates pre- and post-initiative (rather than on absolute values). In order to minimize issues related to regression to the mean as well as floor and ceiling effects, we will focus on facilities that were mid-performing at baseline (2nd and 3rd quartiles). We will focus on those facilities which had high reductions in overtreatment rates at 12 months (high performers) and those who had low (or negative) reductions in overtreatment rates, i.e., got worse, (low performers). Surveys and phone interviews will be conducted to assess what practices were (or were not) implemented to reduce overtreatment, as well as what barriers and facilitators existed to following the initiative. (DM-QUERI has committed funds to support pre-funding work, including instrument refinement and testing - see support letter). Field testing will continue in year 1.The purpose of the surveys of high and low performing sites is to provide the data for the qualitative comparative analysis (QCA) to assess which factors contribute to the difference in performance. Data from semi-structured interviews and site visits will enhance the interpretation. At year 2, the findings of the QCA will be assessed in depth via site visits to the high performing sites, with artifact collection, procedural observation, and in person semi-structured interviews conducted with clinicians (including CPSs, PCPs, and clinic managers), to enhance understanding of contextual factors. Our overall approach is based on the methods developed and used by the

HSR&D QUERI/Office of Specialty Care Evaluation Center (D. Aron and M. Ho, co-directors; J. Lowery – leader of Qualitative Analyses team). Similarly, QCA has been used to assess the national VA weight reduction program (MOVE).

2.4.1. Innovation and Toolkit. The innovation is the de-intensification of glycemic control to prevent hypoglycemia in vulnerable patients. The toolkit provides materials and recommendations to facilitate implementation. Our conceptual model, which is based on a diffusion/dissemination of innovation model, incorporates a focus on the provider, who along with his/her patient makes decisions about de-intensification of therapy. This focus is based on the Theory of Healthcare Professionals' Behavior and Intention, a modification of the Theory of Planned Behavior (Figure 1).[23, 24, 24, 25] Ever since the Diabetes Control and Complications Trial in 1993, the close relationship between glycemic control and DM complications (consequences to patients) has been stressed in the academic literature (knowledge source) with continuing medical education support from professional societies, advocacy groups, and by pharmaceutical manufacturers (knowledge sources of varying credibility and social influence). Practice guidelines (knowledge source) reflect the interests of these and other stakeholders. General recommendations of target A1c levels have been made by specialty societies and governmental agencies both in the United States and elsewhere.[29, 43] It is in this context that Choosing Wisely is being conducted and for which the toolkit is being developed. The toolkit is evolving and includes a variety of materials and more general recommendations, some of which were developed from the bottom up and others from the top down, reflecting the need for multifaceted approaches and allowance for local adaptation.[13,44,51-55] (See Toolkit materials in Appendix.) Although the individual elements have support in the literature,[17, 44, 45,50,51,52] we note that they have not been tested as a package, hence our choice of a Hybrid III study design. Nevertheless, this is the toolkit that will be used in the initiative, and our conceptual model will inform the facility- and provider-level assessments, as the domains are included in the surveys and interviews of high and low performers. We also expect that important disseminators will include many of the ~1,500 Clinical Pharmacists/ Clinical Pharmacy Specialist (CP/CPS) in 131 medical centers authorized to prescribe in Primary Care and/or Diabetes clinics (PBM – personal communication).[46]

2.4.2. Study Sites. All VA facilities with >100 Veterans with diabetes will be included. These sites will be classified by nature of participation in Choosing Wisely (focus on hypoglycemic safety, focus on low back pain, focus on both, or focus on neither). Previous experience (courtesy of National Program Director for Medicine) suggests that a minimum of 4-6 VISNs will focus on hypoglycemia safety alone, each with an average of 5 facilities. In addition, individual facilities in other VISNs may implement this intervention. (Note: VISN status will be included in our models.)

2.4.3. Study Populations. The population for Aims 1 and 2 will be VHA clinic Veteran users with diabetes receiving insulin and/or sulfonylurea treatment in the time period 4/1/14 to 3/31/2015 (the initiative start date being 4/1/2014). A Veteran is determined to have diabetes if one has VHA service use in the year and has two or more visits with diabetes ICD-9-CM codes (250.XX) from inpatient or outpatient (face-to-face) care over a two year period or has prescriptions for diabetes medication in the year. Aims 1 and 2 will utilize longitudinal data from Veterans with diabetes who have at least 1 A1c value. For our primary outcome measure of overtreatment (see below), we will include patients having at least one of the following additional criteria: age >75 years, last serum creatinine $\geq$2.0 mg/dl, or an ICD-9-CM diagnosis of cognitive impairment or dementia in ambulatory care. From a population health perspective, individuals 75 years of age and older have limited life expectancy and an increased co-morbid illness burden, and thus have decreased life-time benefit and increased risk from intensive glycemic control.[13-15] Impaired renal function affects metabolism of oral hypoglycemic agents and insulin.[21] In the ACCORD trial, subjects with serum creatinine levels between 1.0 and 1.3 mg/dl had greater risk of serious hypoglycemia than those with normal creatinine levels (<1.0 mg/dl); subjects with creatinine >1.3 mg/dl had even greater risk.[22] To be conservative, we have restricted our measure to even worse renal function. As a sensitivity analysis, we will evaluate patients with age greater than 70 years or a serum creatinine > 1.7 mg/dl. Cognitive impairment may adversely affect patients' ability to self-manage their diabetes and is associated with increased risk of serious hypoglycemia in both the control and intensive treatment arms of the ACCORD trial.[23]

2.4.4. Power Analysis. Power calculation in hierarchical/multi-level models is not straightforward and there are no established norms. The power calculation for such models needs to consider sample sizes in all levels involved. For a two-level model, a rule of 30 groups and 30 subjects per group is usually recommended to ensure sufficient power for fixed effects.[47] Using Optimal Design® software,[48-50] we conducted power analyses for detecting

differences in probability of overtreatment (e.g., A1c <6.5%) rates for facility-level predictors in a two-level model. We note that in 2009, there were ~205,000 with insulin/sulfonylurea who were ≥ 75 years old, or with either diagnosis of dementia or cognitive impairment, or having serum creatinine >2.0 mg/dl from at least 130 VA medical centers across the nation.[37] We also estimated that the number of such high risk patients in each facility range from 300 to 7,000. Based on 2009 data on the high risk patients, 28.6% (facility variation: 24.7%-32.7%) had their last A1c values < 6.5%. Since 2009, the number of patients has increased. We assumed a "control/reference "group probability of overtreatment of 0.290 based on our data from 2009. We assumed a Type I error rate of 0.05 for two-sided tests. We conducted power analysis based on 130 facilities and 300 (or 150) patients per facility. Figure 3 shows the relationship between power achieved and the overtreatment probability for the treatment group (P(TX)). The graph shows that when P(TX) moves away from 0.29 (namely, the group contrast becomes larger) and n is bigger, power increases. For example, we will have more than 0.8 power if P(TX)<=0.273 (detectable minimum group difference(d)=0.017) for n=300 and P(TX)<=0.268 for n=150 (d=0.022).. Our power calculations are based on 130 facilities with either 300 or 150 patients each and thus are extremely conservative and indicate that there will be sufficient power for subgroup analyses. The power calculation also has to consider the dependence among observations nested within the same cluster. However, we note that intraclass correlation for binary outcomes is less straightforward than for continuous outcomes.[51] In fact, Spybrook and Raudenbush who developed the Optimal Design software stated: "In the case of a binary outcome, we do not typically standardize the model because the level-1 variance is heteroskedastic, which makes the meaning of the intraclass correlation, ρ, uninformative."[49] Our conservative approach will accommodate even large intraclass correlations. Aim 3's selection of high and low performers is based on changes in facility-level overtreatment rates. We cannot be certain which facilities will choose to focus on hypoglycemia risk reduction as opposed to another Choosing Wisely topic area or none at all. Previous experience (courtesy of National Program Director for Medicine) suggests that a minimum of 4-6 VISNs will choose the hypoglycemia risk reduction focus, each with an average of 5 facilities. In addition, sites in other VISNs may implement this intervention. We obtained data from 2009 and 2010 to assess year to year variation. This allowed us to estimate power based on a paired t test for a simple pre-test post-test design. One hundred random samples of 20 facilities were chosen. The mean±1SD change in overtreatment rate for the A1c<6% threshold, a level that puts patients at the highest risk was an absolute percent reduction of -0.9±2.4, e.g., an overtreatment rate falling from 10% to 9.1%.Based on paired t test, our study will require as few as 15 facilities to detect an absolute overtreatment rate reduction of 2.9, e.g., 10% to 7.1%, with 85% power, even assuming as little as 50% correlation between the SDs of each of the two measurements. This compares favorably with preliminary work from VISN 12 which showed that rates of overtreatment fell by 4.6 to 5.9 at the three A1c thresholds.

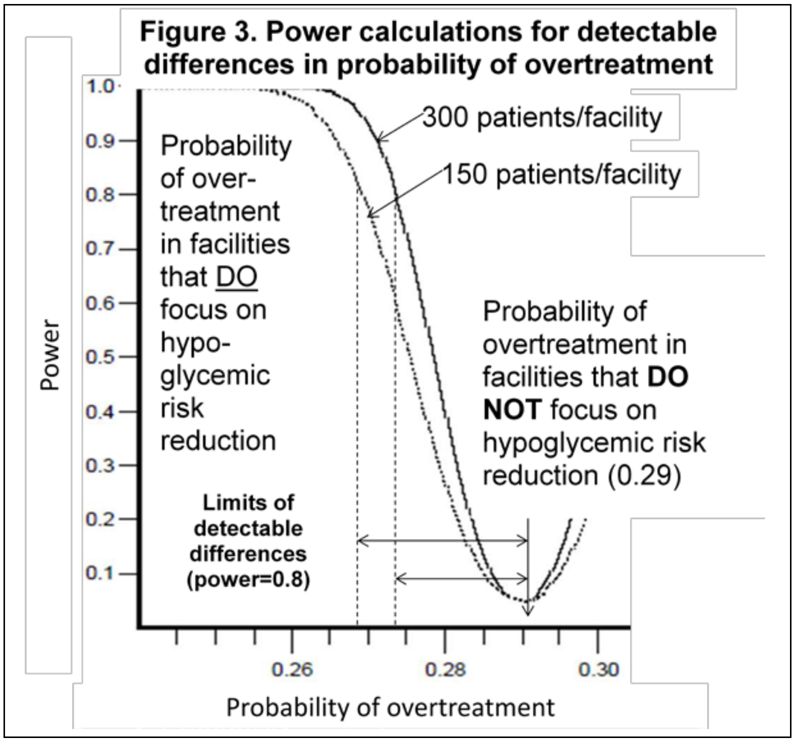

Figure 3. Power calculations for detectable differences in probability of overtreatment

2.4.5. Dependent and Independent Variables (Quantitative)

2.4.5.1. Dependent Variables (Outcome Measures for Summative Evaluation)

Overtreatment and undertreatment: Using the last A1c value in a time period we will define overtreatment if it is less than the thresholds of 6.5% (we will evaluate 6.0% and 7.0% for sensitivity analysis). If the same A1c value is greater than 9.0%, it is considered undertreatment. Those in the range of 6.5% to 9.0% are considered to be in moderate glycemic control and will be used as the reference group in statistical analyses.

2.4.5.2. Independent Variables (Primary Explanatory Variable, Process Measures, and Covariates)

Choosing Wisely Participation Status. We will use several methods to identify and confirm participation status at the 3-4 month time point after the initiative starts. If the information is reported to VACO,

we will obtain these data from the leadership of the VA's initiative (see Pogach support letter). We will contact key individuals in each VISN (Chief Medical Officer) and all facilities (Chief of Staff, Chief of Ambulatory Care, and others if necessary) by email with phone call follow-up if necessary. We will also survey by email the clinical pharmacy specialists at each facility. This approach will be used solely to determine participant status and not to obtain details regarding specific actions within a facility.

Commitment to Quality at Baseline. We will use overall quality of care as a surrogate and use the quality quintile (Gold Star rating) from first qtr FY2014 provided by Strategic Analytics for Improvement and Learning (SAIL) of the Office of Informatics and Analytics. These ratings are calculated quarterly and are a composite of a broad series of measures of all aspects of quality.

Teaching Intensity. Teaching status has been associated with quality of care.[52-54] Since the majority of VA hospitals are affiliated with medical schools, we will use a measure of teaching intensity. Teaching intensity = ∑ (# family practice trainee slots)+ (# internal medicine trainee slots)+ (# internal medicine subspecialty slots). These are the trainees who would be involved in primary care and specialty consultation related to diabetes. In 2013 this value had a mean±1SD of 39±33 with a range of 0-125. Of note, each trainee slot represents ~4 trainees rotating part time at the VA.

Safety Culture. We will use results from the National Center for Patient Safety Culture Survey, which is conducted every 3-5 years, the latest occurring in 2011. The survey which we plan to use in the analyses will occur after the initiative is underway. We will calculate a score based on results of the domains of "Overall Perceptions of Safety," "Perceptions of Patient Safety at your Facility," and "Senior Management Awareness/Actions in Promoting Safety." (See questions in the Appendix.)Scores will be divided into quintiles.

Comorbidities. Using ICD 9CM codes we will create indicators of advanced complications of diabetes, limited life expectancy, cardiovascular or ischemic vascular disease, and major medical and mental health conditions that would impact benefit or risk of tight control based upon previously published taxonomies.[20,24]

Demographic Variables. We have developed algorithms to process multiple entries of patient data over time and from a variety of sources to optimize the demographic information available for research. Sources include many years of VA inpatient and outpatient data with multiple fields and Medicare data. Consequently, we have nearly complete and largely confirmed assignments for most variables, including age and sex. Because of limitations in data concerning race/ethnicity, we will not assess this variable.

2.4.6. Identification of High and Low Performers. High and low performers will be identified based on changes in facility overtreatment rates pre- and post-initiative (rather than on absolute values). Those with the greatest (high performers) and least reduction (low performers) in overtreatment rates will be selected. To minimize issues related to regression to the mean, we will focus on facilities that were mid-performing at baseline (2nd and 3rd quartiles). See Fig. 4. Among the middle-performing facilities at baseline, we will select those facilities with the greatest reduction in overtreatment rates (high performers) and those with the least reduction (low performers) We will also select from among the highest performers at baseline who improved at all as well as some low performers at baseline who stayed low. It is impossible to know how many facilities will fall into these categories; but we are anticipating approximately 5-10 in each group (total n~30). Aim 3 does not include hypothesis testing, but is instead looking for identifying patterns of provider and facility characteristics associated with performance status (high vs. low). Our methods of analyses (matrix and qualitative comparative analysis) allow for a small sample size. Performance status will be determined independently of Choosing Wisely participation status; selected sites will likely include those facilities choosing the hypoglycemia initiative alone or both hypoglycemia and low back pain initiatives. If entire VISNs participate in the initiative, we will seek positive deviants, i.e., facilities which are statistically higher performers than other facilities in the same VISN. Positive deviants may provide special insights into successful implementation

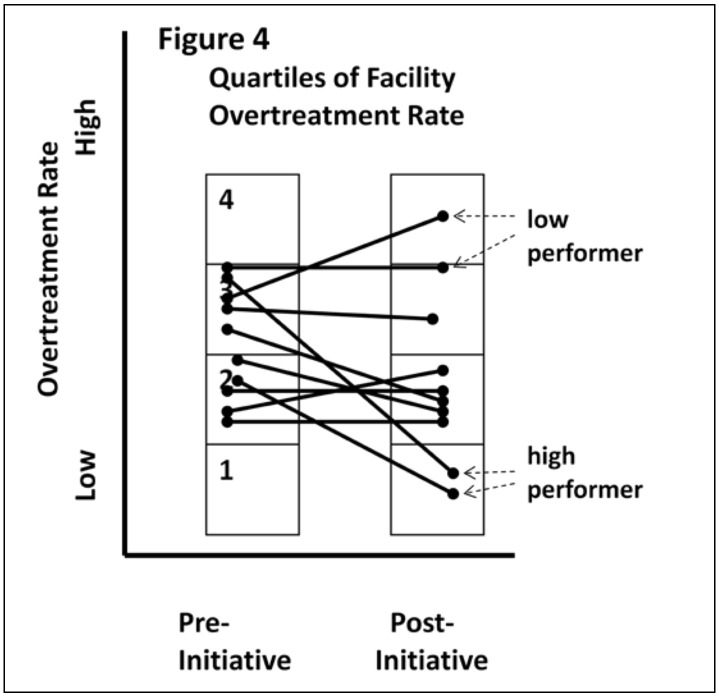

strategies. [55, 56] We will identify them as we have done previously using a statistical approach recommended by the National Committee of Quality Assurance.[22] In order to characterize these sites further as to their performance over time, we will also examine facility rates at baseline and at 2 years following the start of the intervention. Rates at 2 years will provide information about sustainability of improvement. We note that this study sample is a dynamic cohort since new members are added as they meet the study criteria.

2.4.7. Quantitative Data Sources and Collection. We will obtain VHA data from Corporate Data Warehouse (CDW), a national repository of data, and other VA files. The goal at VA has been to transition most VA data to reside on CDW servers. We will include files containing patient characteristics, outpatient and inpatient encounters, diagnosis and procedure codes, pharmacy/prescription information, and laboratory (A1c and serum creatinine) values from 2012 to 2016. We will use CMS data (when available), which includes Medicare (for outpatient and inpatient encounters, diagnosis and procedure codes, eligibility status) and Medicaid (for additional pharmacy/prescription information), as we have done previously.[35, 55] Data for commitment to quality, safety culture, and teaching intensity are described above (2.4.5.2). Specific data files are described below:

<u>Patient Medical Encounter Data</u> include file records for all VA inpatient and long-term stays and all outpatient visits, with information on patient characteristics, eligibility, type of care, and multiple codes for diagnoses (ICD-9-CM codes) and procedures (ICD-9-E or CPT4 codes). They are VHA Medical ASA (MEDSAS) datasets.

<u>VA Laboratory Data</u> are recorded at each site for all tests performed and subsets are extracted to national databases. We recognize that not all patients in our study will have results available from laboratory tests. In our prior work, we have found that, based on CPT codes, most VHA patients with diabetes receive the majority of their outpatient laboratory tests in VA clinics, and have regular measures of A1C and creatinine.

<u>Pharmacy Records</u> will be obtained from the Pharmacy Benefits Management Strategic Health Group (PBM). This national VA prescription database includes information on specific medications, including dates, duration, dose, and physician instructions. The PBM uses a verification process to assure data accuracy and completeness. Use of non-VA pharmacy services by VA patients is not included in PBM. To address this potential limitation, we will include data from Medicare Part D and Medicaid prescription records (if available). We note that such information may not be available for the entire study period due to time lags.

2.4.8. Quantitative Data Statistical Analyses. We will begin all analyses with inspection of frequency distributions and bivariate analyses of means (t-tests and ANOVAs) and proportions (Chi-square tests) among subgroups defined by our independent variables, and will use graphical methods for exploratory data analysis. We will enter variables into statistical models according to our conceptual framework and hypotheses. Cells in the cross tables with 0 or small numbers will be collapsed with other cells. In handling variables with missing data, the primary analyses will be restricted to those with values (complete case analysis); alternatively, patients with unknown values for a variable will be grouped into a subcategory to be included in the statistical models. Our analyses need to account for the possibility that observations may not be completely independent due to individuals nested within a VA facility, and VA facilities nested within a VISN. We will use hierarchical modeling (also termed multi-level models/mixed effects models/random-effects models) for the analysis of correlated data to address the issue of possible underestimation of variance for regression coefficient estimates under conventional regression models. In the statistical models, facilities and VISNs will be considered as random effects. Other than the primary exposure variable specified in each hypothesis, independent variables will also be included in the statistical models as control variables.

<u>Aim 1</u>.<u>To assess the *overall* impact, both intended and unintended, of the Choosing Wisely Initiative</u>. All analyses will be conducted for various subgroups of high risk patients. Due to the nature of the outcome variable in the quantitative aims (Aims 1 and 2), the primary statistical technique for these two aims will be logistic regression. The "logit" (i.e., log of odds) of the presence of an event is regressed on a list of independent variables. We will use odds ratios (=exponentiation of the estimated regression coefficients) and their confidence intervals for the hypothesis testing. An odds ratio larger (smaller) than 1 with a 95% confidence interval not containing 1 indicates a higher (lower) likelihood of the occurrence of an event for the group in comparison relative to the reference group in an independent variable. In Aim 1 we will use multinomial logistic regressions to model the outcome variable, a polytomous variable. In the models the polytomous variable is treated as a nominal variable and a value in the variable is treated as the reference group; separate logistic regression models for the probabilities of non-reference groups against the reference group are constructed and estimated simultaneously. This approach is more efficient than separately conducting two logistic regression models (i.e., the overtreated versus those in moderate control and the

undertreated versus those in moderate control). The primary exposure variable in both H1.1 and H1.2 is the group indicator variable. Along with the previously listed independent variables, a patient's glycemic control status in the pre-initiation period will also be included in the models as a control variable. We will assess both the implementation and sustainability phases of the initiative.

Aim 2. To assess the impact of facility commitment to quality, teaching intensity, and safety culture on patients' likelihood of overtreatment. We will use a similar modeling strategy to Aim1 for this aim but employ binary logistic regression models, because the hypotheses focus on overtreatment (a binary variable: overtreatment versus moderate control). We will construct separate models for each of H2.1a, H2.1b, and H2.1c. W will also evaluate the associations among the three primary exposures variables in the hypotheses, and construct a statistical model including all three variables and compare with the separate models.

2.4.9. Qualitative Analyses

Aim 3. To identify configurations of the implementation strategy (Choosing Wisely toolkit), provider characteristics, and organizational level factors that are associated with successful reduction of overtreatment rates by comparing high and low performers. Data collection and analysis for these research questions will use a mixed methods approach, as described below.

***Provider/Clinician/Clinical Manager Surveys*.** Provider surveys (see Appendix) will be administered approximately 1 year following implementation of the initiative to clinical leaders and PCPs and other clinicians at the low and high-performing sites. Survey domains are derived from our conceptual framework. [23-26] The surveys will obtain data on provider and organizational factors potentially affecting overtreatment rates. Provider characteristics include clinician type (MD, DO, NP, PA, CPS), years in practice, and gender. The survey is based on the Diabetes Attitudes Survey and the Veterans Assessment and Improvement Laboratory (VAIL) VISN 22 Survey of Primary Care Team Experiences.[56] We will also assess clinicians' knowledge and attitudes towards glycemic targets. We will assess efficacy using the Provider Abilities subscale from the Midwest Clinicians' Network Barriers to Diabetes Care Survey questionnaire, adding items modeled after Glazier et al.and questions about factors such as competing priorities.[57,58, 59] We will use the Consolidated Framework for Implementation Research (CFIR) framework to define potentially influential organizational factors .[60] The survey will consist of questions assessing the potential importance of each CFIR construct in implementing the Choosing Wisely Initiative. This survey has been used in the evaluation of four initiatives (E-Consults, SCAN-ECHO, Specialty Care Neighborhood, and Mini-Residencies) so far in the Specialty Care Evaluation project. We will survey up to 30 individuals in each of the ~30 identified low and high performing sites (~900 surveys). We will conduct the survey online (Inquisite® software) with follow up emails, letters, and phone calls as needed. Respondent burden will not exceed 25 minutes. Respondents will be given the opportunity to enter into a lottery for an iPAD or Android Tablet.

Semi-Structured Interviews. A semi-structured interview guide will be developed consisting of questions designed to obtain input on the organizational and provider factors identified as most important from the surveys. As part of the Specialty Care Evaluation project, we have developed multiple interview guides addressing most of the CFIR constructs. These questions have been used successfully thus far for four different Specialty Care initiatives; for a draft, we have selected potentially relevant questions from these previously developed guides and tailored them for this study (see Appendix). The interviews will also contain questions about which elements of the toolkit have been implemented. Potential respondents (the clinical pharmacy specialist, director of primary care, and primary care providers) will be contacted via an email invitation followed by a phone call. All interviews will be conducted by two research team members, with one conducting the interview (audio-taped) and one taking field notes. Based on our experience with the Evaluation Center and others, we will conduct approximately 5 interviews per facility for the highest and lowest performers (n=150).[61] If saturation is not achieved, we will conduct additional interviews.[61, 62]

Site Visits. Once the analyses have been completed and the key constructs identified (see analysis plan below), we will conduct site visits to the highest performing sites; we will select facilities of various levels of complexity and location. (We have budgeted for 8 visits.) The primary purpose of the site visits will be to obtain detailed information on site-level manifestation of the constructs associated with implementation success. Interviews will be conducted with the clinical pharmacy specialists, primary care providers, and clinical managers. Clinics will be visited and artifacts collected, e.g., education materials, screen shots of CPRS (using test patients) illustrating decision support, clinical reminders, and lab comments on A1c. Site visits will require 2 days by a 2-person team. We will use methods adapted from prior work conducted by colleagues with the Center for Organization, Leadership, and Management Research (COLMR).[63]

2.4.10. **Mixed Methods Analysis.** The unit of analysis for our mixed methods approach will be the medical center. Our sample size of low and high performing sites will make it impossible to conduct formal tests of hypotheses, but we can still analyze the data to obtain important information on those provider and organizational factors potentially associated with performance. The Diabetes QUERI is collecting these data across studies for a VA QUERI database of implementation findings, which will eventually allow us to perform hypothesis testing using a meta-analytic approach. As we have done in multiple studies of national implementation efforts, our analysis will be sufficient for providing useful feedback to our operational partners regarding those provider and organizational factors needing attention for improving dissemination.

Our initial analyses will focus on identifying those factors from the *provider surveys* that at least 75% of respondents identified as important or very important for affecting successful implementation of the Choosing Wisely Initiative. In our previous work with the Specialty Care Evaluation project, this percentage allows us to identify 10-12 key organizational factors on which we can focus our interview questions. We will also look at correlations between provider characteristics and performance status (low vs. high).

To analyze the *interview* data, our focus will be on coding the data according to components of the Choosing Wisely toolkit and constructs from the CFIR. Most of the interview guide questions will define specific toolkit components and CFIR constructs that will allow the researchers to efficiently collect and evaluate the appropriate data. Following each interview, two members of our research team will review interview notes. Each member of the team independently assigns the appropriate CFIR code(s) to each response. The team then seeks consensus on the codes for all responses for a given interview. Each team member then independently reviews the responses associated with each construct and assigns a 0 or 1 rating to the construct, which reflects the respondent's perception of the manifestation of the construct in the organization (0 = negative, 1 = positive). Choosing Wisely toolkit components will be assigned either a zero (absent) or 1 (present). Once this process is completed for all respondents at a given site, the team meets to assign an overall rating for each construct at the site level, considering the ratings across respondents and each respondent's role in and knowledge of the Choosing Wisely Initiative. The findings will be entered into a matrix, with sites as columns, constructs/ components as rows, and ratings (0 or 1) as the cell entries. Data will be analyzed initially using a matrix analysis approach to examine potential correlations of the ratings with performance (i.e., ratings of 1 with high performance and ratings of 0 with low performance).[64]

The matrix will also be analyzed using qualitative comparative analysis (QCA), which is a case-oriented, comparative analytic method based on set-theoretic relationships (as opposed to statistical or correlational relationships) between explanatory factors and an outcome. This approach allows for systematic cross-case comparisons across a small to intermediate number of cases, which is the situation for this study. It is useful for studying causal complexity—when different combinations of factors can lead to the same outcome, when some factors may only be causal in the presence of other factors, and when the absence of the outcome has a different causal pathway than simply the absence of factors associated with the outcome (i.e., causal asymmetry).[64-68] We will define approximately six to eight independent variables for use in the QCA. Selection of these variables will be based on the findings from the matrix analysis and will include those toolkit components and those CFIR constructs

Figure 4

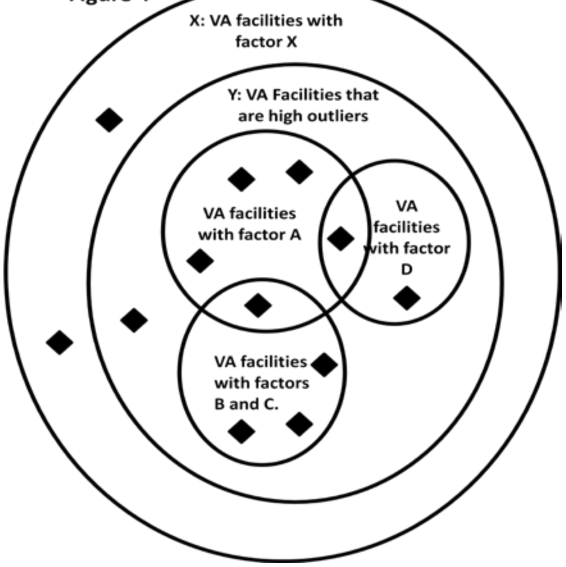

Necessary conditions are supersets of an outcome set.

- VA facilities with factor X are a superset of the outcome set (Y) of VA facilities that are high outliers.
- Factor X is necessary for being a high outlier (Y). However, having X does not guarantee outcome Y; a case can have X, but still be outside of set Y.

Sufficient conditions are subsets of an outcome set.

- VA facilities with components A, D, and the combination of B and C are subsets of the outcome set Y.

Any component (A, or D, or B & C) is sufficient for being a high outlier (Y). All VA facilities with any one of these sufficient components are within the set Y.

Note: A, B, C, D, and X can represent different toolkit components, contextual features, or other characteristic of the population, setting, or initiative. Each diamond represents a unique care (VA facility) in this example.

that (1) distinguish between low and high performers (i.e., negative or absent in low sites, positive or present in high sites); (2) were absent or negative in most sites, regardless of performance; and (3) were positive or present in most sites, regardless of performance.

QCA will allow us to determine those constructs whose positive manifestations (rating of 1) are *necessary* for achieving high performance. A construct is considered necessary if it is always positive in high-performing sites. That is, high performance cannot occur in the absence of a positive manifestation of the construct, but its presence does not guarantee high performance. QCA will also determine those constructs or combination of constructs that are *sufficient* for achieving high performance. A construct or combination of constructs is sufficient for high performance if high performance always occurs when the positive manifestation of the construct or combination of positive constructs is present. The presence of a sufficient construct or combination of constructs guarantees high performance when the necessary constructs are present. For example, we may find that a particular toolkit component is necessary, but not sufficient for high performance. Thus, a policy recommendation might be to recommend that all facilities at a minimum implement the necessary component, as without it facilities have no chance of success. We may also find that no components are necessary, but three of the available components are sufficient. Thus, a policy recommendation might be to recommend at least one of the three components, and allow facilities to select from among any of the three sufficient components. For example, among the components likely to be necessary, if not sufficient, are clinical reminders and local audit and feedback.

In addition to conducting analyses of necessity and sufficiency for the outcome of being a high performer, we will conduct similar analyses with the outcome of being a low performing facility. This is required because the causal recipe to failure can be as useful within a learning organization as that of success. Dr. L. Kahwati will provide expert consultation on the use of QCA. She has used this approach previously in determining best practices in VHA's MOVE! Weight Management Program for Veterans[69] and is PI on a current Agency for Healthcare Research and Quality-funded methods grant to assess the feasibility of using QCA to explain heterogeneity within systematic reviews of complex interventions. Once the important constructs and combinations of constructs have been identified from our analyses, we will go back to the summary memos from our interviews to obtain additional information on the specific practices at sites in which these constructs were positively manifested. In addition, data from the visits to high performing sites will be analyzed descriptively for identifying best practices, and the findings from these analyses will then become the basis of our specific recommendations for improving dissemination of the Choosing Wisely Initiative.

2.5. Contingencies, Limitations, and Other Considerations. There are several factors with significant potential impact on the project including timing and completeness of data. First, the initiative may be delayed (although according to the co-Chair, it is on schedule). Therefore, we have built in a one-year period for implementation which should accommodate a delay of up to six months. However, if necessary, we will request an extension of the project by the necessary amount of time. CMS data is likely to be delayed, but VA data alone should suffice for the key variables. As a natural experiment, we cannot predict with certainty how many facilities will choose to focus on hypoglycemic risk and sample size may be jeopardized if that number is insufficient. However, we are aware of three VISNs who have expressed interest in this project, especially because of its potential to reduce the costs associated with hypoglycemia in the elderly, e.g., hip fracture from falls. Second, the project is contingent upon obtaining data from busy providers. We are taking several approaches to this concern: (1) We have limited the surveys to a single one and minimized respondent burden as much as possible; (2) we have limited surveys to high/low performers allowing us to concentrate our recruitment efforts; (3) Office of Primary Care is strongly committed to this initiative and will promote participation in surveys; (4) PBM is strongly committed to working with clinical pharmacy specialists in the field; and (4) members of the research team, steering committee, and DM-QUERI have extensive social networks which will enable identification of individuals who can facilitate data collection. The study design is quasi-experimental using a natural experiment, so adopters and non-adopters may differ significantly at baseline. Our paired pre-post analysis for each facility based upon its implementation date will help to ameliorate this concern. We are studying a single disorder – diabetes - and findings may not generalize to other conditions. Nevertheless, DM is not only prevalent, but has been a good model for issues related to chronic disease. In addition, a key stakeholder group is not included in this proposal – patients with diabetes and their families. Anecdotally, it appears that some patients, who like clinicians, have been subjected to the marketing campaigns of the ADA, National Diabetes Education Project, and pharmaceutical companies, are reluctant to relax their levels of glycemic control. In addition, clinician-patient interactions affect the choice of targets. We omitted this important group for two reasons. First, the expanded scope of the project could not be

accomplished within budget. Second, evaluation of patient factors would be complicated by the fact that many patients at higher risk for hypoglycemic events may comprise a vulnerable population by virtue of age, cognitive impairment, and alcohol/substance use and/or serious mental conditions, which might compromise decision-making, and necessitate inclusion of family members. Therefore, we plan to submit a pilot proposal (RRP) specifically addressing patients and clinician-patient interactions. For Aim 3, the number of facilities is too large for intensive case studies of all of them, but too small for a regression approach. QCA addresses the middle ground and does so effectively. In addition, our choice of QCA was based on the fact that VHA's Choosing Wisely initiative is a complex intervention nested within a complex and open health care system, and as such issues of causal attribution are more problematic. In addition, some components may only be causal when present in association with other components (conjunctural causality). Similar outcomes may result from different causal pathways (equi-finality). Finally, we recognize that the Choosing Wisely initiative is occurring in a changing health care environment where something completely unexpected is likely to happen. We will be attentive to developments that potentially affect the initiative and be prepared to modify our procedures to take them into account.[70] For example, addition of components to the toolkit or national adoption of a performance measure or inclusion of a monitor in the PCP/PCMH dashboard during the period of implementation would necessitate modification of the surveys and interview guides. The timing would dictate whether or not modification of the time points for analyses is necessary.

3.0. Dissemination of Findings and Project Management. The dissemination plan is illustrated in Table 1 . In addition to presentations at national meetings (e.g., VA HSR&D Service meetings, Academy Health) and submission of results to peer-reviewed journals, there are other means for dissemination of the findings. The PI serves on committees in VISN10, including the Chronic Care and Health Systems Design Committees. Results, particularly those with policy implications, will be shared with these committees along with the Executive Leadership Council (which includes all the Service Line Directors and Chiefs of Staff), the Network Chief Medical Officer, and the Network Director. Moreover, our steering/advisory committee has representatives from VA HSR&D, Patient Care Services (PCS) including PBM and NCPS, as well as a Network Director and CMO. In addition to their advisory role, these committee members will provide input for effectively and efficiently disseminating relevant findings as well as presenting those findings to their respective offices. In addition to their links beyond VA, we specifically note that Dr. Pogach is PCS VA National Program Director, Diabetes. He is also a member of the DM Interagency Coordinating Committee consisting of NIDDK, CDC, DOD, AHRQ, VA, Indian Health System, and other federal

**Table 1. End Users**	**Needs**	**Means**
Researchers	Scientific findings	Publications, scientific meetings
Clinical Operations - Service/Section Chiefs	Scientific and Operations-relevant findings	Presentations at Field Advisory Committee Meetings by PI (FAC member) and P. Conlin (FAC chair)
Managers - CMOs, QMOs, VISN Directors and facility equivalents	Operations-relevant findings	Executive briefs presented by Dr. Gelman, CMO, VISN10 to CMOs and by Dr. Murawsky, Director, VISN12 to other VISN directors and National Leadership Board.
Policy – Offices of Primary Care, Specialty Care, PBM, Academic Affiliations	Operations-relevant findings	Executive briefs will be presented by representatives of these offices serving on the Steering Committee
Choosing Wisely Initiative/ Interagency Task Force	Operations-relevant findings	Executive briefs will be presented by representatives of these offices serving on the Steering Committee
Professional diabetes community – ADA, AACE, NIDDK, Endo. Society, PCORI	Primarily, scientific findings	Meeting abstracts, publications, social networking with other members of these societies/organizations
Clinicians (Primary Care Providers), Endocrinologists, Clinical Pharmacy Specialists, Certified Diabetes Educators	Clinically relevant findings	Presentations to Endocrine/Diabetes Field Advisory Committee (P. Conlin); Primary Care Work Group (S.Kirsh, M.McConnell); ONS Work Group (S.Watts); CPS Work Group (V. Torrise)

**Table 2.** Steering/Advisory Chair PCS-OSC (L. Pogach); Committee: DM-QUERI (C. Richardson); PCS-OPC and OSC (S. Kirsh) PBM (V. Torrise) VA Natl Ctr for Patient Safety(D. Hoover); Office of Acad Affiliations (S. Gilman) Ann Arbor COIN (E. Kerr)	
D. Aron (PI); Project Mgr – Sherry Ball, PhD	
Quant.Team– C-L Tseng, Dr. PH	Qual. Team– Julie Lowery, PhD
O. Soroka; TBN	D. DiFiore, PhD; L. Stevenson, PhD; M. Montpetite, MBA

agencies. He is also chair of the federal interagency work group to address adverse drug events.

3.1. Project Management Plan. The general organizational structure of the project is shown in Table 2. The steering/advisory committee will convene with the PI quarterly for the first year and then semi-annually by teleconference. We will also take an opportunistic approach and meet when there are a significant number of members present, e.g., at QUERI, VA HSR&D, or Academy Health annual meetings, as well at other national meetings for VA. Individuals will also be contacted and provide guidance as needed between scheduled meetings. The external advisory committee members were selected for their expertise in research, healthcare delivery, and training. The internal steering committee will meet every other week initially and then monthly (or more often as necessary) and will be responsible for coordinating the various sub-parts of the project. Quantitative data will be analyzed at the East Orange and Cleveland VA Medical Centers. Some data will be analyzed through VA Informatics and Computing Infrastructure (VINCI), the VA central platform for research. Qualitative data will be analyzed at Cleveland and Ann Arbor. The Quantitative Team will oversee all aspects related to data collection and management (e.g., constructing the necessary databases, coordinating data downloading and merging, overseeing the preliminary analyses). J. Lowery will direct the Qualitative Team, including coordination of interviews, coding and analyses. The Project Manager will oversee the daily activities of the project, including review of time and activity allocations, to assure its smooth and efficient operation. She will also be responsible for survey administration. She will meet weekly with the PI. During the last 6-8 months of the project, activities will focus primarily on the analysis of data, preparation of abstracts and reports, and manuscript writing (see Gantt chart below). The key facilities and personnel needed for the proposed work are available and in place: 1. Personnel. A team consisting of individuals who have been working together for many years has been formed that is fully capable of performing the research. 2. Space. Each study sites has sufficient office space for each of the investigators and research staff. 3. Computers. Existing computer facilities are more than adequate and software is already installed for database management, statistical analysis (SPSS and SAS), qualitative analyses (Atlas.ti®), and surveys (licenses for Inquisite®).

GANTT CHART

Year	**14**				**15**					**16**				**17**
month	1-3	4-6	7-9	10-12	1-3	4-6	7-9	10-12	1-3	4-6	7-9	10-12	1-3	4-6
DM-QUERI funded period														
Refine Initial Survey Instruments/ Interview Guide based on pilot testing														
Survey for participation status														
Administrative/clinical data downloading														
Outlier identification based on facility rates (for assessing sustainability)														
Outlier identification based on CHANGE in facility rates for site selection														
Quant. Analyses Implementation Phase														
Surveys/Interviews with high/low performers														
Development of qual. codes														
Site visits														
Qual. Comp. Analysis (QCA)														
Quant. Analyses Sustainability Phase														
Final Statistical Modeling, and Hypothesis Testing														
Executive Briefs														
Abstracts and manuscripts														
Final report														

Reference List

1. Bernet A, Willens D, Baurer M. Effectiveness-implementation hybrid designs: implications for quality improvement science. Implementation Science 2013;8(Suppl 1):S2.

2. Curran GM, Bauer M, Mittman B, Pyne JM, Stetler C. Effectiveness-implementation hybrid designs: combining elements of clinical effectiveness and implementation research to enhance public health impact. Med Care 2012;50(3):217-226.

3. Craig P, Petticrew M. Developing and evaluating complex interventions: reflections on the 2008 MRC guidance. Int J Nurs Stud 2013;50(5):585-587.

4. Sarkar U, Karter AJ, Moffet HH, Adler NE, Schillinger D. Hypoglycemia is more common among type 2 diabetes patients with limited health literacy: the Diabetes Study of Northern California (DISTANCE). J Gen Int Med 2010;25(9):962-968.

5. Feil DG, Rajan M, Soroka O, Tseng CL, Miller DR, Pogach L. Risk of hypoglycemia in older veterans with dementia and cognitive impairment: implications for practice and policy. JAGS 2011.

6. Barnett A, Cradock S, Fisher M, Hall G, et al. Key considerations around the risks and consequences of hypoglycaemia in people with type 2 diabetes. Int J Clin Pract 2010;64(8):1121-1129.

7. Hsu P, Sung S, Cheng H et al. Association of clinical symptomatic hypoglycemia with cardiovascular events and total mortality in type 2 diabetes: a nationwide population-based study. Diabetes Care 2013;36(4):894-900.

8. Seaquist ER, Anderson J, Childs B et al. Hypoglycemia and diabetes: a report of a workgroup of the American Diabetes Association and the Endocrine Society. Diabetes Care 2013;36(5):1384-1395.

9. Lipska KJ, Warton EM, Huang ES et al. HbA1c and Risk of Severe Hypoglycemia in Type 2 Diabetes: The Diabetes and Aging Study. Diabetes Care 2013.

10. AGS Choosing Wisely Workgroup. American Geriatrics Society Identifies Five Things That Healthcare Providers and Patients Should Question. Journal of the American Geriatrics Society 2013;http://www.americangeriatrics.org/files/documents/jgs12226.pdf:1-10.

11. Pogach L, Aron DC. Sudden acceleration of diabetes quality measures. JAMA 2011;305(7):709-710.

12. Aron D, Pogach L. Transparency standards for diabetes performance measures. JAMA 2009;301:210-212.

13. Aron D, Pogach L. One Size Does Not Fit All: A Continuous Measure for Glycemic Control in Diabetes: The Need for a New Approach to Assessing Glycemic Control. Jt Comm J Qual Improv 2007;33:636-643.

14. O'Conner P, Bodkin N, Fradkin J et al. Consensus Report: Diabetes performance measures: current status and future directions. Diabetes Care 2011;34:1651-1659.

15. Kirsh S, Hein M, Pogach L et al. Improving Outpatient Diabetes Care Delivery. Am J Med Qual 2012;27(May-June(3)):233-240.

16. Coombs C, Hislop D, Holland J, Bosley S, Manful E. Exploring types of individual unlearning by local health-care managers: an original empirical approach. Health Services and Delivery Research 2013; 1(2).

17. Rushmer R, Davies HT. Unlearning in health care. Qual Saf Health Care 2004;13 Suppl 2:ii10-ii15.

18. Cegarra-Navarro J, Wensley AK, Sanchez Polo M. A conceptual framework for unlearning in a homecare setting. Knowledge Management Research & Practice 2013.

19. Cegarra-Navarro J, Cepeda-Carrion G, Eldridge S. Balancing technology and physician–patient knowledge through an unlearning context. International Journal of Information Management 2011; 31(5):420-427.

20. Power ML, Baron J, Schulkin J. Factors associated with obstetrician-gynecologists' response to the Women's Health Initiative trial of combined hormone therapy. Med Decis Making 2008;28(3):411-418.

21. Tsai SA, Stefanick ML, Stafford RS. Trends in menopausal hormone therapy use of US office-based physicians, 2000-2009. Menopause 2011;18(4):385-392.

22. Kale MS, Bishop TF, Federman AD, Keyhani S. Trends in the overuse of ambulatory health care services in the United States. AMA Intern Med 2013;173(2):142-148.

23. Ajzen I. The Theory of Planned Behavior. Organizational behavioral and human decision processes 1991;50:179-211.

24. Godin G, Belanger-Gravel A, Eccles M, Grimshaw J. Healthcare professionals' intentions and behaviours: a systematic review of studies based on social cognitive theories. Implement Sci 2008;3:36.

25. Rashidian A, Russell I. Intentions and statins prescribing: can the theory of planned behaviour explain physician behaviour in following guideline recommendations? J Eval Clin Pract 2011;17(4):749-757.

26. Greenhalgh T, Robert G, Bate P, Peacock R, Kyriakidou O, Macfarlane F. How to Spread Good Ideas A systematic review of the literature on diffusion, dissemination and sustainability of innovations in health service delivery and organisation. University College of London: National Co-ordinating Centre for NHS Service Delivery and Organisation R & D (NCCSDO) , 2004HS&DR - 08/1201/038.)

27. Pogach L, Conlin PR, Hobbs C, Vigersky RA, Aron D. VA-DoD Update of Diabetes Guidelines: What Clinicians Need to Know About Absolute Risk of Benefits and Harms and A_{1c} Laboratory Accuracy. Federal Practitioner 2011;(April):39-44.

28. Laiteerapong N, John PM, Nathan AG, Huang ES. Public health implications of recommendations to individualize glycemic targets in adults with diabetes. Diabetes Care 2013;36(1):84-89.

29. Bennett WL, Odelola OA, Wilson LM et al. Evaluation of guideline recommendations on oral medications for type 2 diabetes mellitus: a systematic review. Ann Intern Med 2012;156(1 Pt 1):27-36.

30. Inzucchi SE, Bergenstal RM, Buse JB et al. Management of hyperglycaemia in type 2 diabetes: a patient-centered approach. Position statement of the American Diabetes Association (ADA) and the European Association for the Study of Diabetes (EASD). Diabetologia 2012;55:1577-1596.

31. Ismail-Beigi F, Moghissi ES, Tiktin M, Hirsch IB, Inzucchi SE, Genuth S. Individualizing Glycemic Targets in Type 2 Diabetes Mellitus: Implications of Recent Clinical Trials. Annals Internal Medicine 2011;154(April 19):554-559.

32. Pogach L, Aron. The other side of quality improvement in diabetes for seniors: a proposal for an overtreatment glycemic measure. Arch Int Med 2012;172(19):1510-1512.

33. Woodard LD, Landrum CR, Urech TH, Profit J, Virani SS, Peterson LA. Treating Chronically Ill People with Diabetes Mellitus with Limited Life Expectancy: Implications for Performance Measurement. JAGS 20122;60(2):193-201.

34. Pogach LM, Rajan M, Maney M, Tseng CL, Aron DC. Hidden complexities in assessment of glycemic outcomes: are quality rankings aligned with treatment? Diabetes Care 2010;33(10):2133-2139.

35. Pogach L, Tiwari A, Maney M, Rajan M, Miller M, Aron D. Should Mitigating Comorbidities be considered in assessing Healthcare plan performance in achieving optimal glycemic control? Am J Managed Care 2007;13(3):133-140.

36. Prasad V, Vandross A, Toomey C et al. A decade of reversal: an analysis of 146 contradicted medical practices. Mayo Clin Proc 2013;88(8):790-798.

37. Tseng CL, Soroka O, Maney M, Aron DC, Pogach LM. Assessing Potential Glycemic Overtreatment in Persons at Hypoglycemic Risk. JAMA Intern Med 2013.

38. Porter M. Value in Health Care. N Engl J Med 2010;363:2477-2481.

39. Porter M, Lee T. The Strategy That Will Fix Health Care. Harvard Business Review 2013;(October).

40. Pawson R, Greenhalgh T, Harvey G, Walshe K. Realist synthesis: an introduction. Research Methods Programme, University of Manchester, RMP Methods Paper 2004.

41. York L, Bruce B, Luck J et al. Online toolkits for metric-driven quality improvment: the Veterans Health Administration managed grassroots approach. Joint Commission Journal on Quality and Patient Safety 2013;39(12):561-569.

42. Creswell JW. Research Design: Qualitative, Quantitative, and Mixed Methods Approaches. 2nd ed. Thousand Oaks, CA: Sage Publications Inc.; 2003.

43. Creswell JW, Klassen A, Plano Clark V, Clegg Smith K. Best Practices for Mixed Methods Research in the Health Sciences. Office of Behavioral and Social Sciences Research (OBSSR), 2013

44. Becker K. Facilitationg unlearning during implementation of new technology. Journal of Organzational Change Managment 2013; 23(3):251-268.

45. Tjia J, Velten SJ, Parsons C, Valluri S, Briesacher BA. Studies to reduce unnecessary medication use in frail older adults: a systematic review. Drugs Aging 2013;30(5):285-307.

46. Collins C, Limone BL, Scholle JM, Coleman CI. Effect of pharmacist intervention on glycemic control in diabetes. Diabetes Res Clin Pract 2011;92(2):145-152.

47. Scherbaum CA, Ferreter JM. Estimating statistical power and required sample sizes for organizational research using multilevel modeling. Organizational Research Methods 2009;12(2):347-367.

48. Raudenbush SW, Martinez A, Spybrook J. Strategies for Improving Precision in Group-Randomized Experiments. Educational Evaluation and Policy Analysis 2007;29(1):5-29.

49. Spybrook J, Raudenbush SW, Liu X, Congdon R, Martinez A. Optimal Design for Longitudinal and Multilevel Research: Documentation for the "Optimal Design" Software. 58. 2008.

50. Spybrook J, et.al. Optimal Design for Longitudinal and Multilevel Research: Documentation for the Optimal Design. Software Version 3.0. Available from www.wtgrantfoundation.org. 2011. Software Version 3.0. Available from www.wtgrantfoundation.org.

51. Zou G, Donner A. Confidence Interval Estimation of the Intraclass Correlation Coefficient for Binary Outcome Data. Biometrics 2004;60:807-811.

52. Polanczyk C, Lane A, Coburn M, Philbin E, Dec E, DiSalvo T. Hospital outcomes in major teaching, minor teaching, and nonteaching hospitals in New York state. Am J Med 2002;112(4):255-261.

53. Allison J, Kiefe C, Weissman N et al. Relationship of Hospital Teaching Status With Quality of Care and Mortality for Medicare Patients With Acute MI . JAMA 2000;284(10):1256-1262.

54. Kupersmith J. Quality of care in teaching hospitals: a literature review. Acad Med 2005;80:458-466.

55. Tseng C. Seasonal patterns in monthly hemoglobin A1c values. Am J Epidemiol 2005;161(6):565-574.

56. Meredith LlRi. Veterans Assessment and Improvement Laboratory (VAIL) VISN 22 Survey of Primary Care Team Experiences. 1-2-2014.

57. Chin MH, Cook S, Drum ML et al. Barriers to providing diabetes care in community health center. Diabetes Care 2001;24(2):268-274.

58. Glazier RH, Dalby DM, Badley EM, Hawker GA, Bell MJ, Buchbinder R. Determinants of physician confidence in the primary care management of musculoskeletal disorders. J Rheumatol 1996;23(2):351-356.

59. Parchman ML, Pugh JA, Romero RL, Bowers KW. Competing demands or clinical inertia: the case of elevated glycosylated hemoglobin. Ann Fam Med 2007;5(3):196-201.

60. Damschroder L, Aron D, Keith R, et al. Fostering implementation of health services research findings into practice: A consolidated framework for advancing implementation science. Implementation Science 2009;4(1ss).

61. Taliani CA, Bricker P, Adelman A, Cronholm P, Gabbay R. Implementing Effective Care Management in the Patient-Centered Medical Home. Am J Manag Care 2013;Published Online: December 12, 2013.

62. Francis J, Johnston M, Robertson C et al. What is an adequate sample size? Operationalising data saturation for theory-based interview studies. Psychology & Health 2010;25(10):1229-1245.

63. Wrobel J, Charns M, Dieher P et al. The Relationship Between Provider Coordination and Diabetes-Related Foot Outcomes. Diabetes Care 2003;26(11):3042-3047.

64. Averill J. Matrix analysis as a complementary analytic strategy in qualitative inquiry. Qual Health Res 2002;12:855-866.

65. Blackman T, Wistow J, Byrne D. Using Qualitative Comparative Analysis to understand complex policy problems. Evaluation 2013; 19(2):126-140.

66. Ragin CC. Using qualitative comparative analysis to study causal complexity. Health Serv Res 1999;34(5 Pt 2):1225-1239.

67. Schneider C, Wagemann C. Standards of good practice in qualitative comparative analysis (QCA) and fuzzy-sets. Comparative Sociology 2010; 9(3):397-418.

68. Vis B. The comparative advantages of fsQCA and regression analysis for moderately large-N analyses. Sociological Methods & Research 2012;41(1):168-198.

69. Kahwati LC, Lewis MA, Kane H et al. Best practices in the Veterans Health Administration's MOVE! Weight management program. Am J Prev Med 2011;41(5):457-464.

70. Glasgow RE. What does it mean to be pragmatic? Pragmatic methods, measures, and models to facilitate research translation. Health Educ Behav 2013;40(3):257-265.
