# Supplementary material for: De-implementation of inappropriately tight control (of hypoglycemia) for health: protocol with an example of a research grant application
Source: Implement Sci. 2014 May 19;9:58. doi: 10.1186/1748-5908-9-58 (PMC4046046; doi:10.1186/1748-5908-9-58)
Supplement: Additional file 2 — Concept paper. [file 1748-5908-9-58-S2.docx]

**Project Title:** De-implementation of Inappropriate Tight Control for Health

**Objectives and clinical focus.** Two important issues in healthcare are (over)use of low value practices and medication safety. The former is the target of the Choosing Wisely Campaign, while the latter is the focus of several initiatives both in and out of VHA. These two issues intersect in overtreatment of diabetes (DM), i.e., intensive glycemic control in clinical situations without substantial benefits but with increased risk for hypoglycemia.^1^ VHA provides care to >8 million veterans; approximately 20-25% of patients have DM. Hypoglycemia is common, underestimated and serious, especially in patients treated with insulin and/or sulfonylurea drugs. Hypoglycemia is associated with morbidity, mortality, and increased healthcare costs.^2-12^ As a consequence of landmark clinical trials (ACCORD, ADVANCE, and VADT), increasing attention is now being paid to the risks and the need to individualize glycemic targets.^13-15^ For >15 years the VA/DoD DM Clinical Practice Guidelines (CPG) included targets stratified by life expectancy and diabetes complications, but their wholesale adoption has been hampered,^16^ in part, due to strong marketing by professional societies and pharmaceutical manufacturers of other guidelines, e.g. American Diabetes Association (ADA), that promoted a single A1c<7% target).

We have developed a measure of “potential over-treatment” operationally defined as: A1c<7%, treatment with insulin or sulfonylurea and one of the following (a)>70 years of age, (b) serum creatinine >1.7 mg/dl or (c) cognitive impairment or dementia. This measure is considerably more restrictive than one of the recommendations of the *Choosing Wisely Campaign* which is “Avoid using medications to achieve hemoglobin A1c <7.5% in most adults age 65 and older; moderate control is generally better.”^17^ Nevertheless, our data indicate that up to 30% of patients in VA are being overtreated. These issues have led to an interagency task force (VHA, HHS, CDC, DoD, NIH-NIDDK) to address hypoglycemia. In VA Pharmacy Benefits Management (PBM) supported by the Office of Specialty Care have begun an initiative to reduce overtreatment of diabetes. The goal of our project is to utilize this natural experiment to study the concomitant processes of de-implementation of the practice of achieving tight glycemic control when clinically inappropriate and implementation of hypoglycemia risk reduction.

**Proposed quality improvement project. *Multi-faceted Hypoglycemia Risk Reduction Intervention (MHRRI).*** This national project sponsored by PBM uses clinical pharmacists to promote appropriate de-intensification of glycemic control, targeting primary care teams and their patients. The intervention components in addition to educational materials include the following six elements: (1) clinical reminder; (2) CPRS-based decision support; (3) academic detailing; (4) clinical champions; (5) audit and feedback; and (6) system redesign for multidisciplinary organization of care.^18^ Each of these evidence-based elements has empirical support for its effectiveness in changing practice. The Theory of Healthcare Professionals’ Behavior and Intention, a modification of the Theory of Planned Behavior provides theoretical support.^19, 20^ The multifaceted approach allows for local adaption which has been shown to be important in similar efforts, e.g., guideline implementation.^18^ However, although some might refer to this approach as an intervention bundle, it has not been clearly shown that effectiveness requires that all elements be implemented.^21^ Nevertheless, all of the elements have been implemented in several VISN12 facilities and shown to be effective. Rates of assessment for hypoglycemia have increased markedly and there were management changes (relaxation of glycemic control, e.g., by discontinuation of insulin and sulfonylureas) in >40% among those reporting hypoglycemia , (see data in power calculation below)

|  |
| --- |

**Evaluation design and methods** -*Background.* PBM has been training Clinical Pharmacists/Clinical Pharmacy Specialist (CP/CPS). As of 3/2013 there were 1,527 pharmacists in 131 medical centers authorized to prescribe in Primary Care and/or Diabetes clinics. We will utilize an iterative mixed methods approach to assess implementation, effectiveness and factors associated with rates of de-intensification of glycemic control.

*Aims and Methods*

**Aim 1.** Assess the implementation and impact of the Multifaceted Hypoglycemia Risk Reduction Intervention on rates of overtreatment of diabetes.

**Aim 2**: Identify factors associated with successful reduction of rates of overtreatment.

**Aim 3**: Assess the de-implementation of achieving tight glycemic control when clinically inappropriate from a clinician’s perspective.

We will conduct a prospective mixed-methods evaluation employing multiple integration approaches.^22^ This approach combines cross-sectional interviews and surveys of administrators, primary care, and specialty care providers, and analysis of rates of overtreatment and measures of intervention implementation.

**Aim 1.** Assess the implementation and impact of the Multifaceted Hypoglycemia Risk Reduction Intervention on rates of overtreatment of diabetes.

*Overview*: We will conduct a summative evaluation by assessing the rates of overtreatment in facilities that implement one or more elements of the intervention compared to structurally similar control VHA sites. We will also conduct a formative evaluation of intervention implementation And then survey VA facilities to assess intervention implementation timelines and intervention intensity/ fidelity.

*Endpoint variable*. (1) Overtreatment measure; and (2) medication use, especially insulin, sulfonylureas and new agents associated with low frequency of hypoglycemia. We will also assess occurrence of hypoglycemia as measured by ICD-9 codes, but recognize the insensitivity of administrative data and that coding accuracy may change with the intervention. Data on potential overtreatment will be obtained from VHA national administrative and clinical data. For the formative evaluation, the primary endpoint variable is.intervention intensity/fidelity. Intervention intensity or implementation (fidelity) is defined as the number of elements of the HRRB implemented. These data will be obtained by an online survey of all clinical pharmacy specialists and interviews with key informants at VA facilities (medical centers and CBOCs). This will complement internal PBM progress reports. Because this is a national initiative, we will structure the evaluation using the RE-AIM framework.  This commonly used population health impact framework addresses Reach, Effectiveness, Adoption, Implementation, and Maintenance. *Reach* refers to the participation rate among eligible individuals (% of patients on insulin and or sulfonylurea seen in sites with CP/CPSs. *Effectiveness* refers to the outcome measures at the patient level (overtreatment rate) and facility setting *Adoption* refers to the participation rate among possible settings and the representativeness of settings participating. *Implementation* refers to the extent that intervention was delivered as intended (number of intervention elements implemented). *Maintenance* refers to longer term outcomes, e.g., >12 months at the individual level as well as the impact on intervention attrition at the setting level.

Analysis. All analyses will be conducted at the level of medical centers, with CBOCs being evaluated separately versus not). Below we use “facility” to simply refer to the health care unit being analyzed. For our primary analysis, we will calculate the number of patients exceeding A1c thresholds of <6.0%, <6.5%, and <7.0% (i.e., the numerators) of defined high risk patients (i.e., the denominator) and obtain the overtreatment rates (=100%*numerator/denominator) on a monthly (alternatively, quarterly) basis. To compare whether the mean rate differ among the different HRRB intensity groups, we will used a random effects model (also termed Hierarchical or multilevel models). We will model the number of events (i.e., counts of patients in a facility exceeding an A1c threshold) as the dependent variable and assume it follows a Poisson distribution. Independent variables to be entered into the models include Intervention indicator (indicating the HRRB elements and pre-intervention), implementation order of all interventions in a facility, and number of patients in the denominator (facility and month specific). In the random effect models, we will treat facilities as random effects, because the overtreatment rates from different months are repeated measurements within a facility hence are correlated. It is important to accommodate the correlation of the data into statistical modeling since conventional statistical models will results in biased estimate of the variance of the regression coefficients, hence possibly invalid conclusions. In the model, we will evaluate HRRB intensity as a categorical as well as a continuous measure. In the latter approach, we will be able to evaluate the trend of overtreatment in relation to increasing number of HRRB intensity elements (range: 0-6). Facility and VISN level variables can be added to the models to allow adjustment of facility and VISN differences in patient mix/composition.

In this study, we are especially interested in evaluating whether use of the clinical reminder is significantly associated with changes in the rates of overtreatment. Using the above analysis, the subgroup contrasts between the clinical reminder and the other interventions will provide answers to such question. However, we recognize the above strategy allows only comparisons of averages across time among intervention groups and does not provide information such as whether the improvement in the overtreatment rate is in the absolute value (i.e., the intercept) or the in the trend (i.e., slope) or both. In an attempt to achieve this, we propose to use the approach of interrupted time series and construct random-effects segmented regression models. As aforementioned, the inclusion of random effects (facilities) will address the issue of correlation of the repeated measurements (the time series data) within a facility. The same dependent variable will be modeled based on a Poisson distribution. A time indicator (in months; in reference to the implementation of the clinical reminder) will be included in the models. Based on the empirical data as guided by graphing time series of monthly overtreatment rates from 2011 to 2015, we will evaluate models with polynomials of time (calculated in reference to the time when the clinical reminder is implemented) to allow non-linear trends. Facility-level variables such as patient compositions, implementation of other intervention elements as well as other variables that may affect glycemic control will be included in the models. A similar approach will be used for medication usage and occurrence of coded hypoglycemia.

Power analysis: We conducted power calculation based on Type I error rate 0.05 for two-sided tests and used preliminary data in which we identified > 285,400 (about 25.8% of patient with diabetes) patients across 131 medical centers in FY2009 with insulin/sulfonylurea who were >=75 years old, or with either diagnosis of dementia or cognitive impairment, or having serum creatinine >1.7 mg/dl. Of them, 10.5% had last A1c value <6%, 27.1% less than 6.5%, and 48.4%% less than 7%. Work from VISN 12 shows that the rate of A1c<7% reduced from 34.5% in March 2012, to 32.9% in September 2012, and further to in March 2013.

We calculated effective sample size (ESZ) using the formula below: ESZ = (m*K)/(1+ρ*(m-1)), where m=# of repeated measurements (i.e., number of monthly data per cluster), k=number of clusters (131 medical centers) , and ρ=estimate of Intra-cluster correlation. The ESZ are 1462 and 1714 for 24 and 36 repeated measurements, respectively. Based on simple Poisson regression, these sample sizes would provide 0.75 and 0.81 power to detect a rate ratio as small as 87% (e.g. 30%/34% or 48%/41.8% for A1c <7.0%, or 23.5%/27% for A1c <6.5%, or 9.6%/11% for A1c <6.0%).

**Aim 2**: Identify factors associated with successful reduction of rates of overtreatment.

*Overview*. We will use the Consolidated Framework for Implementation Research (CFIR) framework to conduct a formative evaluation of implementation at a sample of the sites involved in the HRRB, to identify the factors contributing to successful implementation.^23^ Data from Aim 1 will be used to identify high, mid, and low performing sites based on change in potential overtreatment rates for purposive sampling for in-depth interview key informants. All interviews will be conducted by two research team members, with one conducting the interview (audio-taped) and one taking field notes (written or typed). We will use a rapid analytic process (see below) so that we can incorporate emergent findings to inform data collection for subsequent interviews, e.g., adapting the interview guide. We will conduct key informant interviews at two points in time in order to assess changes in barriers and facilitators, and the de-implementation process. We will also record and transcribe interviews for later, more-detailed inductive qualitative analyses. The qualitative findings will then be used to inform a broader quantitative survey. In this case, preliminary findings from in-depth interviews are used to develop structured survey questions (hence a modified sequential approach). Most of the interview guide questions will define specific CFIR constructs that allow the researchers to efficiently collect and evaluate the appropriate data. Following each interview, the research team reviews interview notes. The two member team then evaluates the fidelity to the predefined CFIR construct using a categorical rating of each element on a simple ordinal scale in terms of perceived impact on implementation. The evaluation document includes a field for summary notes on each CFIR element to allow for emergent findings (i.e., themes not anticipated). For overall site evaluations, all team members involved with the site interviews will meet and analyze the site rating by consensus of the merged individual ratings. In addition, we will use an iterative, deductive and inductive toolkit of analytical strategies with a focus on Content Analysis to identify emergent codes and themes of unidentified factors and relationships between specific constructs and implementation process patterns outside of the defined implementation constructs. Data will be catalogued using Atlas-ti software system where upon the data cleaning and quality assurance process occur to assess the following: protocol adherence to process, fidelity to the taped interview, and detection of leading questions. This is the approach we have used in the HSR&D/Office of Specialty Care(OSC) Evaluation Centers.

The HRRB implementation is a complex intervention nested within a complex and open health and social systems. Issues of causal attribution become more problematic. In fact, experimental, positivist methodologies such as those in Aim 1, which narrowly focus on the outcomes of interventions, are inadequate to deal with this complexity. They address the question of “what works” but may be inadequate to help answer the question: “what is it about this program that works for whom in what circumstances”, in other words: “which mechanisms cause which outcome under which circumstances.”^24^ Moreover, despite the national scope of the initiative, the heterogeneity within the interventions necessitates an approach tailored for the “problem of small n’s,” making causal attribution even more problematic. That is, causal attribution is based on being able to create, locate, or simulate a counter-factual – i.e. an estimate of what would have happened in the absence of the intervention. However, when interventions are complicated (consisting of many components, all of which are needed to produce the impacts) or complex (evolving and emergent), it is not possible or appropriate to develop an explicit counter-factual. Whether impossible (or unreliable as in the case of small n problems), analysis should address causal *contribution* rather than *attribution*.^25, 26^ Contribution Analysis is a theory-based approach which sets out to demonstrate a plausible association between a program and observed outcomes. Based on explicit criteria and using weight of evidence, a credible contribution story is constructed in which each step lying between program inputs and outcomes is clearly evidenced, with the result that a ''reasonable person, knowing what has occurred in the program and that the intended outcomes actually occurred, agrees that the program contributed to these outcomes.' ^25, 26^ Where interventions are more complex, involving numerous sub-elements or simultaneous causal strands or where, for example, causality is recursive or outcomes emergent as is the case with the PBM initiative, multiple causal strands may be developed and each of these summarized within a general theory of change which incorporates them all. ^25, 26^ This approach will help tease out the relative contributions of different initiatives in order to inform policy makers about which interventions are more effective.

**Aim 3**: Assess the de-implementation of achieving tight glycemic control when clinically inappropriate, from a clinician’s perspective.

The decision to de-intensify glycemic control is the obverse of the decision to intensify. There is substantial literature on clinical inertia and the failure to intensify. O’Connor et al. divided the decisionmaking failures of clinical inertia into three categories: (1) goal-related pathologies related to goals continually shifting over time or goal fixation where decisions are made based on goals with which the decisionmaker is most familiar (and most comfortable), even though they may be inappropriate; (2) faulty control strategy, e.g., one that fails to reflect not only current states, but future states as well; and (3) faulty control actions, e.g., inaccurate threshold for action or wrong action.^27^ Each of these fits with our conceptual model and is potentially targeted by the intervention. In this aim we will explore using focus groups of PCPs and in depth interviews to elucidate the issues of de-intensification of therapy with a goal of developing a quantitative survey that would facilitate targeted interventions.

4. Partnerships. Operations partners include Pharmacy Benefits Management (PBM) and Office of Specialty Care. This work will support VHA’s response (led by PBM – V. Torrise) to recommendations of a Health and Human Services National Action Plan for hypoglycemic safety. DM QUERI supports the project.

**5. Personnel:** D. Aron, MD, MS (PI), endocrinologist, established implementation researcher, former DM-QUERI Co-Clinical Coordinator and now Exec. Committee member also Directs of one of the two HSR&D/OSC Eval. Centers; P. Conlin, MD (Co-I) is an established health services researcher, endocrinologist, Chief, Medical Service at the VA Boston Healthcare System, Chair, Endocrinology/Diabetes Field Advisory Committee for OSC, and Chair Executive Committee, DM QUERI; S. Kirsh, MD, MPH (Co-I), DM-QUERI Co-Clinical Coordinator, is a health services researcher, general internist and Clinical Lead for OSC initiatives and consultant to PCS for chronic disease; M. McConnell, MD (Co-I) is a general internist, developer of the HRRB, and leader of the initiative for VISN 12; L. Pogach, MD, MBA (Co-I), DM-QUERI Co-Clinical Coordinator is an established health services researcher, endocrinologist and VA National Program Director; Chin-lin Tseng, PhD (Co-I) is a biostatistician/health services researcher. Along with Dr. Pogach, she will lead the quantitative evaluation group; Julie Lowery, PhD (Co-I), DM-QUERI Co-Implementation Coordinator is highly experienced in qualitative methods will lead the qualititative evaluation group

**Proposed Budget -**  We are requesting a budget of $1.1 million for a three year project.

Reference List

1. Pogach L, Aron. The other side of quality improvement in diabetes for seniors: a proposal for an overtreatment glycemic measure. Arch Int Med 2012;172(19):1510-1512.

2. Bonds DE, Miller ME, Dudl RJ et al. Severe hypoglycemia symptoms, antecedent behaviors, immediate consequences and association with glycemia medication usage: Secondary analysis of the ACCORD clinical trial data. BMC Endocr Disord 2012;12(May 30):5.

3. Feil DG, Rajan M, Soroka O, Tseng CL, Miller DR, Pogach L. Risk of hypoglycemia in older veterans with dementia and cognitive impairment: implications for practice and policy. JAGS 2011.

4. Holstein A, Patzer OM, Machalke K, Holstein JD, Stumvoll M, Kovacs P. Substantial increase in incidence of severe hypoglycemia between 1997-2000 and 2007-2010. Diabetes Care 2012;35(May):972-975.

5. Sarkar U, Karter AJ, Moffet HH, Adler NE, Schillinger D. Hypoglycemia is more common among type 2 diabetes patients with limited health literacy: the Diabetes Study of Northern California (DISTANCE). J Gen Int Med 2010;25(9):962-968.

6. Wright A, Cull C, MacCloud KM, Holman RR, for the UK Prosepctive Diabetes Study (UKPDS) Group. Hypoglycemia in Type 2 diabetic patients randomized to and maintained on monotherapy with diet, sulfonylurea, metformin, or insulin for 6 years from diagnosis: UKPDS73. J Diabetes Complications 2006;20(6):395-401.

7. Zoungas S, Patel A, Chalmers J et al. Severe hypoglycemia and risks of vascular events and death. N Engl J Med 2010;363(October 7):1410-1418.

8. Budnitz DS, Lovegrove MC, Shehab N, Richards CL. Emergency hospitalizations for adverse drug events in older Americans. N Engl J Med 2011;365(21):2002-2012.

9. Leese G, Wang J, Broomhall J, et al. Frequency of severe hypoglycemia requiring emergency treatment in type 1 and type 2 diabetes: a population-based study of health service resource use. Diabetes Care 2003;26:1176-1180.

10. Lundkvist J, Berne C, Bolinder B, Jonsson L. The economic and quality of life impact of hypoglycemia. Eur J Health Econ 2005;6:197-2002.

11. Shorr R, Lonneke V, Resnick H, et al. Glycemic control of older adults with type 2 diabetes: findings from the Third National Health and Nutrition Examination Survey 1988-1994. J Am Geriatr Soc 2000;48 :264-267.

12. UK Hypoglycaemia Study Group. Risk of hypoglycaemia in types 1 and 2 diabetes: effects of treatment modalities and their duration. Diabetologia 2007;50:1140-1147.

13. Pogach L, Aron DC. Sudden acceleration of diabetes quality measures. JAMA 2011;305(7):709-710.

14. Kirsh S, Aron D. Choosing targets for glycaemia, blood pressure and low-density lipoprotein cholesterol in elderly individuals with diabetes mellitus. Drugs & Aging 2011;28(12):945-960.

15. Lee SJ, Eng C. Goals of glycemic control in frail older patients with diabetes. JAMA 2011;305(April 6):1350-1351.

16. Pogach L, Conlin PR, Hobbs C, Vigersky RA, Aron D. VA-DoD Update of Diabetes Guidelines: What Clinicians Need to Know About Absolute Risk of Benefits and Harms and A_1c_ Laboratory Accuracy. Federal Practitioner 2011;(April):39-44.

17. AGS Choosing Wisely Workgroup. American Geriatrics Society Identifies Five Things That Healthcare Providers and Patients Should Question. Journal of the American Geriatrics Society 2013;<http://www.americangeriatrics.org/files/documents/jgs12226.pdf:1-10>.

18. Grol R, Grimshaw G. From best evidence to best practice: effective implementation of change in patients' care. Lancet 2003;362:1225-1230.

19. Godin G, Bélanger-Gravel A, Eccles M, Grimshaw J. Healthcare professionals' intentions and behaviours: A systematic review of studies based on social cognitive theories. Implementation Science 2008;3:36.

20. Ajzen I. The Theory of Planned Behavior. Organizational behavioral and human decision processes 1991;50:179-211.

21. Resar R, Griffin F, Haraden C, Nolan T. Using Care Bundles to Improve Health Care Quality. 2012. Cambridge, Massachusetts, Institute for Healthcare Improvement. IHI Innovation Series white paper. 5-5-2013.

Ref Type: Online Source

22. Leech NL, Dellinger AB, Brannagan KB, Tanaka H. Evaluating mixed research studies: a mixed methods approach. J Mixed Methods Res 2010;4(1):17-31.

23. Damschroder L, Aron D, Keith R, et al. Fostering implementation of health services research findings into practice: A consolidated framework for advancing implementation science. Implementation Science 2009;4(1ss).

24. Pawson R, Greenhalgh T, Harvey G, Walshe K. Realist synthesis: an introduction. Research Methods Programme, University of Manchester, RMP Methods Paper 2004.

25. Lemire S, Nielsen S, Dybdal L. Making contribution analysis work: A practical framework for handling influencing factors and alternative explanations. Evaluation 2012;18(3):294-309.

26. Mayne J. Contribution analysis: Coming of age? Evaluation 2012;18(3):270-280.

27. O'Connor P, Speril-Hillen JM, Johnson P, Rush R, Biltz G. Clinical Inertia and Outpatient Medical Errors. In: AHRQ, editor. Advances in Patient Safety: From Research to Implementation-Vol. 2. <http://www.ahrq.gov/professionals/quality-patient-safety/patient-safety-resources/resources/advances-in-patient-safety/index.html> ed. 2005:293-308.
