# Supplementary material for: De-implementation of inappropriately tight control (of hypoglycemia) for health: protocol with an example of a research grant application
Source: Implement Sci. 2014 May 19;9:58. doi: 10.1186/1748-5908-9-58 (PMC4046046; doi:10.1186/1748-5908-9-58)
Supplement: Additional file 3 — Critiques of the concept paper. [file 1748-5908-9-58-S3.docx]

**Aron Concept paper critiques**

**Review 1**

The implementation portion of this is very sophisticated compared to the average one of these I read.

The one crucial aspect of this is that they will need to strongly justify the rationale for de-intensification for patients who are on a stable dose and doing well – no symptomatic hypoglycemia. So, one of the first maxims of primary care is “don’t rock the boat” if the patient is stable. So if a 70 year old patient has an A1c of 6.9 and is on 5 mg or 10 mg of a sulfonylurea and having no symptomatic hypoglycemia episodes, are the data sufficiently strong that they will have better outcomes if the sulfonylurea is reduced or discontinued and the A1c drifts up to 7.3 or 7.5? I don’t believe that was the message of ACCORD. I believe the message of ACCORD was that if that patient’s A1c was 8, then intensifying treatment to try and get below 7 was worse than a more lenient A1c target. That’s not the same as saying someone already at an A1c of less than 7 and doing well will be harmed by being allowed to stay on that dose of hypoglycemic medication, and thus should be de-intensified. Overcoming this conceptual issue is crucial to acceptance of the premise, and acceptance by the clinicians of the goal.

Something for the investigators to think about is baseline measurement of contexts at the implementation sites. In addition to their CFIR-driven post-implementation evaluation, they would move this field along by postulating a priori the directionality of effect of a few contexts they consider key – institutional patient safety culture, leadership commitment, local esteem of the clinical champions, whatever. Then measure these at baseline, and perhaps once or twice more during the study. Then use these to test their a priori hypotheses. Ultimately, this whole context work has to lead to prospective implementation utility, meaning that we know something about the contexts before we implement the intervention, and in sites where the evidence indicates that success will be difficult to achieve because of something about the contexts, either there is a pre-implementation intervention to improve the context, or we pick a different (or modified) intervention for the particular context. Doing this all retrospectively has the same conceptual problems that a retrospective cohort does when compared to a prospective cohort.

**Review 2**

Comments on main objective: Study the process of de-implementation of the practice of achieving tight glycemic control when clinically inappropriate and implementation of hyplglycemia risk reduction.

This seems an appropriate problem to investigate with QUERI dollars – the problem is described as prevalent and having serious consequences, there is an operations partner (or two) and the interventions being examined are in common use – it would be nice to have some information on whether they work.

The first two aims seem closely related but the third is related only by topic. It could be done or not done with no loss to the others. It seems risky to include it, but it is clinically relevant and one could argue it would be helpful if the HRRB does not work. It just seems likely to invite an entirely new set of reviewer criticisms that might drag down the rest of the proposal.

Comments on approach:

Natural experiment will need to be described in a lot of detail. The more you can convince the reviewer that it is actually going on, but that it has not gone on so far that you are doing a history project, the better. It will probably also help the reviewer if you have some data on the approximate appearance of the implementation bundle at various sites (e.g., “”33 sites did intervention A, 47 did intervention B”, etc.; or maybe: “73 did one thing, 16 did 2, 12 did 3, none diid 4, 2 did 5 and 33 did 6”). But this should not be a reason to not do the project – you will just have to discuss what you expect if you don’t have any data.

I won’t comment on the statistical analysis – it is clear your team recognizes that it will be essential to have it be correct, and that it will be complex.

You do need to have a good reason for why you decide to characterize intensity by count for your primary analysis.

I think your methodologic challenges will be in defining the independent variable(s) – what does it mean to have a clinical champion? Can the clinical reminder be modified? What if the clinical champion is identified in March, the decision support is made available in April and A&F starts in May. Then the decision support is improved in July – that is, it goes from being an option on the tools menu to a link that appears whenever a new insulin order is entered on an outpatient. And I can only guess what “system redesign for multidisciplinary organization of care” means. Again, these are simply things to consider, not reasons to not submit a proposal.

Seems expensive for analyzing the results of a natural experiment. You will need to justify the number of places from which you will gather in person data on what intervention happened, which is a likely cost driver.

I would wonder if retrospective assessment of CFIR constructs is reliable. I imagine if the intervention works at a site, everyone will remember things differently than if it does not. With that limitation, is it even worth trying to do the qualitative part of the study – i.e., Aim 2. At the least you need to explain why this won’t make any difference, or that you will be able to get people to recall just how things really happened, even if their implementation approach was responsible for a failure. I am not clear on the interviews at two points in time, since both points will be after the implementation has happened.

**Review 3**

1) Aim 1 proposes to evaluate the impact of the Multifaceted Hypoglycemia Risk Reduction Intervention on overtreatment of diabetes. For these analyses, the analytic strategy is reasonable and the outcome definition and data are strong. My main concern is the quality of the predictor variables, namely the use, intensity and fidelity of the intervention components. “These data will be obtained by an online survey of all clinical pharmacy specialists and interviews with key informants at VA facilities (medical centers and CBOCs). This will complement internal PBM progress reports.” Online surveys of clinical staff usually yield terrible response rates. Furthermore, self-reports of clinical activity should be verified where possible with independent data sources. Much more detail should be provided in the full proposal so the adequacy of the data can be judged.

2) The description of Aim 2 is eloquent regarding the qualitative methods, but does not mention the number of planned interviews or the specific CFIR constructs that will be examined.

3) I don’t understand Aim 3, especially its stated purpose to develop a “quantitative survey that would facilitate targeted interventions.”

4) Overall, the concept paper convinces me that the investigators have chosen an important topic and that they will put forward a thoughtful proposal.
