# Supplementary material for: De-implementation of inappropriately tight control (of hypoglycemia) for health: protocol with an example of a research grant application
Source: Implement Sci. 2014 May 19;9:58. doi: 10.1186/1748-5908-9-58 (PMC4046046; doi:10.1186/1748-5908-9-58)
Supplement: Additional file 4 — Initial submission. [file 1748-5908-9-58-S4.pdf]

1. Specific Aims – Critical issues of overuse of low value practices and medication safety intersect in overtreatment of diabetes (DM). In particular, (over)intensive glycemic control increases hypoglycemia risk and its associated morbidity and mortality without providing meaningful benefits for certain patient groups. Our work indicates that among patients with diabetes who are at high risk for hypoglycemia – due to use of hypoglycemic agents, age, and/or significant comorbidities – up to 50% are potentially overtreated, as defined by an A1c <7%. National recognition for hypoglycemic safety is evidenced by the creation of a Health and Human Services Federal Interagency Workgroup (with representatives from VA and DoD) to address adverse drug events, including those from hypoglycemic agents. Moreover, the Choosing Wisely initiative to reduce low value care led by the American Board of Internal Medicine Foundation includes an American Geriatric Society recommendation to "not treat most persons over 65 years of age with medications to reduce the A1c<7.5%." For most physicians this involves changes to their current clinical practice. VHA's soon to be announced response to the Choosing Wisely initiative will include hypoglycemic safety as one of its targeted conditions. In addition, the Office of Patient Care Services (PCS) has developed a national action plan to implement a Multifaceted Hypoglycemia Risk Reduction Intervention (MHRRI) to reduce DM overtreatment and risk of hypoglycemia. Our goal is to study the concomitant processes of de-implementation of clinically inappropriately tight glycemic control and implementation of hypoglycemia risk reduction. In so doing, we will inform both VA and the broader federal health community. We propose the following specific aims:

Aim 1. To assess the ***overall*** impact, both intended and unintended, of MHRRI. We will evaluate rates and trends of possible over- and under-treatment and use of specific medications from 2009-2016 among subgroups of high risk patients.

Hypothesis H1.1. There will be a lower rate of (potential) overtreatment post-implementation compared to pre-implementation.
Hypothesis H1.2. There will be no difference in the rate of undertreatment post-implementation compared to pre-implementation.
Hypothesis H1.3. There will be a higher rate of use of high cost medications (with low propensity for hypoglycemia) post-implementation compared to pre-implementation.

Aim 2: To identify factors associated with successful reduction of overtreatment rates and assess factors/differences in implementation processes that explain variation in implementation success. We will assess both macro-(organizational) and micro-(provider) level factors using a mixed methods approach including a focus on positive deviants (facilities with high performance in VISNs of lower performance).

Hypothesis 2. 1a. Facilities with high levels of safety culture (macro-factors) will be associated with greater reduction of overtreatment rates . H2. 1b. Facilities with high commitment to quality (macro-factor) will be associated with greater reduction of overtreatment rates.
Hypothesis 2. 2a. Facilities with high levels of safety culture will be associated with higher exposure intensity of the components of the MHRR Intervention. H2. 2b. Facilities with high commitment to quality will be associated with higher exposure intensity (dose).
Hypothesis 2. 3. Higher exposure intensity (dose) of the MHRR Intervention will be associated with greater reduction of rates of overtreatment.
Research Question 2. 1. Which configurations of the MHRR Intervention components and which factors are associated with greater reduction in overtreatment rates?
RQ 2.2. How does *de-implementation* differ from implementation from a clinician perspective?

Our project will advance implementation science by using an innovative mixed methods multi-paradigm approach to examine potential mechanisms to explain the variation in reduction of rates of overtreatment and to contribute to a better understanding of implementation of multi-component interventions and of de-implementation of established practice.

2. Research Plan

Abbreviations Used in this Application

Multi-faceted Hypoglycemia Risk Reduction Intervention (MHRRI) DM – diabetes mellitus PCP – primary care provider NP – nurse-practitioner Pharm – Pharmacist A1c – glycosylated hemoglobin Clinical Pharmacists/Clinical Pharmacy Specialist (CP/CPS) ADA – American Diabetes Association ACCORD – Action to Control Cardiovascular Risk in Diabetes VADT – VA Diabetes Trial ADVANCE – Action in Diabetes and Vascular Disease: Preterax and Diamicron MR Controlled Evaluation

2.1. Background and Context

The following points, which are integrated into our conceptual framework (Figure 1), establish the background and context for the proposed research and the contribution it will make to health services delivery and research in the VA:

1. *Hypoglycemia is serious, common, and can result from over-intensive glycemic control*
2. *Potential over-intensive glycemic control (overtreatment) of diabetes is common*
3. *Many factors have contributed to the frequency of overtreatment*
4. *Practice change is challenging, and requires a multi-faceted approach,*
5. *De-implementation (practice reversal) may differ from implementation of a new practice.*

1. *Hypoglycemia is serious, common, and can result from intensive glycemic control* - Hypoglycemia is a common accompaniment to diabetes treatment. Risk factors include intensive glycemic control, use of insulin or sulfonylureas, chronic kidney disease, and cognitive impairment.[1] Hypoglycemia rates based on administrative data are underestimates because of undercoding, especially in the ambulatory setting. Rates among high risk Veterans are not known with certainty since VHA does not actively monitor self reported events; physician reporting of adverse events is voluntary; and older patients use Medicare Services and data is not readily available. An analysis using both VHA and CMS data found that the prevalence of combined dementia and cognitive impairment was 13.1% for individuals aged 65 to 74 and 24.2% for those aged 75 and older in 2004.[2] Mean HbA1c levels were 7.0 ± 1.3% for all participants and 6.9 ± 1.3% for those with dementia. The proportion of participants taking insulin was higher in those with dementia or cognitive impairment (30%) than in those with neither condition (24%). Of all participants taking insulin, an outpatient code (or emergency room department/ hospitalization) for hypoglycemia was more common in patients with dementia (26.5%) and cognitive impairment (19.5%) than of those with neither condition (14.4%).[2] However, based on self-report, rates of hypoglycemia requiring third party assistance have been reported to be as high as 14% on oral agents and 59% on insulin in a large HMO.[3]

Hypoglycemia is associated with morbidity, mortality, and healthcare costs. Serious hypoglycemia (defined by the need for 3rd party assistance) is associated with depression and can result in daily debilitating worry, withdrawal from driving, exercise, sex, and going outside of the home. Results from ACCORD, VADT and ADVANCE trials and other studies indicate that hypoglycemia may result in cardiovascular events.[1, 4, 5] While no definitive explanation for the ACCORD mortality results (which contrast with ADVANCE and VADT) it is difficult to completely exclude hypoglycemia or the pursuit of tight control as playing a role. Of note, hypoglycemia is more common at the ends of the spectrum of glycemic control than in the middle.[6]

2. *Potential over-intensive glycemic control (overtreatment) of diabetes is common.* - We have developed a measure of "*potential* over-treatment" operationally defined as: A1c< specific threshold (<7%, <6.5%, or <6%) representing increasingly intensive glycemic control,treatment with insulin and/or sulfonylurea and one of the following (a)>75 years of age, (b) serum creatinine >2 mg/dl or (c) cognitive impairment or dementia. This measure is considerably more

restrictive than one of the recommendations of the *Choosing Wisely Campaign* which is "Avoid using medications to achieve hemoglobin A1c <7.5% in most adults age 65 and older."[7] Nevertheless, our data indicate that up to 50% of patients in VA meeting these criteria are potentially being overtreated (Table 1) Moreover, there is evidence of considerable variation at the VISN and facility levels. For example, variation in rates by VISN and facility for A1c<7% ranged 44.5%-51.3% and 37.3-62.9%, respectively. After excluding A1c <7% in the < 65 year old population and other conditions recommended by VA/DoD Guidelines, ~65% receiving hypoglycemic agents would be considered at high risk for serious hypoglycemia events.

Table 1. Rates of Potential Overtreatement. Study population: patients with diabetes on insulin or sulfonylureas, having A1c in FY09. Advanced DM complications: end-stage renal disease, amputations, advanced retinopathy; Diminished life expectancy: cancer, end-stage hepatic disease; Major neurological disorders: gastroparesis, parkinsons, aphasia, dysphagia, hemiplegia, apraxia, epilepsy, transient ischemic attack; CV disease: myocardial infarction, chronic heart failure

	High risk patients on insulin/sulfonylureas and Age >70y; creat>=2.0mg/dl;	% with <6.0%	% with <6.5%	% with <7.0%
A	Cognitive Impairment/Dementia	10.56	27.4	48.87
B	A+advanced DM complications	10.23	26.52	47.42
C	B+diminished life expectancy	10.46	26.64	47.24
D	C+major neurological disorders	10.48	26.62	47.12
E	D+cardiovascular(CV) diseases	9.87	25.16	44.83
F	E+major depression	9.83	25	44.5
G	F+alcohol/drug abuse	9.93	25.03	44.36

3. Many factors have contributed to the frequency of overtreatment - Our conceptual model(Fig. 1) focuses on the provider; the Theory of Healthcare Professionals' Behavior and Intention, a modification of the Theory of Planned Behavior provides theoretical support for this project.[8-10] Ever since the Diabetes Control and Complications Trial in 1993, the close relationship between glycemic control and DM complications (consequences to patients) has been stressed in the academic literature (knowledge source) with continuing medical education support support from professional societies, advocacy groups, and by pharmaceutical manufacturers (knowledge sources of varying credibility and social influence). Practice guidelines (knowledge source) reflect the interests of these and other stakeholders. General recommendations of target A1c levels have been made by specialty societies and governmental agencies both in the United States and elsewhere.[11, 12] For more than fifteen years the American Diabetes Association (ADA) guidelines have supported a target A1c of <7% for virtually all adults aged 18-75. Similarly, performance measures reflect different stakeholders, but form an important part of the organizational context of healthcare.[11]The National Committee for Quality Assurance's Healthcare Effectiveness Data and Information Set (NCQA-HEDIS®) 's A1c<7 for all patients aged 18-74 years was introduced in 2006 despite the unanimous opposition of the National Quality Improvement Alliance's Technical Advisory Panel.[13] It took the premature termination of ACCORD to modify the <7% measure for persons less than 65 years of age without advanced diabetes complications, cardiovascular or ischemic disease, or dementia. A <8% measure was introduced for all other persons 18-75 years of age; however, no lower level was identified.[13, 14] Unintended consequences of these dichotomous measures have included misplaced focus of clinical efforts including inappropriate intensive glycemic control – overtreatment--and increased frequency of hypoglycemia.[13-16] Changing practice has been difficult. Even in the VA, which never adopted the HEDIS glycemic control measures, we find that the guidelines most commonly cited are those of the ADA and not VA/DoD[17] and VA providers are influenced by the more general marketing efforts of professional societies and pharmaceutical companies. For example, in a survey of clinicians at the Tomah VAMC, October 2011 (where the intervention was developed) 37% agreed or strongly agreed, 10% were neutral and the remainder disagreed with the statement: "I believe that all patients without a terminal

disease should pursue an A1c goal of less than 7." This view is reflected in the lab comments; Fig.2 shows a CPRS A1c report for a patient >75 years at a different facility/ VISN.

Figure 1. Theory of Healthcare Professionals' Behavior and Intention

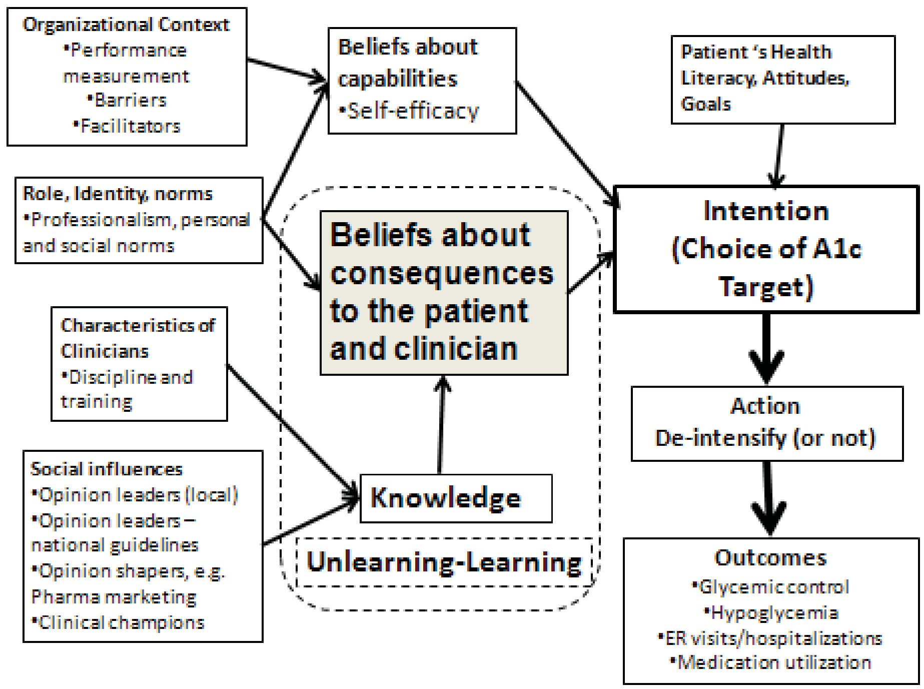

4. Practice change is challenging and requires a multi-faceted approach – Practice change, whether involving guideline implementation or more general quality improvement has proved to be challenging. Multifaceted approaches are critical and at least when it comes to guidelines, allowance for local adaptation is important.[17-23] A systematic review of quality improvement strategies in diabetes (mainly to improve control of intermediate outcomes (A1c, LDL-cholesterol, and blood pressure) found particular support for reminders, decision support, substitution of tasks, e.g., pharmacist-based prescribing, interprofessional collaboration, education outreach, and financial incentives. Mixed effects were observed for educational materials, use of opinion leaders, and audit and feedback.[22] The MHRR intervention is and our simplified implementation model are based on our conceptual model with constructs from the Consolidated Framework for Implementation Research (CFIR).In addition to the need to adopt new practices, the need to reverse poorly supported but established practices is increasingly recognized as evidenced by the American Board of Medicine Choosing Wisely campaign and analogous efforts in the UK. For example, a recent study reviewed of articles in the New

Figure 2. Actual A1c Laboratory Report

```
Report Released Date/Time: XXXX 2013@13:06
Provider: XXXXX
  Specimen: BLOOD.            LOC: XXXXX
    Specimen Collection Date: Jul 30, 2013
      Test name                 Result    units      Ref.   range    Site Code
HEMOGLOBIN A1C                  7.4 H    %         3.0 - 6.1        [541.2]
      Eval: The American Diabetes Association recommends a level of <7%
      Eval: (Diabetes Care, 31[Suppl 1]:S12-54, 2008; PMID: 18165335), and
      Eval: the American Association of Clinical Endocrinologists Task Force
      Eval: recommends a level of <6.5% (Endocr Pract 13[Suppl 1]:s1-68,
      Eval: 2007;PMID: 17613449) as a practice goal for the management of
      Eval: diabetes mellitus.
```

England Journal of Medicine over a 10 year period. Of the 363 articles testing standard of care, 146 (40.2%) reversed that practice.[24] How the process of de-implementation of an established practice, especially one that has been heavily promoted, e.g., intensive glycemic control, differs from the implementation of a new practice is unknown.

5. De-implementation (practice reversal) may differ from implementation of a new practice.

In contrast to the extensive literature on implementation of a new practice or new technology, there are relatively few studies of de-implementation, particularly in health care. In contrast to implementation of a new practice which may involve addition to or substitution for an old practice, de-implementation involves practice reversal, and in addition to learning, requires deliberate "unlearning," and may involve a major change in one's mental model (so-called deep unlearning).[25, 26] Unlearning requires a change in one's knowledge and in the case of a healthcare practice, a change in the beliefs about consequences to the patient and possibly the clinician. Fig. 3 integrates the Becker model of unlearning into the Theory of Healthcare Professionals' Behavior and Intention. These changes may be precipitated by dramatically different scientific findings such as the rapid decrease in estrogen prescribing observed following the release of the Women's Health Initiative questioning the value of estrogen replacement.[27, 28] It may also be a less dramatic process[26, 29, 30]. However, although there are studies of individual unlearning by managers/ administrators therapists treating addictive behavior, our understanding of the process in physicians is very limited.[31, 32] Such knowledge is critical to designing interventions that target the need to abandon outmoded practices. Our study is designed to address gaps in our understanding of the individual unlearning.

Figure 3. Modified Becker Model of Unlearning

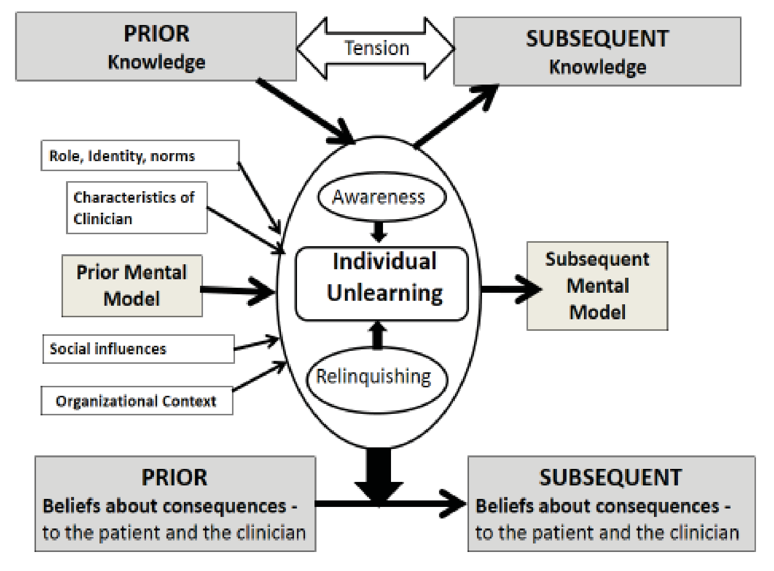

2.2.Significance. The critical issues of overuse of low value practices and medication safety intersect in overtreatment of diabetes (DM). Our project which focuses on the intersection has implications for policy (population health level), clinical practice (individual patient level), and research. Our project is also aligned with and supports efforts of VA Patient Care Services (both Specialty and Primary Care) and Pharmacy Benefits Management related to VA's Choosing Wisely Initiative. VHA provides care to about 5.6 million of the enrolled 8 million veterans; approximately 20-25% of patients have DM. Since 1997 it has issued clinical practice guidelines that have recommended individualized targets and warned against tight control in Veterans with signficant comorbid conditions or decreased life expectancy. As a consequence of landmark clinical trials (ACCORD, ADVANCE, and VADT), increasing attention is now being paid to the risks and the need to individualize glycemic targets and new guidelines have been issued by professional societies and advocacy groups.[33, 34] In contrast, for >15 years the VA/DoD DM Clinical Practice Guidelines included targets stratified by life expectancy and diabetes complications, but their adoption within VA has been hampered, in part, due to strong

marketing of other guidelines by professional societies and pharmaceutical manufacturers, e.g. American Diabetes Association (ADA), that promoted a single A1c<7% target. [35] In a recent review of NHANES data, about half of all adults with diabetes≥20 years of age would have personalized targets ≥7%.[36] Moreover, recent publications on individualization of targets and "systematic" reviews of guidelines have tended to ignore those from VA/DoD.[11, 33-35] Our project will inform policy related to marketing VA/DoD guidelines and interventions to reduce hypoglycemia. The planned initiative is consistent with recent efforts to reduce the use of low value practices; A *Choosing Wisely Campaign* recommendation is "Avoid using medications to achieve hemoglobin A1c <7.5% in most adults age 65 and older; moderate control is generally better."[7] Overtreatment is of low value not only because it lacks benefit, but also because it is potentially harmful.[37] This harm results from hypoglycemia which has implications for both population health and individual health.

As discussed in the Background, hypoglycemia is common, especially in patients treated with insulin and/or sulfonylurea drugs, underestimated and serious. Hypoglycemia is associated with morbidity, mortality, decreased health related quality of life and increased health services utilization (emergency department visits and hospitalization) and healthcare costs.[2,3, 4, 5] Our data indicates that up to~50% of older Veterans with diabetes on hypoglycemic agents are at high risk for hypoglycemia or adverse outcomes because of advanced diabetes complications, serious medical and neurological conditions, decreased life expectancy, substance use and cardiovascular disease. Though there is evidence that take some of these factors into account,[38] there remains a large population at risk. Thus, there are implications for practitioners and their management of many of their patients.

The Institute of Medicine has long proposed that prevention of adverse drug events (ADEs) should be a national patient safety goal. In addition, there is increasing recognition of hypoglycemia reflected in position papers from professional societies. Changing the paradigm for diabetes population health assessment (and management) by emphasizing the importance of potential overtreatment and harms will require a concerted effort. *Underscoring the importance of hypoglycemic safety*, the Office of the Assistant Secretary for Health and Human Services (Office of Disease Prevention and Health Promotion, ODPHP) recently included these agents in an interagency partnership to develop a National Action Plan for ADE Prevention, modeled after the National Action Plan to Prevent Healthcare-Associated Infections. The national action plan was published in the Federal Register on September 3rd for public comment; and will be published in early 2014. Our project will inform this committee's work, which was summarized in a recent presentation to the HHS Health Informatics Technology Policy: Quality Measures Workgroup. We note that the presentation on the ambulatory recommendations for hypoglycemic safety included our HSRD funded work.[37, 39, 40]

In addition, it will provide information about how interventions to decrease hypoglycemia, particularly in the ambulatory setting, can be conducted at the organizational and provider levels. For example, this initiative will test our measure of "potential over-treatment" in real world settings. There are large knowledge gaps are: "de-implementation" and analysis of large scale multi-component interventions. In contrast to implementation, there are very few studies of de-implementation. Although implementation of a new practice that is considered superior may by necessity require de-implementation of an old practice, it is not clear whether or how these processes differ. In particular, intensive glycemic control has been promoted as an essential best practice for many years. Changing the mental models of individual practitioners is more involved than merely implementing something new, e.g., a new drug or surgical procedure. This will likely require different improvement strategies than those used for implementation. Finally, from a manager's perspective, the question is not does some strategy have efficacy under "ideal" conditions, but rather what works when or for whom. Addressing these gaps will be

necessary in order to design more effective interventions that support campaigns such as Choosing Wisely.

The project is mentioned specifically in the DM-QUERI strategic plan:"Goal 2:To work with operations partners to promote evidence-based approaches to improve treatment and reduce complications of diabetes (Diabetes Management/ Secondary and Tertiary Prevention)."DM QUERI relies "on our relationships with PCS to ensure that our projects and activities are aligned with key operational priorities, such as implementation of the diabetes guidelines, current concerns related to hypoglycemia." (p48) Ensuring the safety of insulin and other glycemic control regimens, and avoiding hypoglycemia, particularly in the elderly, is an important focus of the Diabetes QUERI because of the adverse consequences of severe or recurrent hypoglycemia. (p10)There is considerable DM-QUERI involvement. The PI is a member of the Executive Committee and former Co-Clinical Coordinator. J. Lowery is Co-Implementation Coordinator and P. Conlin is Chair of the Executive Committee. In addition, L. Pogach and S. Kirsh, members of the Steering Committee, are co-Clinical Coordinators. DM-QUERI supports this research not only for its contribution to the accomplishment of DM-QUERI Goal 2, but also for its anticipated contribution to implementation science. Aim 2's results will inform the DM-QUERI's efforts to use the CFIR for conducting a synthesis of findings across QUERI implementation studies. Of note, Implementation Science Synthesis was one of the program topics at the 2013 Global Implementation Conference (August 19-21, Washington, DC), where the use of CFIR for implementation science synthesis (a focus of the conference) was presented at the conference and was well-received. Thus, our project will address the needs of implementation researchers, clinicians, managers and policy makers.

2.3 Research Design and Methods

2.3.1. Overview of Study Design (Tables 2, 3 and Fig. 4) We propose a 3-year mixed methods study of implementation of the Multifaceted Hypoglycemia Risk Reduction Intervention that will take place in the context of a natural experiment involving a VA national initiative (Choosing Wisely Campaign and MHRR intervention). Our prospective mixed-methods analysis employing multiple integration approaches to address both formative and summative assessments. relies upon quantitative data for statistical inference validity, and qualitative data will not only provide different elements of richness to the data, it will contribute to interpretative rigor.[41, 42] The modes of mixing include triangulation (assessment of convergence) and sequential use (quantitative methods to identify low and high performers with special attention to positive deviants[43, 44] followed by qualitative assessment).

**Table 2 Strategy**	Goals	Sample
Aim 1 To assess the intervention's *overall* impact	Assess trends of rates of over-treatment, under-treatment, and use newer DM medications (quant.)<br>Identify high and low performers including positive deviants (quant.)	131 VA facilities; FY2009-2016<br>subgroups of high risk patients
Aim 2 To identify factors associated with successful reduction of rates of overtreatment	Assess MHRII implementation (fidelity and intensity) (quant.)<br>Assess association of implementation with organizational factors (safety culture and commitment to quality) (quant.)<br>Identify which configurations of MHRRI components are associated with higher performance (qual. & quant.)<br>Identify factors that distinguish positive deviants (qual. & quant.)	Facility (n=131) surveys<br>Semi-structured interviews (total n= 50) with high and low performers (8 pairs matched for facility complexity)<br>Site visits to positive deviants and sample of other high outliers<br>NOTE: additional details in appendix with instruments.

A baseline assessment will be conducted followed by a 1-yr implementation phase and an 18 month maintenance phase. During the baseline period, we will assess practices as they relate to potential DM overtreatment. Recognizing that the baseline period occurs prior to potential funding dates for the project, the DM-QUERI has committed funds to support work related to that assessment (see support letter). We will then conduct an ongoing fidelity assessment with surveys every three months to determine which components of the intervention are implemented, when they are implemented and whether they are maintained. Overtreatment rates for all VA facilities with >100 patients with diabetes will be assessed at quarterly intervals during the pre-initiative period (2009-1/30/2014) and for the following 2.5 years. Trends of overtreatment rates will be analyzed using a serial cross-sectional design. A similar approach will be used for rates of undertreatment and use of newer medications. Because the intervention will be implemented at different times, we will divide the trend analysis into pre- and post-implementation phases.

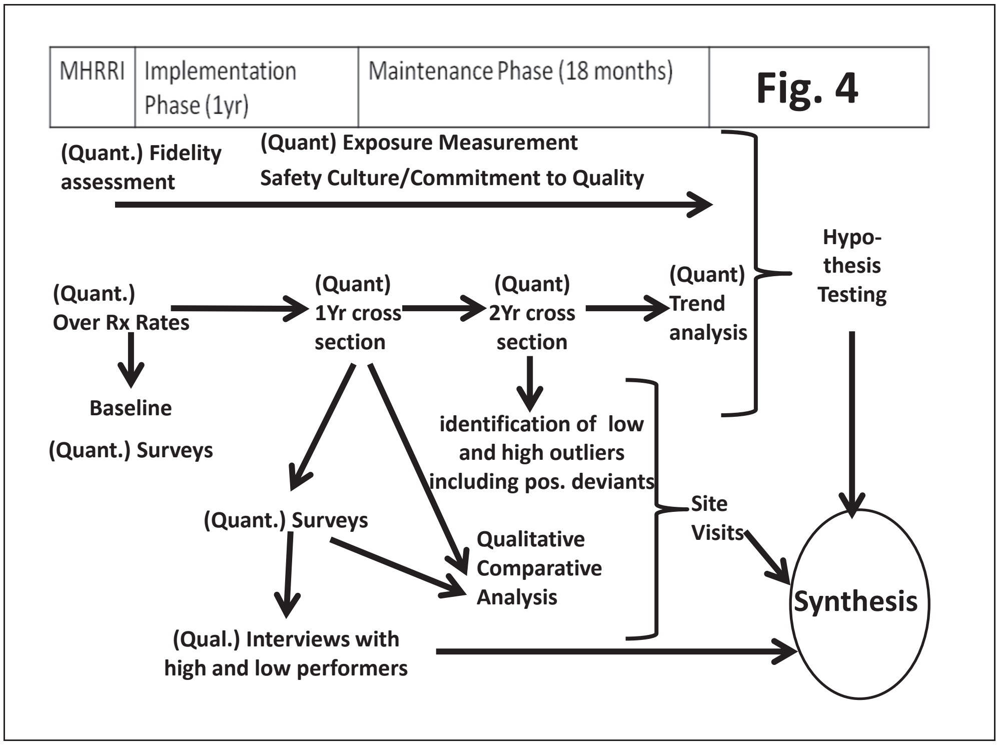

One year following initiative initiation, high and low performers[45] will be identified to provide data for qualitative comparative analysis (QCA, see "Qualitative Analysis below).[46-49] At year two, the findings of the QCA will be assessed in depth via site visits to the high performing sites, with artifact collection, procedural observation, and semi-structured interviews conducted with clinicians including (CPSs, PCPs, and clinic managers), to enhance understanding of contextual factors. Our overall approach is based on the methods developed and used by the HSR&D QUERI/Office of Specialty Care Evaluation Center (D. Aron and M. Ho, co-directors; J. Lowery – leader of Qualitative Analyses team).

We take advantage of a VA national initiative (Choosing Wisely Campaign and MHRR intervention) that will roll out Jan. 30, 2014 and thus constitutes a natural experiment in which "events, interventions or policies which are not under the control of researchers, but which are amenable to research which uses the variation in exposure that they generate to analyse their impact."[50] VISNs will choose one among a limited number of overuse/low value care issues. Thus, randomization is impractical and implementation will vary among facilities both in terms of what is implemented and when it is implemented. Because intervention exposure will not be random, we will use special care in design, reporting and interpretation of evidence in accordance with MRC Guidance and draw causal inferences cautiously.[50] As such, this observational study will be one of assessing effectiveness as contrasted with establishing

causality. Consequently, we will use an *"as treated"* approach. Surveys will be conducted on line with a follow up phone call if no response. Responses related to elements of the intervention will be verified by a second individual at each facility.

**Table 3 Instruments**	**Purpose**	Target	Timing
1. initial survey	existing elements<br>attitudes towards A1c targets<br>potential barriers	Clin. Manager and Pharm D<br>All 131 VA facilities	Pre-initiative
intervention elements portion of initial survey	determine exposure intensity	Same as above	quarterly
2. Knowledge /Attitudes and Self-Efficacy	Clinician characteristics	All PCPs in VISNs 1,10, and 11<br>CPSs at all 131 VA facilities	Pre-initiative<br>And at 1 year
3. MHRRI:<br>Implementation Factors	organizational factors (CFIR)<br>ORCA	All intervention adoption sites, est. ~30; equal number of non-adopter sites matched for complexity.	at 6 months for those who adopt within six months, then at 1 year for the others;<br>and at 2 yrs
4. Master Interview Guide Semi-structured Phone interview	Clinician characteristics; evaluate unlearning process	~45-50 Semi-Structured interviews with participating clinicians (PCPs & CPS) (3 providers) at 8 high & 8 low performing sites (16)	At 1 year post date initiative was adopted at site
5. Master Interview /Site visit Guides; Semi-structured interviews Artifact collection, procedural observation	to evaluate unlearning process	High performing sites & all positive deviants<br>Site visits (~4-8 sites)	At year 2 post date initiative was adopted at facility

2.3.2. Intervention. The Choosing Wisely initiative supported by PBM will be launched Jan 30, 2014 and involves a multifaceted intervention developed in VISN12. The intervention entitled the *Multi-faceted Hypoglycemia Risk Reduction Intervention (MHRRI)* includes the following elements: (1) clinical reminder; (2) CPRS-based decision support; (3) educational outreach (academic detailing); (4) clinical champions; (5) audit and feedback; (6) system redesign for multidisciplinary organization of care; and (7) educational materials. Intervention elements and their targets and mapping to theoretical constructs, are shown in Table 4. Examples of the clinical reminders, audit and feedback reports, decision support and educational materials are shown in the appendix. Clinical pharmacy specialists and other local clinical champions will promote appropriate de-intensification of glycemic control, targeting primary care teams and their patients. There are currently ~1,500 Clinical Pharmacists/ Clinical Pharmacy Specialist (CP/CPS) in 131 medical centers authorized to prescribe in Primary Care and/or Diabetes clinics (PBM – personal communication).[57] They will be tasked by PBM with providing lists to local clinical services of potentially overtreated patients

**Table 4. Intervention Element**	**Intervention Target Mapped to Conceptual Framework**
Clinical champions	Social influence[22]
Educational outreach (academic detailing)	Knowledge, beliefs about consequences and capabilities[22, 51]
Clinical reminder	Awareness[22, 52]
Audit and feedback	Knowledge, beliefs about consequences[22, 53]
Decision support	Knowledge[22, 54]
Multidisciplinary care	Organizational context[22]
Educational materials	Knowledge, beliefs about consequences, patient attitudes[22, 55, 56]

with diabetes at high risk of hypoglycemia. Each of these evidence-based elements has empirical support for its effectiveness in changing practice in diabetes care. In addition, multifaceted interventions tend to be more effective. This combination of elements have been implemented in several VISN12 facilities and, though never tested as such in an RCT, initial results were sufficiently promising for its adoption as an approach in VHA's Choosing Wisely initiative. Some of the results include the evaluation of 2100 PACT assigned patients for hypoglycemia (2,278 evaluations); 17% of patients evaluated (n = 354) reported at least one episode of hypoglycemia. Of these, 9% (n = 32) reported passing out or falling and/or requiring a clinic/ED/hospital visit for management of a hypoglycemia episode and 60% (n = 211) had their diabetes medication therapy relaxed. Rates of relaxation of glycemic control in patients who reported hypoglycemia ranged from 44 to 80%; the lowest rate seen in the most recent intervention site. In addition, there is evidence for shared decision making in choosing more appropriate A1c targets. A1c<7% was the target for those >75 years of age in 36%, half the rate for those ≤75. Intervention implementation will be assessed both quantitatively (see section 2.3.5.1) and qualitatively (see section 2.3.5.3). Of note, the MHRRI meets UK Medical Research Council criteria for a complex intervention: number of interacting components, variability of outcomes, and degree of flexibility/tailoring of the intervention.[50]

2.3.3. Study Sites: Intervention Sites will include all VA sites who implement ≥ 3 components of the intervention within 6 months (2 quarters) of rollout of the Choosing Wisely Initiative. This will allow for VISNs to choose one of four topics on which to work. Previous experience (courtesy of National Program Director for Medicine) suggests that a minimum of 4-6 VISNs will participate, each with an average of 5 facilities. In addition sites in other VISNs may implement this intervention. "On treatment" sites include all sites who implement. (Note: VISN status will be included in our models.)

2.3.4. Study populations/estimated sample size/ power calculations VHA clinic Veteran users with diabetes receiving insulin and/or sulfonylurea treatment in years 2009-2016. The study sample is a dynamic cohort since new members are added as they meet the study criteria. A Veteran is determined to have diabetes if one has VHA service use in the year and has two or more visits with diabetes ICD-9-CM codes (250.XX) from inpatient or outpatient (face-to-face) care over a two year period or has prescriptions for diabetes medication in the year. In FY2009, this population included 463,348 Veterans.

Power analysis: We conducted power calculation based on Type I error rate 0.05 for two-sided tests and used preliminary data. PASS software (version 12.0.2) by NCSS, Inc. was used for the calculation. We have identified > 285,400 (about 25.8% of patient with diabetes) patients across the national 131 VA medical centers in FY2009 with insulin/sulfonylurea who were ≥ 70 years old, or with either diagnosis of dementia or cognitive impairment, or having serum creatinine>1.7 mg/dl. Of them, about 10.5% had last A1c value <6%, 27.1% for less than 6.5%, and 48.4% for less than 7%. In the subgroup of those ≥ 75 years old, or having serum creatinine>2.0mg/dl, or with either diagnosis of dementia or cognitive impairment (n=205,875; 31.5% of those with diabetes), 11.3% had last A1c value <6%, 28.6% less than 6.5%, and 50.0%less than 7%. We have reason to believe the MHRR intervention will have effects on potential overtreatment rates. Work from VISN 12 (where the intervention originated) for a very similar population (age ≥ 75 years old, or creatinine>1.7 mg/dl) shows that the rate of A1c<7% reduced from 34.5% in March 2012, to 32.9% in 9/12, and further to 30.0% in 3/13.

For this analysis, we considered the impact of correlated data due to clustering within facilities and VISNs by applying the concept of effective sample size (ESZ). We calculated ESZ as $(m*K)/(1+\rho*(m-1))$, where m=# of repeated measurements (i.e., number of monthly data per cluster), k=number of clusters (131 medical centers) , and ρ=estimate of Intra-cluster correlation. We assume K=30, a very conservative number of facilities to join the initiative

program. Regarding number of repeated measurement (i.e., number of time points in the time series analysis), we have conducted the power analysis based on the design of 36 months for the pre-implementation and 18 months for the post-implementation (hence unbalanced). The following shows the detectable rate ratios for power =0.8 based on calculated ESZ, and other assumed various pre-implementation rates (e.g., resembling different rates for different A1c thresholds), and an intra-cluster correlation 0.05. The analysis was based on a simple Poisson regression with the independent variable (X) as presence of implementation (1: pre-implementation, 0: post-implementation). The results show that the sample size will provide adequate power to detect a 35%, 43%, and 60% changes in rates if the pre-implementation rate=40%, 25%, and 10%, respectively (i.e., the post implementation rates=26%, 14.25%, and 4%, respectively). We note that increasing intra-cluster correlation and inclusion of covariates (with increasing correlation with X) will reduce the power to detect the same rate ratios (Table 5) if all other parameter values are kept the same. Furthermore, we attempted to conduct power

Table 5 Detectable ratio ratios for comparison of pre- and post- implementation for power =0.8

Effective sample size (ESZ)	m	k	rho	pre-I rate	Post-I rate	rate ratio
444	54	30	0.05	40%	26%	0.65
444	54	30	0.05	25%	14.25%	0.57
444	54	30	0.05	10%	4%	0.4

ESZ (effective sample size) =(m*K)/[1+rho*(m-1)];
m=# of time points = # of repeated measurements; k=# of clusters
Pre-I: Pre-implementation; Post-I: Post-implementation; rho= intra cluster correlation

analysis for the comparison between the implementation versus non-implementation sites. Of the total 131 sites, 30 are estimated to be implementation sites, and the remaining 101 are the non-implementation sites. By making the outcome variable Y for comparison as the difference between the pre- and post-implementation period rates (the "implementation" time for the non-implementation sites being fixed on 2/2014), we simplified the analysis to be 131 observations, one for each facility. Using the same Poisson regression approach, we found that the sample size provides adequate power for only very large effect sizes (i.e., very small rate ratios); the power was 0.70, 0.80, and 0.85, for a ratio of 0.03 if Y=6%, 0.04 if Y=0.10%, and 0.05 if Y=14%.

2.3.5. Dependent and independent variables (quantitative)

2.3.5.1 Dependent variables (Outcome Measures for Summative Evaluation)

Overtreatment: defined as a last A1c value in a time period (described specifically in each hypothesis) smaller than the thresholds of 6.0%, 6.5%, and 7.0%.

Unintended consequence: Undertreatment: defined as a last A1c value in a time period (described specifically in each hypothesis) greater than the threshold of 9.0%.

Unintended consequence: Use of DPP-4 Inhibitors : defined as an active prescription for any amount of time in a time period. Note that the available pharmacotherapy for diabetes has been reviewed.[58] Sulfonylureas and insulin are associated with the highest frequency of hypoglycemia. Metformin has a low incidence of hypoglycemia, but is contraindicated in renal insufficiency, decompensated heart failure, and in patients >80 years of age, although the degree of renal insufficiency that defines contraindication is an evolving issue. Recently, three new classes of drugs have been introduced which have lower incidence of hypoglycemia: (1) incretins, such as glucagon-like peptide-1 (GLP-1) analogues/mimetics, e.g., exenatide; (2) dipeptidyl peptidase-4 (DPP4) inhibitors which decrease the degradation of endogenous incretin, e.g., sitagliptin and vildagliptin; and (3) Sodium Glucose Co-Transporter 2 (SGLT2) inhibitors which reduce renal glucose reabsorption, e.g., canagliflozin.We will assess use of each of these agents. However, because DPP4 inhibitors are administered orally and SGLT2 inhibitors have been released only in 2013, we expect that DPP4 inhibitors will account for most of the non-formulary drug use for diabetes glycemic control.

Intervention Exposure Dose – For our measure of intensity used in the quantitative analyses, we will utilize a variant of intervention fidelity involving dichotomous measures that will account for the fact that elements of the intervention might be implemented at different times.[59] This makes data collection both easier and less costly as well as providing the type of data used in our initial Qualitative Comparative Analysis. Calculation of exposure dose is shown below.

Exposure Dose Score = ∑ (# months element 1 in effect) +(#months element 2 in effect) + …(# months element 7 in effect)   Score will be categorized into quintiles.

Element operational definitions are shown in Table 6. We recognize that how each of the elements themselves are operationalized locally will undoubtedly vary, e.g., perceived influence of the clinical champion will vary by location. Therefore we will also conduct a more detailed qualitative assessment of intervention intensity as part of our analysis of high and low outliers with specific attention to positive deviants. We will accomplish this with interviews and site visits.

**Table 6 Intervention Element**	**Operational Definition (see survey in the appendix) presence/absence of each of the elements (dichotomous)**
Clinical champions	individual appointed with responsibility for implementation
Educational outreach (academic detailing)	Personal education directed to providers
Clinical reminder	CPRS reminder
Audit and feedback	Providers receive reports of their own performance
Decision support	Decision support built into CPRS
Multidisciplinary care	Nurse case managers or Pharm Ds have role in target population
Educational materials	Presence of educational materials to providers about hypoglycemia risk

2.3.5.2 Independent variables (Primary explanatory variable, Process Measures, and Covariates)

Intervention Start Date – quarter in which a minimum of 3 intervention components have been implemented.

Facility Characteristics -- facility complexity (the five- level VA classification) and VISN.

Commitment to Quality at baseline – We will use overall quality of care as a surrogate and use the quality quintile (Gold Star rating) from first qtr FY2014 provided by Strategic Analytics for Improvement and Learning (SAIL) of the Office of Informatics and Analytics. These ratings are calculated quarterly and are a composite of a broad series of measures of all aspects of quality. (see Appendix).

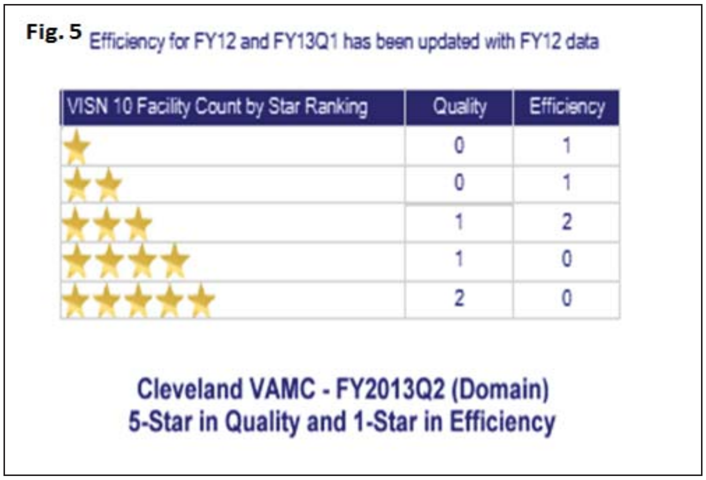

Safety Culture-- We will use results from the National Center For Patient Safety Culture Survey which is conducted every 3-5 years, the latest occurring in 2011. This will serve as the baseline. The follow-up survey will occur after the initiative is underway. We will calculate a score based on results of the domains of general perceptions of safety, organizational learning and perceptions of patient safety at your facility. Scores will be divided into quintiles. We will conduct separate analyses using the relevant questions from the Learner Perception Survey as source of data for safety culture scores (see appendix). Note: this latter analysis will

be limited to medical centers affiliated with universities/training programs. PI is a member Education Work Group, Office of Academic Affiliations, which developed and continues to refine the survey.

Comorbidities: using ICD 9CM codes we will create indicators of advanced complications of diabetes, limited life expectancy, cardiovascular or ischemic vascular disease, and major medical and mental health conditions that would decrease benefit or increase risk of tight control based upon previously published taxonomies. We will create an indicator variable for presence of cognitive impairment or dementia. We will define presence of kidney diseases as having a serum creatinine value>1.7 mg/dL. We will use these variables (and age) to define various subgroups of high risk patients to evaluate overtreatment rates in Aim 1. The primary high risk group will be study population who were ≥ 70 years old, or having cognitive impairment or dementia, or having a kidney disease. We will also use these variables (especially decreased life expectancy or advanced complications of diabetes) to test hypotheses in Aim 2.

Demographic variables including age and sex. As part of our prior work, we have developed algorithms to process multiple entries of patient data over time and from a variety of sources to optimize the demographic information available for research. Sources include many years of VA inpatient and outpatient data with multiple fields and Medicare data. Consequently, we have nearly complete and largely confirmed assignments for most variables, including age and sex. Because of limitations in data concerning race/ethnicity, we will not assess this variable.

2.3.5.3. Identification of high and low outliers and positive deviants. High and low outliers will consist of the top 5% and bottom 5% performers. Positive deviants are defined as high performing facilities, i.e. low over-treatment rates in lower performing environments (VISNs) using a statistical approach recommended by the National Committee of Quality Assurance.[22]

2.3.5.3 Quantitative Data collection

Data sources and Collection: We will obtain VHA data from Corporate Data Warehouse (CDW), a national repository of data, and other VA files. The goal at VA has been to transition most VA data to reside on CDW servers. We will include files containing patient characteristics, outpatient and inpatient encounters, diagnosis and procedure codes, pharmacy/prescription information, and laboratory (A1c and serum creatinine) values from 2004 to 2016. We will use CMS data (when available) which includes Medicare (for outpatient and inpatient encounters, diagnosis and procedure codes, eligibility status) and Medicaid (for additional pharmacy/ prescription information), when they are available, as we have done previously.[40, 60] The data files are listed as follows.

Patient Medical Encounter Data includes file records for all VA inpatient and long-term stays and all outpatient visits, with information on patient characteristics, eligibility, type of care, and multiple codes for diagnoses (ICD-9-CM codes) and procedures (ICD-9-E or CPT4 codes). They are VHA Medical ASA (MEDSAS) datasets.

VA Laboratory Data are recorded at each site for all tests performed and subsets are extracted to national databases. We recognize that not all patients in our study will have results available from laboratory tests. In our prior work, we have found that, based on CPT codes, most VHA patients with diabetes receive the majority of their outpatient laboratory tests in VA clinics, and most patients have regular measures of A1C and creatinine.

Pharmacy Records will be obtained from national VA prescription data maintained by the Pharmacy Benefits Management Strategic Health Group (PBM). The database includes information on the name and dose of the specific medication prescribed, days of medication prescribed, date it is filled, and instructions entered by the doctor on the prescription. The PBM uses a custom software package to extract patient specific medication dispensing data elements from every VA facility on a monthly basis and its verification process assures the

accuracy and completeness of the transmissions. These sources provide data for VA prescriptions only. We recognize that use of non-VA pharmacy services by VA patients became more common since 2007 with the availability of prescriptions through Medicare Part D and increases in the monthly VA co-payment. To address this potential problem, we will include additional measures of prescription drug use in the analysis: (a) Information extracted from physician records on prescriptions the patient received from sources other than the VA that will be part of the PBM data; and (b) Non-VA prescription records from Medicare Part D and Medicaid (if available) and use them together with VA prescriptions in the analysis. We note the information will not be available for the entire study period due to lags.

Mortality data comes from the VA Vital Status File, created as part of the VA-National Death Index Data Merge Project at VIReC, using information from various sources and cross-checked.

Figure 6 Identification of Positive Deviants.

We will construct a 99% confidence interval (CI) for the rate difference between a facility and its corresponding VISN using the formula below where the $VISNrate$ is the average rate in a VISN minus the comparison facility, $facility_{se}$ and $VISN_{se}$ are the standard errors for the $facilityrate$ and $VISNrate$ rate, respectively.

$$(facility\ rate - VISN\ rate) \pm 2.576\sqrt{(facility_{se})^2 + (VISN_{se})^2}$$

Facilities with CIs not containing zero are statistical outliers (potential positive deviants: the facility rate being lower than the VISN rate; potential negative deviants: the facility rate being greater than the VISN rate); the others were non-outliers. Second, we will graph a scatter plot of facilities based on their deltas (=$VISN\ rate - facility\ rate$) and outlier status. We then determine positive deviants from potential positive deviants using proximity to next most extreme outlier and use of box plots.

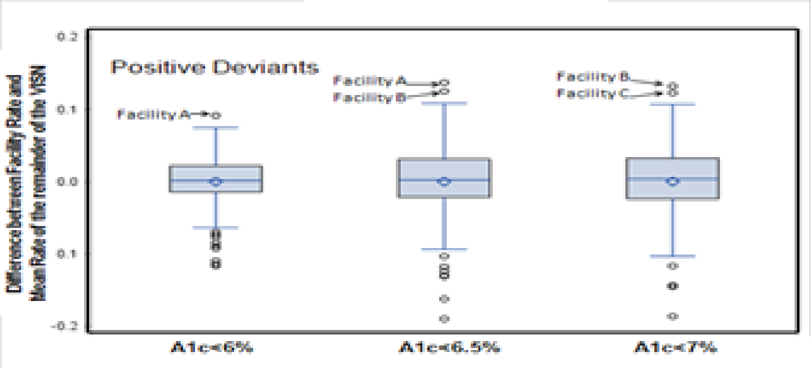

The total of 5 extreme outliers (positive deviants) at different A1c thresholds represent a total of 3 facilities. These facilities have significantly LOWER rates of potential overtreatment than other facilities in their VISN

.

"On treatment" status – This key process measure will be obtained from quarterly surveys of clinical pharmacy specialists at each facility. Participants will be invited and encouraged to take part in an on line survey by the national PBM. Initial survey invitations will be sent before the intervention starts. On line surveys will be built using Inquisite® Surveys will be short enough to be completed within 5 minutes. Each survey will be preceded with a short paragraph explaining the purpose of the survey. Three follow up email reminders will be issued with two to three weeks between each reminder as recommended in the methodological literature. Uncompleted, incomplete, and unclear surveys will be followed up with a phone call. (see Appendix)

2.3.5.4. Quantitative Data analysis

2.3.5.4.1. Statistical Analysis--Overview and general consideration: Our analytic strategy is informed by a model of implementation based on the domains of the Consolidated Framework

for Implementation Research.[61] (Fig. 7) Adopter factors are related to the Theory of Healthcare Professionals' Behavior and Intention (Fig. 1).

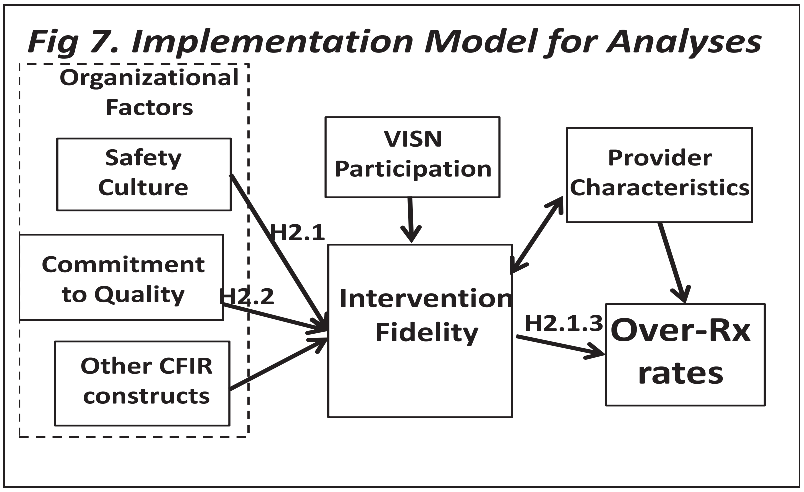

2.3.5.4.2. Statistical Analyses. We will begin all analyses with inspection of frequency distributions, bivariate analyses of means (t-tests and ANOVAs) and rates (Chi-square tests) among subgroups defined by our independent variables. Graphical methods will be used to assist us in preliminary assessment of data to detect information such as direction of trends. We will enter variables into statistical models according to our conceptual framework and hypotheses. Based on our prior experiences with the data sources, we only expect a very small proportion of our study population having missing information on any of the study (independent) variables. As we have done in our prior work, we will assign patients with missing information to be a category of a variable to allow all patients included for the analysis. If the number of patients with missing information is very small (e.g., <100), we may consider removing them from the analysis.

Aim 1. To assess the _overall_ impact, both intended and unintended, of the MHRR Initiative. We will evaluate rates and trends of possible overtreatment and undertreatment and use of specific medications from 2009-2016 among subgroups of high risk patients receiving insulin and/or sulfonylurea treatment defined based on age and comorbidity.

All analyses will be conducted for various subgroups of high risk patients and the following modeling strategy applies to all hypotheses in Aim 1. We will construct and evaluate the data at the level of medical centers, with CBOCs being evaluated separately versus not. Below we use "facility" to refer to the health care unit being analyzed. For each facility, we will calculate the number of patients below A1c thresholds of <6.0%, <6.5%, and <7.0% (i.e., the numerators) of defined high risk patients (i.e., the denominator) and obtain the potential overtreatment rates (=100%*numerator/denominator) on a monthly (alternatively, quarterly) basis. Undertreatment rate will be defined using A1c >9.0% and will be derived in a similar fashion as the potential overtreatment rates. We propose to use the approach of interrupted time series and construct segmented Poisson regression models to analyze the data.[62] We will use monthly data of number of patients qualifying for potential overtreatment (H1.1), undertreatment (H1.2), and receiving DPP4 inhibitor use (H1.3) as the dependent variables. Number of days in a month will be included in the model as an independent variable. A time (period) indicator (1/0) will be created with hypothesis specific definition to allow evaluation of rates from two segments in the time series of data. For H1.1 and H1.3, value 1 in the time indicator is the post-implementation period, and value 0 is for the pre-implementation period. For H1.2, value 1 from the time indicator refers to years 2009-2013 and 0 refers to years 2014-2016. We will evaluate the empirical trend of the rates by graphing time series of monthly rates from 2009 to 2016 and include a time variable (with 10/2008 as the starting month) and appropriate polynomials of time to account for underlying trend of the rates over the years. This will allow us to adjust for secular trends in that rates simply change over time for reasons not related to the intervention. The interaction terms of the time indicator and the time variable(s) will be added to allow evaluation of changes in time trends (e.g., slopes) in two time periods. We will account for temporal

fluctuations in A1c values by including trigonometric terms, sine and cosines, to describe seasonal patterns.[60] Additional selected variables (e.g., VISN indicators, patient characteristics, etc.) that may be related to variation in rates and are different in groups of comparisons will be included in the models as well. Finally, facility-specific monthly number of populations at risk for the outcomes (i.e., patients alive at the beginning of the month) will be included as an offset variable, as typically done for modeling of rates using Poisson regressions. If we find the initiative is statistically effective in reducing the rates of interest, we will evaluate the impact of individual elements in reducing rates by entering separate indicators for their presence.

Our modeling strategy permits adjustment for important covariates that change over time by including them in the models as time-varying variables; it helps to reduce bias in the comparison of pre- and post-intervention outcomes in the presence of time-dependent variables. Another strength of the study is the participation of various VISNs (likely from different regions, with different facility cultures/characteristics and patient composition). It provides us an opportunity to evaluate robust findings of the initiative in various settings rather than in just one or very few settings. In other words, we can assess and compare the impact of initiative in different VISN/facility environments through assessment of the interaction terms between the time indicator and VISN and/or between the time indicator and facility. In the analysis, we plan to assess the effectiveness of the initiative based on individual facility's start date rather than a fixed date (e.g., 01/30/2014, the planned start date of the initiative). Our modeling strategy provides flexibility to allow for differential starting dates among facilities (even within the same VISN). However, we will separately fix the intervals of pre- and post-initiative to be the same across facilities. The actual starting date is defined as the earliest date of those elements implemented in a facility. It is no earlier than 01/30/2014 by design, and we will include those starting the initiative no later than 1/30/15 for analyses of intervention adopters so the post-initiative period will be at least 18 months long. In determining the length of the pre-initiative, it is important to have a longer baseline (pre-initiative) period to allow observation of a more stationary series (e.g., fluctuation of rates within a certain range) in evaluating the effect of an initiative/intervention because an intervention is easier to detect in a stationary series [63] (We have considered assessing data at least three years (36 months) prior to the initiative. This will give us ~ 50 time points (for monthly) for the combined pre- and post-initiative periods and 50 is a good round number in addressing the concern related to data being stationary or not [63] We will use the above mentioned graphs of rates of interest over the study time period: 2009 to 2016 to guide us and assess if an even longer pre-initiative trend (i.e., including data beyond the 36 months) may change the results of our evaluation of the effectiveness of the initiative.

In comparing between the adopter and the non-adopter group, indicator of presence of the initiative (1: yes' 0: no) and its interaction with the time indicator (experiment X time indicator) as well as its three way interaction (experiment X time indicator X time variable) will be entered into the statistical models. A negative sign for both the time indicator and the two-way interaction term will evident that the rate decline is greater in the adopter group. With regards to adjusting for covariates that may distribute differently in the two comparison groups and may be related to the rates of interest (i.e., confounders), other than the above commonly used regression approach, we have considered conducting the comparisons within subgroups/strata defined based on levels of facility complexity. We will assess if we have adequate number of facilities in each subgroup to allow meaningful comparison before the actual data analysis. We have also considered two commonly mentioned methods (for person level of analysis) are propensity score analysis and instrumental variables (IV) approach. The former adjusts for differences in observed covariates and the latter can handle even the differences in unobserved covariates. However, the IV approach relies on identification of an appropriate IV to have valid results. We have not been able to identify any good IV candidate for this analysis; therefore, we do not plan to use this approach for the analysis. Of the two alternative methods, the propensity score

analysis is used more commonly. It involves an initial step of deriving a propensity score for the treatment (or in the experiment group) for each subject, which is then followed by uses of the propensity score in the main analysis (either as a covariate or for matching). We will consider using an application of the propensity score approach based on a developed method for time series data [64]

The study outcomes are inherently correlated because facility-specific monthly data are clustered within a facility and facilities are clustered within a VISN. In the above Poisson regression models, we will include facilities as random effects and use random-effects models (also termed as mixed effects models, hierarchical models, or multi-level models) to accommodate correlation of the time series data (e.g., specifying the correlation structure among the time series data within a facility as first order autoregression). VISN will be treated as a fixed effect and VISN indicators will be entered into the model. This random-effect modeling approach is commonly used in longitudinal analysis of repeated measurements over time. It helps address the issue of underestimating the variance of the regression coefficients (hence invalid inference) based on conventional regression models that rely on an assumption of independence among observations.[65]

Aim 2: To identify factors associated with successful reduction of rates of overtreatment and assess factors/differences in implementation processes that help explain variation in implementation success. We will assess both macro-(organizational) and micro-(provider) level factors using a mixed methods approach including a focus on positive deviants (facilities with high performance in VISNs of lower performance).

Continuing from the random-effects segmented Poisson regressions in Aim1, we will test the following hypotheses by adding in the models each individual independent variables of interest and its interaction term with time indicator (1/0). Specifically, for H2.1 and H2.3, the outcome variable is monthly rates of overtreament. The factors of interest, safety culture (H2.1a and H2.2a), facility commitment (i.e., culture of quality at baseline; H2.1b and H2.2b), and exposure intensity of MHRR initiative (H2.3) will be separately evaluated (along with its interaction with time indicator). To test H2.2, we will evaluate the relationship of exposure intensity of MHRR initiative with safety culture (H2.2a) and commitment (H2.2b) using statistics such as Gamma, Kendall's tau and variants, because they are all ordinal variables with limited number of categories in each variable. Gamma statistic is calculated based on number of concordant (in agreement) and discordant (in disagreement) pairs of the observations (i.e., facilities here) while ignoring ties and is suitable when there are large number of ties. Kendall's tau and variants correct for ties. A +1 and -1 for these statistics indicate the presence of a perfect positive and negative correlation between the two variables. A value close to zero indicates an independent relationship between the variables.

<u>Research Question 2. 1</u>.Which configurations of the MHRR intervention components and which factors are associated with greater reduction in overtreatment rates?

Data collection and analysis for this research question will use a mixed methods approach. Semi-structured interviews will be conducted with key informants from high and low performing sites approximately one year after the initiative has been implemented at each site. Interview data will be analyzed to identify the intervention components and organizational factors that distinguish between low and high performing sites. Surveys of a larger sample of clinical leaders and primary care providers will also be administered at approximately the same time to obtain data on provider characteristics, as well as data on interventional components and organizational factors. The latter data from the surveys will be compared with the interview data to help validate our analyses of the interview data. In turn, the interview data, which will contain much more in depth information on barriers and facilitators to implementation of MHRRI, will help us better understand the survey results. Additional detail is provided below.

Additional Variables and Data Collection

Provider Factors: Provider surveys will be administered approximately one year following implementation of the initiative to clinical leaders and primary care providers at each site. Provider characteristics include clinician type (MD, DO, NP, PA); years in practice; gender. We will assess clinicians' knowledge using CME questions related to results of recent clinical trials, e.g., ACCORD, ADVANCE, and VADT. We will assess attitudes towards glycemic targets with a Likert scale-based instrument that has been used in VISN 12. (see appendix) We will assess efficacy using the Provider Abilities subscale from the Midwest Clinicians' Network Barriers to Diabetes Care Survey questionnaire. [66] The questions are rated on an 11-point Likert-type scale assessing degree of confidence and tap confidence in ability related to medical processes, communicatiing with patients. We have added items to assess efficacy/confidence in ability to utilize shared decision making in general and with respect to setting targets for glycemic control as well as disease-specific management issues. We modeled these items and format after Glazier et al's scale assessing efficacy/confidence in the primary care management of musculoskeletal disorders. [67]

Organizational Factors:-We will use the Consolidated Framework for Implementation Research (CFIR) framework (See Appendix) to conduct a baseline and post-implementation (interpretive) process evaluation to determine those organizational factors associated with implementation success.[61] Selected constructs (from CFIR's 39 constructs) will be used to design the interview guide. We will identify a subset of constructs most relevant to this initiative based on surveys of the participating clinical pharmacy specialists, administered prior to the start of the initiative. (Since this grant will not be funded by the time implementation begins in January 30, 2014, we will conduct these surveys using other DM QUERI resources.) The survey will consist of questions assessing the potential importance of each CFIR construct (see Appendix). This survey has been used in the evaluation of four initiatives (E-Consults, SCAN-ECHO, Specialty Care Neighborhood, and Mini-Residencies) so far in the Specialty Care Evaluation project.

A semi-structured interview guide will then be developed consisting of questions designed to obtain input on the constructs identified as most important from these baseline surveys. As part of the Specialty Care Evaluation, we have developed multiple interview guides addressing most of the CFIR constructs. These questions have been used successfully thus far for three different Specialty Care initiatives; so, it will be relatively simple to pull the relevant questions from these previously developed guides and tailor them for this study. The interview guide from the Specialty Care Neighborhood is provided in the Appendix as an example. Potential respondents (the clinical pharmacy specialist, director of primary care, and two primary care providers) will be contacted via an email invitation followed by a phone call. All interviews will be conducted by two research team members, with one conducting the interview (audio-taped) and one taking field notes (written or typed). We will use a rapid analytic process (see below) to obtain findings in a timely manner.

In addition to the semi-structured interviews, we will administer surveys to a larger sample of providers (i.e., all primary care providers) at approximately one year following implementation of the initiative, at all sites that participated in implementation. This is the same survey that will be used to obtain data on provider characteristics (described above), and will also include questions regarding organization characteristics that may have been barriers or facilitators to successful implementation of the initiative. These questions will include questions from the Organizational Readiness to Change (ORCA) instrument,[68] as well as questions assessing the impact of CFIR constructs.

Qualitative Analysis. Typical to qualitative methodologies, data analysis will be ongoing, throughout the data collection period.[41, 42] This will allow us to define new themes and categories as they emerge from the interviews; although our focus will be on coding the data

according to the pre-specified themes (constructs) from our fidelity checklist and the CFIR. Most of the interview guide questions will define specific fidelity and CFIR constructs that will allow the researchers to efficiently collect and evaluate the appropriate data. Following each interview, the research team reviews interview notes. Each member of the team independently assigns the appropriate CFIR code(s) to each response. Although each question focuses on a specific construct, the questions are largely open-ended, allowing the respondent to expand on the topic, thus potentially discussing additional constructs and requiring additional codes. The team then gets together to reach consensus on the codes for all responses for a given interview. Following the coding of the interviews according to the fidelity and CFIR constructs, each team member independently reviews the responses associated with each construct and assigns an ordinal rating to the construct (-2, -1, 0, +1,+2), which reflects the respondent's perception of the influence of the construct in the organization (positive or negative) and the magnitude or strength of that perception. For example, under the construct of "relative priority", if there are a number of other high priority clinical programs competing for resources/attention, the construct would be assigned a -1 or -2 rating (depending on how detrimental the respondent feels these competing programs are relative to the MHRRI). In contrast, if the MHRRI has a high priority compared to other programs, then a +1 or +2 would be assigned. Following assignment of these ratings, the two-member team gets together to reach consensus on the ratings for each construct, for each respondent. Once this process is completed for all respondents at a given site, the team meets to assign an overall rating for each construct at the site level, considering the ratings across respondents and each respondent's role in and knowledge of the MHRRI. Thus, the responses for some respondents might be weighted more heavily than others in determining an overall site rating. This coding and rating process [69]has been used successfully in HSR&D/Office of Specialty Care (OSC) evaluation. The process provides a more systematic means of linking constructs to implementation success (see below) than simply organizing qualitative data according to themes. As we have done in coding interview data for the HSR&D/OSC evaluation, analysts will be encouraged to also identify emergent codes or themes not included in the CFIR. Often these can be assigned to CFIR codes after further consideration; however, sometimes new themes emerge, and we have used these to refine the CFIR. Data will be analyzed initially using a simple matrix format to examine potential correlations between each construct and program performance (low vs. high). Strong correlations will then form the basis of specific recommendations for subsequent program improvements and dissemination. Table 7 provides an example of what the matrix might look like for analyzing the post-implementation data. Constructs A and B do not appear to be correlated to program performance, in that there is no orderly progression from low to high magnitude of the construct. In contrast, constructs C and D do show a correlation, suggesting that these constructs might be important factors affecting successful implementation of the program. Thus, these findings can be used to develop recommendations for future efforts to disseminate the program, depending the factors and how they are manifested in the individual sites.

Table 7: Example Findings from Qualitative Analyses of CFIR Interviews

	**Site (Performance)**			
**Construct**	**1 (Low)**	**2 (Low)**	**3 (Hi)**	**4 (Hi)**
**A**	-2	+1	0	+2
**B**	+2	+1	0	+2
**C**	-1	-2	0	+2
**D**	-2	0	+1	+1

We have previously employed rapid analytic methodology in an implementation study of diabetes registries, and this type of ordinal rating of qualitative data is increasingly being used in studies of organizational change. This more rapid approach will enable more timely feedback to the sponsors of the Choosing Wisely Initiative to inform program modification. However, audio

recordings will be available for later transcription and validation of the findings of rapid coding. The coded interview data along with the quantitative data on implementation intensity will also be analyzed using Qualitative Comparative Analysis (QCA), which is a case-oriented, comparative analytic method based on set-theoretic relationships (as opposed to statistical or correlational relationships) between explanatory factors and an outcome. It uses formal logic for systematic cross-case comparisons across a small to intermediate number of cases. It is useful for studying causal complexity—when different combinations of factors can lead to the same outcome, when some factors may only be causal in the presence of other factors, and when the absence of the outcome has a different causal pathway than simply the absence of factors associated with the outcome (i.e., causal asymmetry). For example, the factors associated with being a low performing facility may not simply be the absence of factors associated with being a positive deviant.

Cases are selected for use in a QCA based on the outcome, with a goal of maximizing heterogeneity in outcome. Thus, QCA will be useful method for explaining positive deviants, as it will include both high performing AND low performing cases in the analysis.[47, 70, 70] In QCA, independent variables are called “condition sets” and the dependent variable is the “outcome set.” Sets can be thought of as data containers or boundaries that define zones of inclusion and exclusion. Researchers establish the zones through a process called calibration and assign cases a set membership score within each condition set and the outcome set according to whether the case fits within the boundaries of the set as defined. Figure 8 shows how different intervention components or contextual factors, or any other explanatory variables can be operationalized as condition sets. In QCA, the set, subset, and superset relationships between the condition sets, combinations of conditions sets and the outcome set are analyzed. Set relationships directly correspond to verbal statements of necessity and sufficiency; a necessary condition set is one that exhibits a superset relationship to the outcome set, while a sufficient condition set is one that exhibits a subset relationship to the outcome set.

For this analysis, we will use our conceptual model to define approximately five to six conditions for use in the QCA. The case to condition ratio in a QCA is generally at least 3 to 4 cases per condition included.[46] These conditions will likely be use of various intervention components, fidelity of implementation, facility contextual information, and patient population context. The process of calibration is iterative with QCA; thus it is not necessary to completely finalize condition specification and calibration before beginning the analysis. We will determine the most suitable calibration scheme (crisp set or fuzzy set) based on the nature of the condition. In “crisp set”, conditions are calibrated dichotomously (e.g., condition “A” is present (1)/condition “A” is absent (0), condition “B” is high (1)/condition “B” is low (0)). With fuzzy set calibration, cases can be assigned condition set membership scores of any number between 0 and 1. Condition set membership scores are assigned based on the quantitative degree to which a case is “in” or “out” of the condition set, with 0.5 as the threshold characterizing the qualitative difference in set membership (> 0.5 is more “in” than “out”, and < 0.5 is more “out” than “in”). Fuzzy-set calibration allows analysts a more nuanced categorization, reflecting the reality of the real world where conditions are not simply present or absent. The set membership scores assigned for each condition and the outcome for each case are placed into a data matrix called a “truth table”, which is the *sine qua non* analytic device in QCA. The truth table has 2^k rows, where k is the number of condition sets in the study, with each row representing a theoretically possible configuration of condition sets. Each empiric case within the study is sorted into one (and only one) of these rows; however, cases with the same configuration of condition sets will occupy the same truth table row. The truth table is analyzed to identify subset, and superset relationships between condition sets and the outcome set. Boolean algebra based on the logical operators “AND,” “OR,” and “NOT” is used to logically minimize the sufficient conditions (or combinations of conditions) identified. Findings derived from the truth table analysis are expressed in terms of

necessary and sufficient conditions using narrative text, solution formulas, Venn diagrams, and for some types of fuzzy-set QCA analyses, XY plots. Sometimes these are referred to as “causal recipes”. Parameters of fit are calculated to assess the degree to which the causal recipes derived from the empiric data deviate from perfect set relationships (i.e., consistency) and explain the most number of cases in the outcome set (i.e., coverage). In addition to conducting analyses of necessity and sufficiency for the outcome of being a high outlier, we will conduct similar analysis with the outcome of being a low performing facility. This is required because the causal recipe to failure can be as useful within a learning organization as that of success. The difference between our two analytic approaches (matrix correlation analysis vs. QCA) is that QCA will identify combinations of necessary and sufficient factors through an analysis of set relationships that, when present, “guarantee” implementation success. This contrasts with the matrix correlation analysis approach, which seeks to identify those constructs that statistically correlate with high performance—i.e., indicate a higher probability of success than when these constructs are absent or manifested negatively. The benefit of QCA is that it provides specific recipes for implementation success; where matrix correlation analysis can only suggest which individual constructs are independently associated with success. We believe both approaches are very useful. The combination of “successful” constructs from QCA implies a rich and complex path to success or failure – i.e., different combinations of constructs may equally result in success or failure. It is possible to obtain results showing that no two high performing sites exhibit the same pattern of constructs sufficient for achieving high performance. Thus, it might be difficult to come up with specific recommendations for potential areas of focus for widespread dissemination. In matrix correlation analysis, recommendations focus on those individual constructs that appear to improve one’s chances of achieving high performance. Once the significant constructs and combinations of constructs have been identified from our analyses (i.e., those constructs that are associated with high performance), we will go back to the summary memos from our analyses to obtain additional information on the specific practices at sites in which these constructs were positively manifested. These “best practices” will then become the basis of our specific recommendations for dissemination of MHRRI. Dr. L. Kahwati will provide expert consultation on the use of this method. She has used this approach previously in determining best practices in VHA’s MOVE! Weight Management Program for Veterans[70] and is PI on a current Agency for Healthcare Research and Quality-funded methods grant to assess the feasibility of using QCA to explain heterogeneity within systematic reviews of complex interventions.

Fig. 8. Qualitative Comparative Analysis

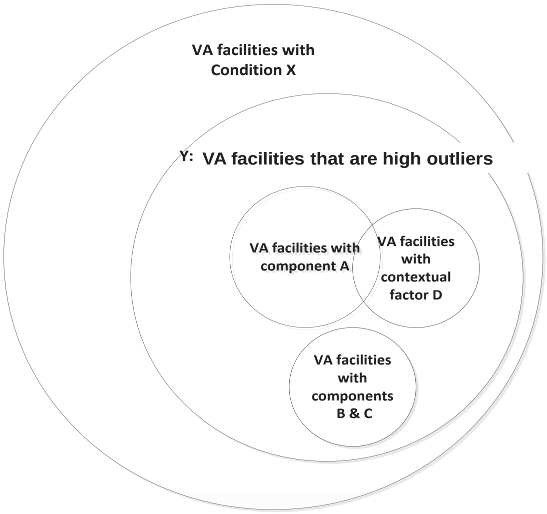

Necessary conditions are supersets of an outcome set.

- Condition X is a superset of the outcome set Y.
- X is necessary for Y. However, X does not guarantee the outcome Y. A case can have X, but still be outside of the set Y.

Sufficient conditions are subsets of an outcome set.

- Conditions A, D, and the combination of B and C are subsets of the outcome set Y.
- Any one (A, or D, or B & C) of the sufficient conditions is linked to the outcome Y. All cases with any one of these conditions are within the set Y.

Note: A, B, C, D and X can represent different features, contextual conditions, or characteristics of VA facilities.

Mixed Methods Analysis[42, 71] – Since there is currently no gold standard measure of organizational barriers/context for implementation success, we will triangulate data from three different sources to help us understand the different measures and help us in our effort to develop valid measures of the CFIR constructs. The ratings of the CFIR constructs (-2, -1, 0, +1, +2) from the interview data, which will be obtained from a subsample of participants, will be compared to the survey responses from the larger sample of respondents. The survey responses, in turn, will consist of data from two different scales: the CFIR questions and the ORCA questions. We will examine whether there is a correlation between the ratings across these three sources. While qualitative data obtained from semi-structured interviews can significantly improve our understanding of how particular organizational factors influence implementation success, the limitations of these interviews is that they can usually be conducted with a much more limited number of stakeholders than can be reached via surveys. With a limited number of interviews, therefore, we cannot be sure that the people interviewed are representative of other key participants in the initiative. In turn, we will gain a better of understanding of whether the ORCA and CFIR questions are capturing the intended meaning of the constructs. Once the analyses have been completed and the key constructs identified, we will conduct site visits to the high performing sites. The primary purpose of the site visits will be to obtain detailed information on site-level manifestation of the constructs associated with implementation success, for purposes of developing an implementation toolkit that can be used by other sites. Interviews will be conducted with the clinical pharmacy specialists, primary care providers, and clinical managers. Clinics will be visited and artifacts collected, e.g., education materials, screen shots of CPRS (using Test Patients) illustrating decision support, clinical reminders, and lab comments on A1c.

<u>Research Question 2.2</u> How does deimplementation differ from implementation from a clinician perspective? In addition to surveys, we will conduct semi-structured interviews with clinicians from high and low outliers using the modified Becker model of unlearning. Instruments will be refined during the first 6 months of the project.

2a.3. Contingencies, Limitations, and other Considerations. There are several factors with significant potential impact on the project including timing and completeness of data, particularly to assess MHRRI implementation. First, the potential funding period doesn't align with the initiative roll-out, i.e., if funded. Therefore, we have obtained DM-QUERI support to collect baseline data and we plan to start this collection as soon as IRB approval for that portion is obtained. We don't anticipate major concerns about collecting data on what aspects of the intervention may already be in place. The initiative may be delayed (although according to the National Program Director, it is on schedule). Therefore, we have built in a one-year period for implementation which should accommodate a delay of up to six months. However, if necessary, we will request an extension of the project by the necessary amount of time. CMS data is likely to be delayed, but VA data alone should suffice for the key variables. As a natural experiment, we cannot prediction which facilities will be adopters. Therefore for our intitial PCP survey, we will use all PCPs from three VISNs, those of three of the investigators. These are very heterogenous and will likely have adopters and non-adopters. Sample size may be jeopardized if an insufficient number of facilities choose this (as opposed to another) initiative. However, we are aware of three VISNs who have expressed interest in this project, especially because of its potential to reduce the costs associated with hypoglycemia in the elderly, e.g., hip fracture from falls. Second, the project is contingent upon obtaining data about intervention implementation and fidelity. We are taking several approaches to this concern: (1) by using an extremely brief survey, the respondent burden will be minimal; (2) PBM is strongly committed to working with clinical pharmacy specialists in the field to make sure that the data are collected for their own quality improvement uses; (3) there are multiple potential sources of data at facility and VISN levels and we will follow up with as many as need be; and (4) members of the research team,

steering committee, and DM-QUERI have extensive social networks which will enable identification of individuals who can facilitate the process) For surveys of PCPs, if response rate is too low, we will focus on networks where we have the most extensive social networks. The study design is quasi-experimental using a natural experiment so that adopters and non-adopters may differ significantly at baseline. Our pre-post analysis for each facility based upon its implementation date will help to ameliorate this concern. We will also explore other methods to adjust for baseline differences, e.g., propensity models for time series data. We are studying a single disorder – diabetes - and findings may not generalize to other conditions. Nevertheless, DM is not only prevalent, but has been a good model for issues related to chronic disease. Finally, a key stakeholder group is not included in this proposal – patients with diabetes and their families. Anecdotally, it appears that some patients, who like clinicians, have been subjected to the marketing campaigns of the ADA, National Diabetes Education Project, and pharmaceutical companies, are reluctant to relax their levels of glycemic control. In addition, clinician-patient interactions affect the choice of targets. Our omitting this important group was done for two main reasons. First, it would expand the scope of the proposed project considerably and could not be accomplished within the budget. Second, our experience with IRBs has been characterized by major delays whenever patients are surveyed or interviewed. Therefore, we plan to submit a separate proposal specifically addressing patients and clinician-patient interactions. Similarly, we considered using the RE-AIM framework, a commonly used population health impact framework. In addition to Effectiveness, Implementation, and Maintenance which our proposal addresses, RE-AIM also addresses Reach (e.g., participation rate among eligible individuals (% of patients on insulin and or sulfonylurea seen in sites which implement the MHRRI); and Adoption (participation rate among possible settings and the representativeness of settings participating). However, although informative, the additional data collection required would expand the scope well beyond the budget and/or introduce a degree of respondent burden that could jeopardize our ability to obtain complete data on implementation. Although modest, the initial results of the intervention in VISN12 were sufficient for its adoption as an approach in VHA's Choosing Wisely initiative.

We have chosen to use qualitative comparative analysis (QCA) even though its application in health care has been limited. Our choice of (QCA) was based on the fact that VHA's Choosing Wisely initiative is a complex intervention nested within a complex and open health care and social systems, and as such issues of causal attribution are more problematic. In addition, some components may only be causal when present in association with other components (conjunctural causality). Similar outcomes may result from different causal pathways (equi-finality). In addition, the number of facilities is too large for intensive case studies of all of them. Yet, the number of facilities in any particular subset of configurations of intervention components may be too small for a regression approach. QCA addresses the middle ground and does so effectively.[48, 51] Finally, we recognize that the Choosing Wisely initiative is occurring in a changing health care environment where something completely unexpected is likely to happen. We will be attentive to developments that potentially affect the initiative and be prepared to modify our procedures to take them into account.[72]

3.0. Dissemination of Findings and Project Management

3.1. Dissemination Plan -- The dissemination plan is illustrated in Table 6 (next page). In addition to presentations at national meetings (e.g., VA HSR&D Service meetings, Association for Health Services Research) and submission of results to peer-reviewed journals, there are other means for dissemination of the findings. The PI serves on network committees in VISN10, including the Chronic Care and Health Systems Design Committees. Results, particularly those with policy implications will be shared with these committees along with the Executive Leadership Council (which includes all the Service Line Directors and Chiefs of Staff), the network Chief Medical Officer, and the Network Director. Moreover, our steering/advisory committee has representatives from VA HSR&D, Patient Care Services (PCS) including PBM,

and NCPS as well as a Network Director and CMO. In addition to their advisory role, these committee members will provide input for effectively and efficiently disseminating relevant findings. In addition to their links beyond the VA, we specifically note that Dr. Leonard Pogach is PCS VA National Program Director, Diabetes. He is also a member of the DM Interagency Coordinating Committee consisting of NIDDK, CDC, DOD, AHRQ, VA, Indian Health System, and other federal agencies. He is also Chair of the federal interagency work group to address adverse drug events, including those from hypoglycemic agents.

**Table 8. End Users**	**Needs**	**Means**
Researchers	Scientific findings	Publications, scientific meetings
Clinical Operations - Service/Section Chiefs	Scientific and Operations-relevant findings	Presentations at Field Advisory Committee Meetings.
Managers - CMOs, QMOs, VISN Directors and facility equivalents	Operations-relevant findings.	Executive briefs. Drs. Gelman, CMO, VISN10 and Dr. Murawsky, Director, VISN12 will assist.
Policy – Offices of Primary Care, Specialty Care, PBM, Academic Affliliations	Operations-relevant findings.	Executive briefs. Representatives of these offices serving on the Steering Committee
Choosing Wisely Initiative/ Interagency Task Force	Operations-relevant findings.	Executive Briefs. Representatives of these initiatives serving on the Steering Committee
Broader professional diabetes community – ADA, AACE, NIDDK, Endo. Society, , PCORI	Primarily, scientific findings	Meeting abstracts, publications, social networking with other members of these societies and colleagues at NIH and PCORI

3.2. Project Management Plan -- The general organizational structure of the project is shown in Table 8. The Steering/Advisory Committee will convene with the PI quarterly for the first year and then semi-annually by teleconference. We will also take an opportunistic approach and meet when there are a significant number of members present, e.g., at QUERI, VA HSR&D or Academy Health annual meetings as well at other national meetings for VA. Individuals will also be contacted and provide guidance as needed between scheduled meetings. The external advisory committee members were selected for their expertise in research, healthcare delivery, and training. Specifically, Dr. Kerr, Director of QUERI-DM, has vast experience regarding all aspects of conducting and managing research pertaining to patients with DM. Dr. Pogach is an established health services researcher and Chair of the VA Clinical Practice Diabetes Guidelines. The Internal Steering Committee will meet every other week initially and then monthly (or more often as necessary) and will be responsible for coordinating the various sub-parts of the project. The Intervention Team Task Force will be responsible for addressing any issues or concerns that arise in the implementation of the interventions and will meet monthly. The Data Management and Analysis Team will oversee all aspects related to data collection and management (e.g., construction of the necessary databases, coordinate data downloading and merging, oversee the preliminary analyses). It will meet weekly or monthly as project needs evolve. The Project Team will oversee the daily operations of the project and will review time

The Data Management and Analysis Team will oversee all aspects related to data collection and management (e.g., construction of the necessary databases, coordinate data downloading and merging, oversee the preliminary analyses). It will meet weekly or monthly as project needs evolve. The Project Team will oversee the daily operations of the project and will review time and activity allocations, data collection techniques, and will assure the smooth and efficient operation of project activities at a day by day level and will meet weekly. During the last 6-8 months of the project, activities will focus primarily on the analysis of data, preparation of abstracts and reports, and manuscript writing (see Gantt chart below). The key facilities and

personnel needed for the proposed work are available and in place: 1. Personnel. A team consisting of individuals who have been working together for many years has been formed that is fully capable of performing the research; 2. Space. Each of the study sites has sufficient office space for each of the investigators and research staff; and 3. Computer. Data management and analysis will be performed using existing computer facilities. Software is already installed for database management, statistical analysis (SPSS and SAS), qualitative analyses (Atlas.ti), and surveys (licenses for Inquisite).

**Table 9.** Steering/Advisory Chair PCS-OSC (L. Pogach); Committee: DM-QUERI (C. Richardson));); PCS-OPC and OSC (S. Kirsh) PBM (Ginny Torrise) VA National Center for Patient Safety(D. Hoover); Office of Academic Affiliations (S. Gilman) Ann Arbor COIN (E. Kerr)		
Internal Steering Committee: D. Aron (Chair)		
Quantitative Team– Chin Lin Tseng, Dr. PH	Qualitative – Julie Lowery, PhD	Operations – David Aron, MD, MS; Project Mgr – Sherry Ball, PhD
O. Soroka<br>TBN.	D. DiFiore, PhD; L. Stevenson, PhD; M. Montpetite, MBA	S. Watts, RN, CNP, ND; K. Pascuzzi, Pharm D; J. Shell-Boyd

GANTT CHART

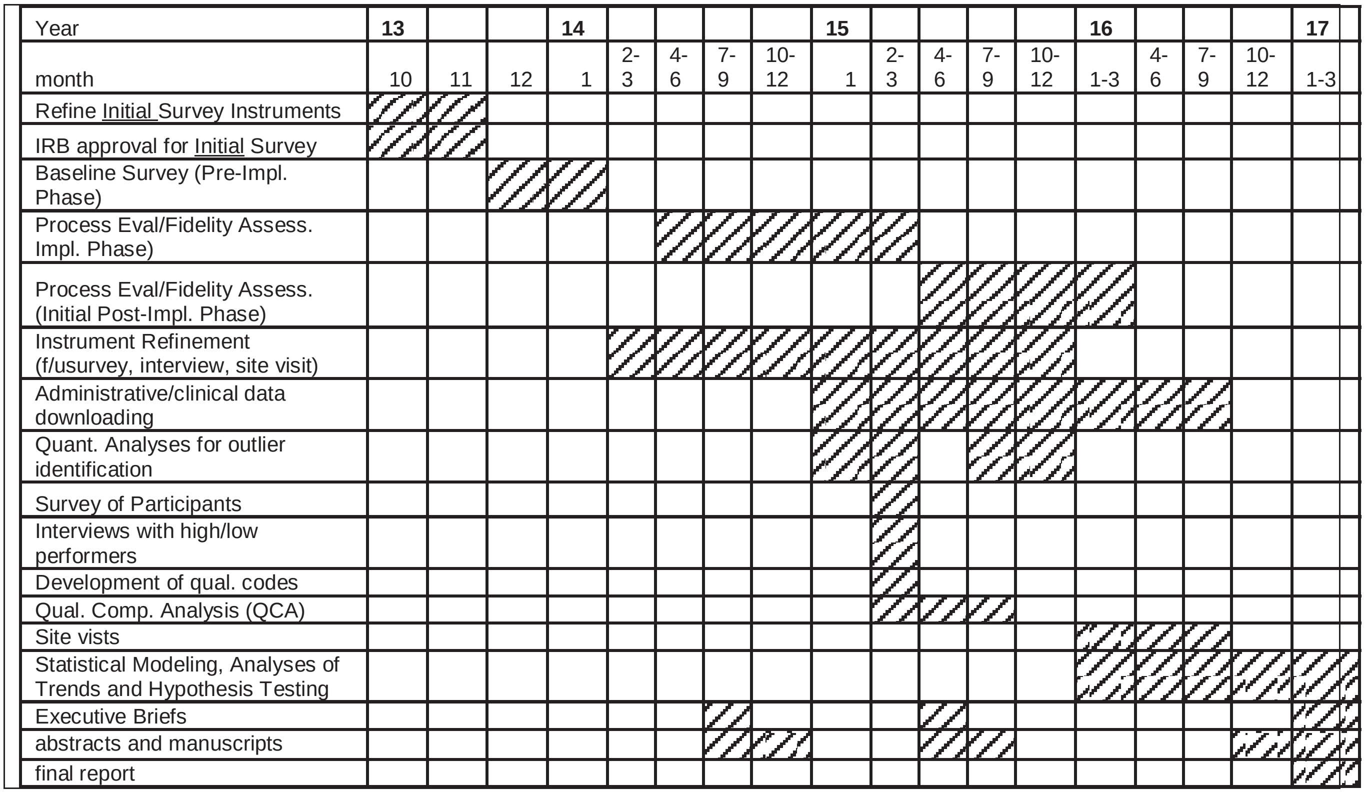

Human Subjects Considerations:

a. **Risk to Subjects.**

Human subjects' involvement and characteristics.

This project is essentially an observational study of a natural experiment - the VA national Choosing Wisely initiative that will roll out Jan. 30, 2014. Thus, the interventions are not under the control of researchers, but are amenable to research which uses the variation in exposure that they generate to analyze their impact. VISNs will choose one among a limited number of overuse/low value care issues to address. Thus, implementation will naturally vary among facilities both in terms of what is implemented and when it is implemented. As such, this observational study will assess effectiveness as contrasted with establishing causality.

The primary potential risks are breach of confidentiality; psychological discomfort; and the inconvenience of filling out forms and participating in interviews and site visits. Nevertheless, no adverse affects are anticipated with the proposed study. Although the target patient population is at high risk for hypoglycemia, so that adverse events are likely, these result from the patients' condition, something the initiative is trying to reduce. Thus, this risk is independent of the research project and will not be affected by the research itself.

Breach of confidentiality may occur when working with large databases. However, we have considerable experience in this area. Analytic files will not include identifiable data elements. Results will be aggregated at the facility level and clinic level, but not at the provider level. Information will be collected from healthcare professionals via on line survey or interview regarding the implementation of the Choosing Wisely initiative. Names will be kept in secure files for tracking purposes only. Results will be presented only at the facility/clinic level potentially stratified by clinician type. Similarly, at site visits, we will note personally only by job title, e.g., clinic manager, clinician. Results at this level will be reported only in aggregate.

The process of unlearning a standard practice that is now out of date could be uncomfortable. However, this is inherent in the initiative itself, although the interviews could exacerbate the discomfort. However

The study activities will be conducted as follows: A baseline assessment will be conducted followed by a 1-yr implementation phase and an 18 month maintenance phase. During the baseline period, we will assess practices as they relate to potential DM overtreatment. We will then conduct an ongoing fidelity assessment with surveys every three months to determine which components of the intervention are implemented, when they are implemented and whether they are maintained. Overtreatment rates for all VA facilities with >100 patients with diabetes will be assessed at quarterly intervals during the pre-initiative period (2009-1/30/2014) and for the following 2.5 years. Trends of overtreatment rates will be analyzed using a serial cross-sectional design. A similar approach will be use for rates of undertreatment and use of newer medications. One year following initiative initiation, high and low performing facilities will be identified to provide data for crisp set qualitative comparative analysis (QCA) This method, based on set theory, bridges quantitative and purely qualitative approaches and will be used to compare cases systematically and identify cross-case patterns and set-theoretic membership with outcomes, i.e., identify which configurations of intervention components with higher performance. At year two, high and low outliers will be identified and the findings of the QCA will be assessed in depth via site visits with artifact collection, procedural observation, and semi-structured interviews. selected for site visits that will include direct observation and interviews with clinicians including (CPSs, PCPs, and clinic managers) will be conducted at high and low performing sites to enhance understanding of contextual factors.

b. Adequacy of Protection from Risks. First we will work with the relevant IRBs to clarify which individual health care providers are considered human subjects for research purposes. For the latter, we will obtain Informed Consent: Oral or written consent (as advised by the IRBs). Most surveys will be anonymous, but coded by broad categories where there are >10 individuals). Completion of the survey or participation in an interview will imply consent. When linkage with other data is required, we will use the consent procedures as above. All relevant methods of ensuring privacy will be utilized.

c. Potential Benefit of the Proposed Research to the Subject and Others. The risks of this study are relatively minimal; the benefit to an individual is also minimal, although completion of a survey could encourage self-reflection.

d. Importance of the Knowledge to be gained. Identification of optimal strategies for the implementation of national initiatives and de-implementation of outdated or low value practices.

Inclusion of Women.
Aggregate data will include both men and women.
