# Supplementary material for: De-implementation of inappropriately tight control (of hypoglycemia) for health: protocol with an example of a research grant application
Source: Implement Sci. 2014 May 19;9:58. doi: 10.1186/1748-5908-9-58 (PMC4046046; doi:10.1186/1748-5908-9-58)
Supplement: Additional file 5 — Critiques of the initial submission. [file 1748-5908-9-58-S5.docx]

**Summary Bullet Points**

1. The proposed study is well aligned with the DM QUERI aim of promoting evidence-based approached to treatments.
2. The approach of “de-implementation” is novel and important to investigate.
3. Obtaining the appropriate sample size for this study may be problematic.
4. The power calculations suggest that the study would be under-powered to even address Aim 1. This should be addressed prior to resubmission.
5. Research Question 2.1 and 2.2 should be dropped from this study.
6. Drop Hypothesis 1.3 or justify that these medications will be available in VA.
7. The quantitative analysis and qualitative data collection methods are unclear (see critiques for in-depth explanation).
8. It is unclear if there is an alternate plan to address variation in safety culture and ORCA, or lack thereof, amongst facilities.
9. Study team FTE, in particular the PI at 10%, does not seem sufficient (see critique 4).

**Critique 1**

1. **Significance and alignment of proposed specific aims with QUERI and VHA goals.**

Appropriate control of glucose in a condition with something like a 25% prevalence in Veterans is an exceptionally strong target

1. **Impact.**

De-emphasizing tight control will have a large impact in terms of hypoglycemic events, which the applicants provide good estimates can occur in up to 15-20% of patients.

1. **Approach:**

I‟ll note a number of issues, below.

1. **Personnel and Management** Strong team, linked with the Diabetes QUERI
2. **Human Subjects Protection:** No comment.
3. **Budget:**

No comment

**7. Impact and Sustainability:**

This proposal is to assess a coming change in VA policy that seeks to de-emphasize so-called “tight control” of diabetes, with target Hgb A1c levels of less than 7%. In late January of the coming year, VHA will roll out a national initiative called Multifaceted Hypoglycemia Risk Reduction Intervention, and this application is to evaluate the effectiveness of this, in particular looking for context-sensitive effects and to assess which components (of the multiple components) might be most responsible for an effect. The VHA intervention includes the use of a clinical reminder, the use of clinical champions, academic detailing, audit-and-feedback, decision support, the use of nurse case managers and pharmacists, and educational materials. The application plans to assess the effects of the intervention on the proportion of patients with diabetes and certain factors that puts them at higher risk of hypoglycemia (presence of dementia, renal disease, age, and the use of insulin or sulfonylureas) and a Hgb A1c of less than 7.0 (or 6.0 or 6.5, they will assess various thresholds). And then, as well, the potential for inappropriate relaxation of glucose targets, looking at the proportion of patients whose A1c exceeds 9.0. The application proposes to assess context sensitivity by looking at several measures of context (safety culture, organizational readiness to change) and also the intensity of eh adopted intervention (how many of the components are implemented), across the system, and then in a narrower number of facilities use qualitative methods to try and identify factors related to success in high deviant facilities (one whose performance exceeds that of their VISN). They also plan to assess whether the process of de-implementation is different than the process of implementation.

What I like about this application is that it is taking advantage of something VA is going to do anyway, and is trying to study it to learn something both about the target intervention and the process of implementation in general. It is being led by experienced investigators who are solidly locked in to VA research and operations.

The investigators make much of their belief that the process of de-emphasis will differ from the process of emphasis. I am willing to go along with them in terms of this being a testable hypothesis, but do note I am far less convinced that the process, either conceptually or in its practical application, is all that different from any other provider-based or organizational-based behavior change. In concept this is no different than a choice of intervention A vs intervention B, where the prevailing opinion has been A is better than B, and now new data suggests B is better than A. The authors themselves point to HRT in women as one example, and that practice seems to have changed very rapidly as a result of the publication of the WHI, changes in practice guidelines and performance measures, and widespread publicity. If there were any clinical reminders or decision support or audit-and-feedback used to help make this change, I am unaware of it. Recently, VA has also de-emphasized lipid targets, going from specific targets to instead a certain dose of statin therapy. The same is coming for PSA testing, with experiments going on now with interventions to decrease the use of this. In the broader health care setting, there is the “rise-and-fall-and-rise of carotid endartectomy”, the old NEJM article that documents how this went up-and-down-and-up-again following enthusiasm, publication of studies questioning its utility, and then major clinical trials establishing a benefit in certain patient populations. I suspect that the factors associated with successful “de-emphasis” are going to look very similar to the factors associated with other forms of behavior change, such as the work of Richard Grol and the factors associated with Dutch GPs following guidelines. These are going to include things like whether the action is consistent with their understanding of professional norms, whether it involves something easy like writing a prescription or ordering a blood test or whether it is something more time consuming like counseling, and (in America) whether there is money involved. So stopping writing HRT prescriptions is a much easier change to make than stopping giving PCI for persons with stable angina,

and is a big reason why the publication of the WHI made a big difference in practice while the publication of COURAGE has not. So – I don‟t share the authors belief that this kind of change is so fundamentally different than the other kinds of change that we‟ve been trying to influence for years. But I do agree it is something testable.

There are a number of methods issues that the applicants will want to consider as they plan and perform their study. The first is their power analysis. I can‟t understand how they did these calculations, but they state that the sample size will have adequate power to detect a 35% change in rates if the preimplementation rate is 40%. Yet two paragraphs earlier the application presents the VISN 12 data where the pre-implementation rate was 34.5% and a year or more into the intervention the rate was 30% - a 13% relative change, which is far, far away from the 35% change they‟ll need to have in order for them to have enough power. And this is before the clustering. So I either didn‟t understand something in these power calculations or the applicants are going to need to have to do something – a lot of something – to have enough power to do what they hope to do. With a nationwide VA rollout happening, it seems to me there ought to be plenty of power available, so I am presuming this is a fixable problem.

Another methods issue they are going to want to consider is the recent change over time in their outcome measure at the various sites. Maybe what I am about to propose may not actually exist in their data, but here it is. My hypothesis is that it will be easier to “deemphasize” the glucose targets in facilities that made recent efforts to “emphasize” those targets, as compared to facilities which have been and continue to have high levels of low A1cs in their target populations, This is based on the following: every study I have ever seen of an organizational change like clinical reminders or decision support has shown when the reminder and support are turned off then behavior quickly reverts to its baseline characteristics. So facilities that ^„^did something” in order to push providers and patients to lower targets are going to have that behavior more easily reversed by de-emphasis than our facilities where low targets have been “hardwired” into their organization for years (for example due to the influence of a charismatic clinical champion for low targets). So, if the applicants could go back in time and look at the past 5 years or so of data and see if their study facilities differ in the change over time in terms of their outcome measure, then I would include this as a predictor variable in the contexts for assessing effectiveness.

Speaking of the contexts, the methods in this section is resting on the hope that these contexts are going to naturally differ among the 30 or so sites that they select for inclusion in their more detailed analysis, which is based on a “high and low performer” set of criteria. Maybe this is going to be the case, but then again maybe it‟s not. That remains to be seen, and they have to be prepared with a plan B if it turns out they don‟t have the variance across sites in terms of the safety culture or commitment to quality measure.

Lastly, I could not understand at all the text on pages 29-33 in terms of research question 2.1, “Which configurations of the MHRR intervention components and which factors are associated with greater reduction...”. It is very hard for me to believe that they are either going to get the necessary spread of the intervention components across their 30 facilities (that is, the naturally occurring variation is going to provide them with an adequate distribution to try and estimate effects for different components or combinations of components) or have the degrees of freedom they are going to need to do hypothesis-testing work in this space. I‟d think you‟d need more like hundreds of facilities rather than 30 in order to be able to do this. This may be a section to consider cutting from the plan.

One last point to bring up is that this application had a much-worse-than-average use of the English language, with virtually every page having typos and grammatical errors, like “For example, a recent study reviewed of articles in the New England Journal of Medicine...” and “There are large knowledge gaps are.” And “we have considered two commonly mentioned methods (for person level of analysis)

are propensity score analysis and instrumental variables approach”. It really detracts from the proposal and these applicants are advised to do better next time.

**Overall Impression.** See above

**Key Strengths.**

1. A time sensitive evaluation of a major VA rollout for an important patient population
2. Looks at overuse of care
3. Experienced investigators locked in to VA research and operations

**Key Weaknesses.**

1. Sample size may be a problem
2. Aim 2 Research questions 2.1, 2.2 and 2.3 depend on variation among facilities in terms of predictor variables that may not actually occur
3. The plan for testing which configurations

**Critique 2**

1. **Alignment of proposed specific aims with QUERI and VHA goals.**

The Diabetes QUERI and the leadership within PCS are both very focused on this, based on letters and reinforced by the willingness of Dr. Richardson of the Diabetes QUERI to commit financial resources to facilitate gathering baseline data necessary for the present project. In addition to the formal letters, there is considerable evidence that the VA has made this a priority inasmuch as the formal “de-implementation” is an initiative – the Multifaceted hypoglycemia risk reduction intervention (MHRRI) - which is being rolled out by PCS as a VA wide, but optional program.

In addition, the investigators supply considerable evidence that this is a significant problem, both overall and in VA, and that it has important negative consequences for VHA – that is, it should be a VHA goal.

1. **Evidence base is adequate for implementation.**

This is an area where they do not make much of an effort to convince us that the intervention is likely to be an effective one. They do note that the components of the MHRRI generally have an evidence base, and they do note that multi-component interventions are generally considered superior to interventions that have just one component. There is relatively little discussion of whether the MHRRI components are the ones that one should use. Moreover, the descriptions – Clinical champions, Educational outreach, clinical reminder, Audit and feedback, decision support, multidisciplinary care and educational materials – are insufficiently precise to give me much of an idea about what is planned. The description of these interventions is very cryptic – sometimes no more than the name - and there is no discussion of the evidence that supports the various components and how it is consistent with the approach planned with the current project. To take the first example, details might include how the clinical champion is to be identified, any support to be given to a clinical champion, whether the clinical champion will have protected time for that role, etc. They suggest they will gather such detail in follow up interviews at outlier sites, but the evidence base is inadequate for some versions of this intervention.

In their defense, they can argue that the strength of evidence is not all that important, since the VA has decided to invest considerable resources in the deimplementation project. Thus, an effort to understand what about the MHRRI did and did not work is warranted simply on that basis.

The evidence that iatrogenic hypoglycemia is common, morbid, associated with attempts to achieve tight control (or generally poor control) and important is quite strong. The face validity of discouraging sulfonylureas or insulin in this setting is high.

1. **Research aims and methodology are appropriate:**

The specific aims (SA) are clearly focused on understanding the success of an approach that someone else has devised. Aim 1 asks an appropriate question regarding whether the MHRRI was effective when implemented, for better or worse. Their analysis is quite complex and involves trying to determine when the intervention is actually happening at an individual VAMC, but in the end they generate a start date and will determine if, after that date, there is a change in several measures (H1.1., 1.2, 1.3) that are either targeted or likely to be affected in an effort to hit the target. I would argue that the MHRRI starts on 1/30/14 regardless of when individual facilities put in place any of the components that are planned. This would also simplify the analysis – was the program able to change slopes in VA overall. The idea that some facilities will be more or less successful is all part of the answer.

The second aim is more interesting for future use. In this aim they will try to determine why changes were more favorable in some facilities than in other. They argue that their mixed methods are particularly appropriate, since the complex intervention is one that is not amenable to simply examining the regression coefficient on the particular component being assessed. That said, they will provide us with the regression co-efficient and also look at the data in less quantitative ways, using in particular “Qualitative Comparative Analysis.”

The third aim of the concept paper is now termed a Research Question. It is “How does de-implementation differ from implementation from a clinician perspective?” Although this seems an appropriate question for a QUERI project, they provide no information on the methods that will be used to address it.

1. **Research Design:**

They are clearly at the mercy of the VA‟s decision on how to implement MHRRI. They will get a non-randomly selected subset of VAMC and CBOC‟s that will choose to focus on this *Choosing Wisely* topic, while others focus on other topics – we are not told what they will be. Therefore, they are going to try to learn as much as possible from the facilities that choose to adopt. They are further handicapped by needing to cobble together a description of what each facility is doing based on reports from surveys of clinical pharmacy specialists and clinical managers. It appears that the surveys are going to continue to be repeated at some interval since they will define a facility as participating when it achieves 3 intervention components based on survey responses.

Once a facility is categorized as participating, they will examine the proportion of patients who are in a group of diabetics at high risk for hypoglycemia who receive insulin or sulfonylurea and have HgbA1c values below a specific threshold, comparing the proportion before and after intervention. Quite rapidly they will identify facilities with the lowest proportion of at risk patients with A1c below the threshold and then consider these facilities to be high outliers, or positive deviants if they are high outlier facilities but in a VISN that is not particularly good. These designations will be made based on absolute numbers rather than changes, it appears.

The survey data will also provide information about whether the facilities use specific intervention elements. They recognize that the determination of whether an element is used could vary among interview respondents, so they propose that they will clarify just which intervention elements are implemented during the telephone calls they will make to the high and low performing outliers.

1. **Human Subjects Protection:**

They acknowledge the fact that employees in this study are human subjects and their privacy and risk of participation are worthy of consideration. They take adequate precautions.

1. **Project Organization and Management.**

This group has worked together on projects for years and contributed broadly to the literature regarding appropriate diabetes management, both in the VHA and in general.

**7: Evaluation Plan:**

Their mixed methods approach is appropriate. They note that their quantitative tests will be underpowered to detect all but the most remarkable differences between intervention and control sites in the changes in overtreatment among at risk patients. This is going to be even more of a problem for endpoints such as undertreatment and increases in use of certain newer agents that do not put patients at as much risk of hypoglycemia. This power is likely to be further compromised since they propose to use a range of definitions of overtreatment, and examine multiple subgroups which are at increased risk of hypoglycemia. Although they don‟t describe corrections for multiple comparisons, their quantitative analysis will need to be interpreted in light of their very wide set of proposed ways of defining the study population of interest, and the primary outcome of overtreatment.

They hope to make up for this by gathering extensive qualitative data on certain high and low outliers. Although they don‟t have a lot of detail on how they will identify these sites, and how many there will be, we do know they will be drawn from the top and bottom 5% - this would 6-7 in each group if they lumped CBOC with facility, leaving an N of 131, which seems their first choice, though apparently subject to revision if it seems appropriate. But since they have multiple definitions of the outcome (different A1c cutpoints) and at risk population (age, dementia, comorbidity) there may be some facilities that are outliers for some endpoints and not for others.

They don‟t have much detail about how they will gather information about the how of implementation at sites that are selected, although a large number of individuals will be asked to complete surveys and a smaller number will be asked to participate in phone interviews. Draft surveys are provided in an appendix, but it is hard to line them up with all the surveys that are described. Examples of similar interview guides for other projects are presented as they plan to select some of the items for use in this project. For the analysis of this data, they describe use of qualitative comparative analysis (QCA), a method that is very tolerant of a large number of important variables that may be present at only a few facilities; but it is not clear what variables will be considered as candidates for inclusion in the model.

After this analysis has identified constructs associated with being a high performing site, they plan site visits to these high performing sites to obtain detailed information on what those constructs look like at the site, with an aim to developing a toolkit that other sites can use. Although the site visits are not descried in much detail they plan to visit clinics and collect artifacts e.g, screen shots of CPRS, educational materials, etc. They will also interview clinical pharmacy specialists, primary care providers and clinical managers. The details of how many sites they will visit, for how long and who will make the visits is not clear. The analysis of this data is not described.

The wide range of analyses proposed, the large number of variables described, and the range of potential inferences to be made make me concerned that the data synthesis step will be difficult. While the use of mixed methods is an appropriate response to this complex intervention in a complex system, I was unable to convince myself that a synthesis of the various methods will be feasible.

1. **Budget:**

They have only a relatively small amount of time from the QCA expert, and from Dr. Lowery of the DM QUERI for interpreting the large volume of qualitative and quantitative data that is to be synthesized for the second Aim. It is not clear who will be the prime leader of that analysis, although a third PhD health services researcher is also involved. She, however, will have responsibility for actually conducting the interviews and helping to design the surveys.

I am also concerned about the relatively limited quantitative budget for the repeated and extensive looks at the data that will be needed in the first year to identify the high and low outliers. During this year the Masters and PhD analysts have just 10% time apiece.

1. **Impact and Sustainability:**

It is not clear that this project will have a sustainable impact on practice across VHA. There is a close connection to DM leadership, but

**Overall Impression.**

This project seems to be too broadly focused. A wide range of analyses are planned with limited clarity on how they will be synthesized. I am particularly concerned that serendipitous associations will be viewed as important, while important ones may be missed, especially at the early stages when quantitative associations among constructs that are admittedly measured with considerable error are made and then lead to in depth attempts to better understand the associations.

**Key Strengths.**

1. Longstanding relationship among the investigators.
2. This is an area where an effective intervention is likely to have important impact on veterans‟ health and where VHA operations is planning to take action.
3. The idea of de-implementation is one that we will see again. It is important to take steps to understand whether there are important qualitative differences between this and implementation.

**Key Weaknesses.**

1. I am not convinced that there is enough focus to allow synthesis.
2. The description of the quantitative analysis, although long and detailed, is not described in a way that allows me to judge whether it will test the specified aims. The power calculations suggest that it is not powered to address even the first aim. The second aim is obviously beyond quantitative reach, unless very fortuitously large numbers of facilities end up making very similar choices.
3. The qualitative data collection is not described in adequate detail. The transition from data assessment and coding to QCA is not adequately described. Again, I would feel better if there were some ideas going into the project regarding specific questions that should be addressed.

**Critique 3**

1. **Alignment of proposed specific aims with QUERI and VHA goals.** No comment
2. **Evidence base is adequate for Implementation:**

The evidence base that strict glycemic control is not beneficial and potentially harmful in certain subgroups with DM is sufficient to lead to changes in CPGs (even among true believer organizations such as the ADA). The evidence comes from several large RCTs and is convincing. Although the evidence is not directly from VA populations, there is no reason it would not apply to the VA population. A potential weakness in the evidence base and study logic is that the target population (and the outcome) is those who are *potentially overtreated* – as we don‟t know and won‟t know from the data collected if patients are having hypoglycemic episodes. Nevertheless, data are presented that hypoglycemic episodes, including those that are serious in nature occur with sufficient frequency to justify the effort to reduce even the proportion who are *potentially overtreated*.

The evidence base for the MHRRI is less well established. Less is known about how to design and implement programs aimed at decreasing the use of firmly established provider behaviors. Although this is a weakness of the implementation science field, there is no information provided to suggest that the MHRRI was tailored to special issues related to de-implementation and the investigators do not draw sufficiently upon prior examples such as changes in data about medication effects (e.g., antiarrhythmics during acute coronary syndrome, estrogen) or changes in guideline recommendations (e.g., dropping need to monitor LFTs for patients on statins). The investigators state (and I agree) that the intervention meets the definition for a complex intervention, but the intervention while multicomponent is not described in detail and appears to be a hodgepodge of elements. Some of the educational materials appear weak or overly complex and are constrained by VA‟s algorithmic approach to CPG development. Finally, a similar intervention piloted in the investigators VISN only produced modest results.

1. **Research aims and methodology are appropriate:**

The research aims are stated clearly, important, and are a strength of the proposal. The aims address the intended intervention effects (overtreatment rates), unintended effects (under treatment, change in use of expensive hypoglycemic) and organizational and provider factors related to implementation processes and implementation success. An important and novel aspect of the proposal is the focus on understanding factors unique to “de-implementation.” There is clear support from the DM QUERI (including monetary support), the Choosing Wisely Initiative, and Primary Care Operations. Patients are not represented in the proposal – due to IRB complexity – but the investigators plan to submit a separate proposal to address patient perspectives. While the rationale for not involving patients directly is understood, this is a relative weakness as patients‟ attitudes, priorities, and knowledge may be important barriers to achieving a reduction in over-treatment.

The overall plan is linked to a sensible theoretical framework, although surprisingly it omits contextual issues such as degree of local change (e.g., provider turnover), provider morale, and competing priorities – issues known to be important to implementation in primary care settings. However, some of these issues are addressed in the specific data collection measures. The design will capitalize on natural variation in the success of implementation.

1. **Research Design**:

Overall approach: The overall approach to evaluate a natural experiment is appropriate – using time-series analyses and mixed methods analytic approaches. Analyses attempt to adjust for baseline inequalities likely to be observed between groups, and the effects of time. Evaluating the entire system (131 facilities with >100 patients with DM) with deep dives at selected facilities is a strength. Overall, the approach is consistent with best practices for evaluating organizational interventions.

Patients/sample: There appear to be ample patients (>285,000 with DM and at elevated risk) in the 131 facilities. Despite this apparently large cohort, power calculations presented show sufficient power to detect only large effects. For each of the power scenarios presented, the effects are larger than those (4.5% absolute difference) observed in the originating VISN, a VISN that presumably has motivated leadership and clinicians for this particular issue.

Hypothesis1.3 relates to the use of high cost medications with a lower propensity for hypoglycemia. Currently these medications are highly restricted or unavailable through VA formularies. Unless this changes, it will be difficult or impossible to address this hypothesis.

A concern is the lack of specificity in describing the intervention components. Although the intervention benefits from being multifaceted, and the basic components are described, details are given only for some elements and some of the detailed materials (e.g, the provider education materials) do not seem well suited to the task. Further, some changes attributed to the MHRRI program (e.g., changes in laboratory reporting format) have long been in place, at least at some VA sites, and could confound the association with the intervention.

What level of national support will be provided to the program? Will CPRS clinical reminders be robust, easy to use and acceptable to clinicians, and standardized across VISN or as with some reminders – subject to local adaptation. Will clinical champions be given training, time and resources to truly champion implementation or will this be a simple add on duty for overcommitted clinicians? Decision support seems rudimentary. Academic detailing and provider education may be misaligned for the majority of primary care clinicians since the survey data presented suggest only about 1/3 of physicians have a knowledge deficit. Audit and feedback seems potentially robust and may be one of the most important elements of the MHRRI. Although it is true that we know less about designing interventions for “de-implementation” the QI community has experience around some clinical issues (e.g., antibiotic prescribing for acute respiratory illness, estrogens for osteoporosis, glitizones for DM) where the goal has been to reduce use – and this literature is not cited to inform the current intervention.

Intervention fidelity is assessed q3 months – a critical component since the intervention may simply fail and measuring fidelity is a critical component to understanding the success/failure of the intervention.

The measure of exposure dose is crude (elements * months implemented). It would not be surprising to see no or a weak association with the independent variables (safety and quality commitment).

As the MHRRI intervention has not been fielded, there is uncertainty over which VISN/facilities will choose to focus on DM over-treatment. This uncertainty generates some confusion in the proposal in regards to the intervention vs comparator sites. For some analyses, the comparators are those implementing other CW initiatives. Since focusing on another QI initiative may retard implementation of concurrent clinical programs (e.g. overtreatment of DM), should another comparator be those sites who do not participate in the CW initiative?

Measurement:

a. The organizational, provider factors and outcomes measures seem well chosen and feasible.

1. The process for collecting provider level data through surveys is standard but given low rates of MD survey response, the potential for high non-response rates is a concern.
2. The primary quantitative outcome measure –potential over-intensive glycemic control (assessed quarterly) – seems conceptually sound although this measure would be stronger if we had validation data showing the linkage to clinically meaningful outcomes. Based on the data presented for the 2100 PACT assigned patients, severe episodes (32/2100) appear infrequent.
3. Although not completely describe in the proposal, the measures appendix show careful consideration for contextual factors.
4. It is unclear if surveys limited to clinic managers and PharmD will provide valid estimates of local attitudes towards A1c targets and the exposure intensity.
5. Qualitative data collection using semi-structured interviews and site visit guides seem appropriate.

Timeline: The timeline is well summarize in a GANTT chart and appears sensible.

Dissemination plans are adequate and standard.

1. **Human Subjects Protection**:

Appropriate measures are described to protect confidentiality. Risk of harm is minimal. No concerns.

1. **Project Organization and Management**:

The project leaders are stellar and have the content expertise (both in DM and implementation science) and research track record to successfully lead the project. The project management plan is sensible. Other team members have the skills and experience to be successful in their respective roles.

1. **Evaluation Plan** See above comments
2. **Budget**:

Although relatively expensive since this project is restricted to evaluation of a VHA central office initiative, the budget seems appropriate and adequately justified. It is likely that patient perspectives could be sought and incorporated into the evaluation for little additional expense.

1. **Impact and Sustainability**:

It is clear that overly tight glycemic control is problematic for patients with DM overall and for Veterans with DM. If the MHRRI is effective, it will decrease the rates of overtreatment and should decrease the rates of important adverse medication effects. There is the potential for downstream cost-savings (less medication, fewer complications of hypoglycemia) and improved veteran health status.

The proposal has the potential to: a) better understand the effectiveness of the national choosing wisely initiative, b) advance implementation science through a focus on differential measures required for de-implementation. If the conceptual framework were to classify different types of interventions (e.g., simple medication changes, provider behavior changes, complex organizational changes) into a framework that included the MHRRI initiative and allowed other interventions to be mapped to this framework, then the contribution to implementation science would be more generalizable and robust.

**Overall Impression**:

Overall, this study proposes to evaluate the effects of a national initiative to implement revised guidelines for glycemic control in patients with diabetes. The initiative and study is particularly interesting because the change is guideline is for more permissive control in a large subset of patients. Strengths of the proposal in include the consistency with QUERI and National VA priorities, sound overall research design, and many best practice approaches to evaluation. Weaknesses include: inadequately description of the national MHRRI initiative, likely small impact of the initiative (and thus insufficient power to test the aims), lack of involvement of patient stakeholder groups, and a target population and outcome of “potentially over-treated” without clear knowledge of rates of hypoglycemic episodes or its related harms. Although this study has the potential to advance knowledge of implementation science, these weaknesses must be adequately addressed to invest in this relatively high cost evaluation.

**Key Strengths and Weaknesses**:

Strengths:

1. The proposal is aligned with the broad DM QUERI aim of promoting evidence-based approaches to treatment and to reduce the complications of DM. The study aims are important and include the novel concept of “de-implementation.”
2. Overall, the evaluation approach is consistent with best practices for evaluating organizational interventions.
3. The research has good support from the National PC office and QUERI.

Weaknesses:

1. An important potential weakness in the evidence base and study logic is that the target population (and the outcome) is those who are *potentially overtreated* – as we don‟t know and won‟t know from the data collected if patients are having hypoglycemic episodes.
2. While the rationale for not involving patients directly is understood, this is a relative weakness as patients‟ attitudes, priorities, and knowledge may be important barriers to achieving a reduction in over-treatment.
3. The intervention is not adequately described and there is important uncertainty about the support for the intervention and its likely effectiveness. The pilot intervention only showed small effects and power calculations describe scenarios that are larger than those observed in the pilot.

**Critique 4**

1. **Alignment of proposed specific aims with QUERI and VHA goals.**

This project is well aligned with QUERI and VHA goals and well-timed with the upcoming implementation of the MHRR intervention.

1. **Evidence base is adequate for implementation.**

The risk of hypoglycemia is well established. There does not to appear to be as much evidence (pilot data) to support the specific, proposed intervention. However, the investigators will make efforts to determine the attributable impact of the components of the intervention and all of the intervention components are generally well established in many other diseases and processes.

1. **Research aims and methodology are appropriate:**

The aims and methods are generally appropriate but I do have some specific concerns. 1) some of the variables and outcomes are not well defined. For example, kidney disease will be defined as a creatnine of 1.7. Is this a one-time measurement or an elevated measure sustained over a period of time? What if it was elevated in the past but has since normalized? 2) I have the same questions for A1c and further, how are patients whose A1c measures fluctuate between different categories handled during data analysis? 3) It was not entirely clear (at least to this reader) that the outcome of adverse events will be measured and whether there is sufficient power (or accuracy in the data) to allow this. 4) I am concerned that the surveys and role of the pharmacists to provide the lists of overtreated patients at each facility may prove a) to be too burdensome and b) may be fraught with missing data. 5) The proposal notes that there will be a manual review process to assess patients for receipt of non-VA hypoglycemic medications. It is not clear what specific part of the medical record will be used (e.g., progress notes, med reconciliation notes, etc), how much of a burden this will be to the study and how much missing data is anticipated. 6) the top/bottom 5% of the facilities will be selected for further study to identify the barriers and facilitators to implementation, but from the proposal, it appears that facilities will be assessed in the context of the performance of their VISN. Perhaps I am not completely understanding the math, but I am concerned that if a VISN is high or low performing, facilities selected may not be true outliers when compared with the spectrum of VHA facilities. and 7) the pre/post intervention indicator variable appears to be incorrectly assigned in the bottom paragraph on page 27.

1. **Research Design:**

The design appears appropriate but I agree with other reviewers‟ concerns about sample size and the uncertain ability of the project to determine the attributable impact of different components of the intervention.

1. **Human Subjects Protection:**No concerns
2. **Project Organization and Management.**

A very well qualified teams has been assembled. Program office support and collaboration is extremely strong. I am very concerned that the surveys and other involvement asked of pharmacists and others may be too burdensome to ensure accurate/timely data needed for the study is received.

7: **Evaluation Plan:**

I am concerned that the dissemination plan lacks sufficient detail in what actions specifically will be taken and how the dissemination will be performed. Interestingly, neither front line primary care providers nor endocrinologists are listed in the table of end users.

1. **Budget:**

No concerns

1. **Impact and Sustainability:**No comment

**Overall Impression.**

This is a well-timed project focusing on a very important, prevalent problem. The study team assembled is excellent as is the program office support and alignment with VHA goals. My enthusiasm is somewhat tempered by the lack of detail in many critical areas of the methods, the possible over­reliance on assistance from facility pharmacists and unclean reasons for not including frontline staff in the dissemination plan.

**Key Strengths.**

1. important clinical topic, aligned with QUERI goals
2. well timed with PCS initiative roll-out
3. Excellent, qualified team

**Key Weaknesses.**

1. Incompletely described outcome and key variable definitions
2. High reliance on frontline staff to volunteer in completion of surveys and interviews

**MEETING ROSTER**

**HQ1 QUERI SDP REVIEW
Quality Enhancement Research Initiative Parent
Office of Research & Development
Workgroup
HQ1 R
October 23, 2013**

**CHAIRPERSON**

SINNOTT, PATRICIA LUCE, PHD

HEALTH ECONOMIST

VA HEALTH ECONOMICS RESOURCES CENTER (HERC) VA PALO ALTO HEALTH CARE SYSTEM

MENLO PARK, CA 94025

**MEMBERS**

GOLDSTEIN, MICHAEL G, MD

ASSOCIATE DIRECTOR

BAYER INSTITUTE FOR HEALTH CARE COMMUNICATION WEST HAVEN, CT

GRAHAM, GLENN D MDPHD, MD, PHD

DEPUTY NATIONAL DIRECTOR OF NEUROLOGY 810 VERMONT AVE

WASHINGTON, DC 20420

KREIN, SARAH , RN, PHD

CTR FOR CLINICAL MGMT RESEARCH, VA ANN ARBOR RESEARCH ASSOCIATE PROFESSOR, INTERNAL MEDICINE

DIVISION OF GENERAL MEDICINE

UNIVERSITY OF MICHIGAN

ANN ARBOR, MI 48105

MARTINELLO, RICHARD A., MD ASSISTANT PROFESSOR

VA CONNECTICUT

YALE UNIVERSITY

WEST HAVEN, CT 06516

POST, EDWARD P, MD, PHD

VA HSR&D (152) 2215 FULLER RD. ANN ARBOR, MI 48105

SCHNURR, PAULA P., PHD

HSR&D SMRB CHAIR

DEPUTY EXEC DIR VA NAT CTR FOR PTSD WHITE RIVER JUNCTION VA MEDICAL CENTER RESEARCH PROFESSOR OF PSYCHIATRY DARTMOUTH MEDICAL SCHOOL

WHITE RIVER JUNCTION, VT 05009

SHEKELLE, PAUL G., MD, PHD

DIRECTOR

SOUTHERN CALIFORNIA EVIDENCE-BASED PRACTICE CENTER

RAND

SANTA MONICA, CA 90401

SMITH, BRIDGET M., PHD

SOCIAL SCIENCE ANAYLST

HSR&D, CMCCC

EDWARD HINES JR VA HOSPITAL RESEARCH ASSISTANT PROFESSOR NORTHWESTERN UNIVERSITY

CHICAGO, IL 60141

SOX-HARRIS, ALEX , PHD

VA PALO ALTO HEALTH CARE SYSTEM PALO ALTO, CA 94304

TURVEY, CAROLYN L, PHD ASSOCIATE PROFESSOR

DEPARTMENT OF PSYCHIATRY UNIVERSITY OF IOWA

IOWA CITY, IA 52242

VANDEUSEN-LUKAS, CAROL , EDD

SENIOR INVESTIGATOR

CTR FOR ORGANIZATION, LEADERSHIP & MGMT RES VA BOSTON HEALTH CARE SYSTEM

CLINICAL ASSOCIATE PROFESSOR, HLTH POLICY & MGMT

BOSTON UNIVERSITY SCHOOL OF PUBLIC HEALTH BOSTON, MA 02130

WHITTLE, JEFFREY C., MD

PROFESSOR

HEALTH SERVICES RESEARCH & DEVELOPMENT ZABLOCKI VA MEDICAL CENTER

DIRECTOR, SOUTHEAST WISCONSIN ALLIANCE FOR TRANSLATING RESEARCH INTO PRACTICE

MILWAUKEE, WI 53295

WILLIAMS, JOHN W, MD

ASSOCIATE PROFESSOR

DEPARTMENT OF MEDICINE & PSYCHIATRY HEALTH SERVICES RESEARCH & DEVELOPMENT DUKE VA MEDICAL CENTER

DUKE UNIVERSITY

DURHAM, NC 27705

WILLIAMS, LINDA S, MD

ASSOCIATE PROFESSOR

DEPARTMENT OF NEUROLOGY, INDIANA UNIVERSITY RESEARCH COORDINATOR, VA STROKE QUERI INVESTIGATOR, VA HSR&D CENTER FOR IMPLEMENTING EVIDENCE-BASED PRACTICE

INDIANAPOLIS, IN 46202

WU, WEN-CHIH , MD

STAFF CARDIOLOGIST

HEART FAILURE CLINIC

PROVIDENCE VA MEDICAL CENTER

ASSOCIATE PROFESSOR, DEPARTMENT OF MEDICINE ALPERT MEDICAL SCHOOL, BROWN UNIVERSITY PROVIDENCE, RI 02908

**SCIENTIFIC REVIEW ADMINISTRATOR**

KILBOURNE, AMY M., PHD

ASSOCIATE PROFESSOR

DEPARTMENT OF PSYCHIATRY

UNIVERSITY OF MICHIGAN, SCHOOL OF MEDICINE RESEARCH HEALTH SCIENCE SPECIALIST SMITREC, VA ANN ARBOR HEALTHCARE SYSTEM ANN ARBOR, MI 48105

MCIVOR, LINDA

SCIENTIFIC REVIEW ADMINISTRATOR

HSR&D QUERI

DEPT OF VA, VETERANS HEALTH ADMINISTRATION WASHINGTON, DC 20420

Consultants are required to absent themselves from the room during the review of any application if their presence would constitute or appear to constitute a conflict of interest.
