# Supplementary material for: De-implementation of inappropriately tight control (of hypoglycemia) for health: protocol with an example of a research grant application
Source: Implement Sci. 2014 May 19;9:58. doi: 10.1186/1748-5908-9-58 (PMC4046046; doi:10.1186/1748-5908-9-58)
Supplement: Additional file 7 — Critiques of the revision. [file 1748-5908-9-58-S7.docx]

2/24/14

# Summary Bullet Points

Time sensitive evaluation by a strong investigator team of a major VA roll out for important patient population.


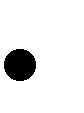


The key independent variable (site participation in risk reduction) for Aim 1 was not well-defined and rationale for choice of independent variables for Aim 2 was not clear.


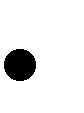


The project‟s lack of involvement of patient stakeholder groups may miss information pertaining to the program‟s success, especially in addressing patients‟ attitudes, priorities, and knowledge in achieving a reduction in over-treatment.


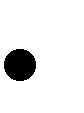


Suggest use of Statistical Process Control and Run charts as a means to identify whether or not the Choosing Wisely campaign had an effect (see critique 1).


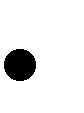


# Critique 1

1. **Significance and alignment of proposed specific aims with QUERI and VHA goals.**

Appropriate control of glucose in a condition with something like a 25% prevalence in Veterans is an exceptionally strong target

# Impact.

De-emphasizing tight control will have a large impact in terms of hypoglycemic events, which the applicants provide good estimates can occur in up to 15-20% of patients.

# Approach:

I‟ll note a number of issues, below.

# Personnel and Management

Strong team, linked with the Diabetes QUERI

# Human Subjects Protection:

No Comment

# Budget:

No Comment

# Impact and Sustainability:

This proposal is to assess a coming change in VA policy that seeks to de-emphasize so-called “tight control” of diabetes, with target Hgb A1c levels of less than 7%. Approximately April 1st of this year, VA is going to rollout a “Choosing Wisely” toolkit that will focus on trying to reduce inappropriate use in two areas: potentially inappropriate aggressive lowering of HgbA1c in certain patients with diabetes and the overuse of MRI examinations in patients with low back pain. This SDP seek to evaluate the effectiveness of this, by comparing rates of performance in facilities that voluntarily select the diabetes

target, the back pain target, both targets, and no targets. As opposed to their earlier submission, which proposed some specific intervention components in order to try and achieve this “de-implemenation”, in this proposal this is left to the “toolkit to facilitate action [that] will be provided by VA.” The outcome measure remains conceptually the same, namely the proportion of patients whose most recent Hgb A1c is less than 6.5% (or 6% or 7.0% in sensitivity analyses) and (I think) one of age, dementia, and serum creatinine of more than 2.0; and then also the proportion of people whose last HgbA1c exceeds 9.0, (for inappropriate undertreatment). The plan is to use a number of predictor variables and see if they are associated with change over time, including participation in the Choosing Wisely campaign, commitment to quality, safety culture, “teaching intensity” (I like this one least, since the definition for this is so lame compared to their other variables like safety culture and participation in Choosing Wisely), and patient variables. They then plan to identify high and low performers and do some in- depth evaluations to try and figure out what might be going on that explains the results: what parts of the toolkit were implemented and which weren‟t; various components of context in the Consolidated Framework for Implementation Research; etc.

What I like about this application is that it is taking advantage of something VA is going to do anyway, and is trying to study it to learn something both about the target intervention and the process of implementation in general. It is being led by experienced investigators who are solidly locked in to VA research and operations.

In their prior application the investigators made much of their belief that the process of de-emphasis will differ from the process of emphasis. They have de-emphasized this potential difference in this revised application, which now treats de-implementation more as another kind of implementation problem rather than as a unique and not-yet-ever-before-seen kind of problem.

Another problem in their original application was their sample size calculations. This appears to be fixed in this application. I leave this to better men than I.

A subtle point I made in their prior application was about change being „easier‟ to implement when it involved “de-implementing” something that had been recently implemented, rather than imply achieving (or not) low A1c targets. They‟ve addressed this by dealing with the change score to identify high and low outliers. That‟s OK. But I offer up here another way to approach their problem, which could be done in place of or in parallel with their current planned analysis. I suggest they consider using Statistical Process Control and Run Charts to identify both special cause variation (which could be due to the adoption of Choosing Wisely) and to identify particular high and low outliers for the second part of their project. So, to do this, the investigators will need to establish the current process prior to April 1, 2014. They could do this by breaking down into quarters (or months, but quarters is probably more stable) the occurrence of their outcomes (proportion of patients with Hgb A1c less-than-whatever in patients with dementia, advanced age or creatinine greater than 2.0) by facility for the prior 3 years.

This then gives them 12 datapoints, enough to have a sense of the process at each facility, how stable it is, whether it is moving up or down already, etc. Then let this run out another 4 – 6 quarters after April 1st (or whenever Choosing Wisely gets off the ground). Then, use established criteria for determining whether there is special cause variation going on at any site (i.e., a point above or below the upper or lower control limits, or a cluster of consecutive points all below (or above) the mean).

They could even have this part of the analysis done by people who are blinded to adoption of Choosing Wisely. In other words, one set of analysts looks at all 130 facilities or whatever and then tells the PIs which facilities meet the criteria for special cause variation. The PIs then match this to the list of which facilities adopted the Choosing Wisely diabetes campaign. You‟d expect that more facilities with special cause favorable variation are Choosing Wisely adopters more often than not, and this could be tested statistically. The investigators could also use the Run Charts to pick out the high and low outliers, I would expect the Run Charts to be more informative about how the process is actually working than the aggregate pre/post measure they currently propose. This may then increase their

ability to actually identify important contexts and processes, since they‟d have a (probably) better selection of differences in performance between facilities.

In the prior application I indicated that the distribution of contexts may not be as favorable as they are hoping it will be, and they may need a plan B. In this version, the investigators say this isn‟t their worry, they are confident there will be differences. So be it. But I will reiterate that I would be worried about there being enough sample in the various cross tabs of their predictor variables, like participation in the Choosing Wisely campaign, some categorical groupings of Commitment to Quality and Safety Culture and Teaching intensity, etc. I share their confidence that they are likely able to do each predictor variables as a univariate analysis, but it is the independence of these in a multivariable analysis that implementation science experts will be interested in and about which I am worried.

One last point to bring up is that the prior application was full of grammar mistakes and typos. This reviewer greatly appreciates the extra care that went into this application, which has cleaned this up.

# Overall Impression.

See above

# Key Strengths.

- 1. A time sensitive evaluation of a major VA rollout for an important patient population
  2. Looks at overuse of care
  3. Experienced investigators locked in to VA research and operations

# Key Weaknesses.

1. No key weaknesses

# Critique 2

1. **Significance and alignment of proposed specific aims with QUERI and VHA goals.**

The specific aims are well aligned with QUERI and VHA goals as follows:

**Aim 1.** To assess the *overall* impact … of the Choosing Wisely Initiative.

The Choosing Wisely Initiative is an important VA initiative, with Drs. Schectman and Pogach as co- chairs. The 2011 DM QUERI strategic plan discusses the problem of overtreatment in considerable detail and notes the goal of encouraging more moderate glycemic control in subgroups at particular risk of hypoglycemia and/or unlikely to benefit from tighter control.

**Aim 2.** To assess the impact of commitment to quality, teaching intensity, and safety culture on patients‟ likelihood of overtreatment. This is an assessment of the importance of facility characteristics on implementation – this understanding of what impacts the success of implementation efforts is a goal of the Diabetes QUERI in particular and QUERI in general.

**Aim 3.** To identify configurations of the implementation strategy (Choosing Wisely toolkit), provider characteristics and organizational level factors that are associated with … reduction of overtreatment … This is again consistent with QUERI goals of better understanding what impacts implementation success.

All three of these aims are significant in that overtreatment of people at high risk for hypoglycemia is associated with increased healthcare costs and worse outcomes. It seems very likely that reduction of

this overtreatment would mitigate these consequences. However, it is not clear that reducing this by the approaches taken in the CWI would cause an improvement in costs and outcomes. Thus, this study will give us good data on how best to address the problem; at the least it will give us good data on whether the approach taken is useful. If it turns out that, in fact, all VHA facilities, not just those targeting overtreatment of DM as part of the CWI, have reductions in both overtreatment and its consequences, it would suggest that this is not a good way to change practice. Or at least that the centrally advised/directed facility specific implementation efforts don‟t add much to simply pointing out that this is a problem and giving people feedback.

The specific aims are also significant for implementation science. If the constructs examined in Aim 2 are associated with implementation success, this will suggest that future implementation efforts should attend to them, or at least be aware that measures of teaching intensity, safety culture and commitment to quality can identify sites where the task of implementation will be more or less challenging. The use of the Consolidated Framework for Implementation Research (CFIR) also means that the DM QUERI will be able to add this to a growing list of projects where CFIR constructs are measured and examined in relationship to implementation success.

# Impact on Veteran Care and Implementation Science:

As the researchers note, the ability to make direct inference about the particular constellation of toolkit components that are effective will be limited by the fact that details about the implementation approach are only going to be available from a small number of sites that are identified as high performers or are selected as a control. This analysis will thus lack data needed to find a dose response effect, it will be underpowered, and it will be susceptible to the dangers of multiple hypothesis testing, since there are multiple toolkit components that will be examined.

Similarly, the findings regarding teaching intensity, safety culture and commitment to quality will still be limited by the number of facilities (30) where there is survey data available. Moreover, the various data sources for safety culture and commitment to quality will be quite imperfect measures of the constructs of interest, so this introduces significant noise to an analysis of the relationship between the underlying constructs and the outcome. They are using quintiles as the independent measure, so it is an ordered categorical variable – my guess is that they will treat it as a linear variable, but they may be doing something else to allow for the idea the relationship might not be linear.

The impact on veteran care is potentially large. If they can demonstrate that participation in the CWI, however that is defined, is associated with important improvements in patient outcomes, it would be a big win. Unfortunately, they are aiming to show that participation will decrease the number of people with a lower A1c than most people think is necessary, which is less exciting. Nonetheless, the face validity of their assumption that such a reduction will reduce adverse consequences is quite high and would likely support a lot of pressure for the facilities that have yet to jump on board, to do so. This is especially true if this (proportion of high risk people who are below the lowest reasonable goal) becomes a performance measure.

# Approach:

This is the most challenging part of the proposal. Since they are taking advantage of a natural experiment, they have to work with the allocation of interventions that they get. I will focus on a couple of the issues that will make it hard to do their project and/or will weaken inferences they can draw.

First, it is not clear, or at least they did not make it clear to me, that they have a very clear idea of what will constitute participation in the DM risk reduction part of the CWI. This is Aim 1, and it is the advertised important information for the CWI sponsors, so it is disappointing they don‟t have a clearer

idea how they are going to classify a VAMC as participating. I think that they could at least have provided a sense of what it means when facilities “choose one of two overuse/low value care issues to address.” They do have close ties to the Initiative. Will the VAMC Director send a letter signing up so they get access to the toolkit? Will the toolkit be put out on the web and anyone who wants can take the parts they want? Are there any resources that come with participating? Are there any responsibilities that come with participating? On the research side, they should have commented on how they will handle a facility that trials this in a CBOC, but not the main facility, or vice versa. If a veteran gets care in two places, to which is he or she allocated? Is there some minimum effort that will be needed even if the Director did send the letter saying they want to focus on this? Will VISN 12 be excluded? What about VISN‟s 11, or 10, where the DM QUERI or the PI are located? All these VISN‟s have reasons to be atypical.

Second, the statistical approach is not well specified. The analysis is described as looking at changes over time based on individual patients, nested within facilities, but many will only show up in one or the other time frame. It is not clear which time periods are used – it looks like about a year. But if the initiative starts in April, and there is some time frame to ramp up, it seems unlikely that a person seen in May will be affected. Issues around visit and lab frequency will be important and may well vary by VISN. Given the 15 page limit, I recognize that there are constraints on methodological detail, but one could imagine less detail and/or less repetition of the new consensus that overtreatment is common.

While it is typical that some decisions will be made in the analysis process, based on review of the data structure, it would be valuable to specify a base case approach.

Third, the process by which they will identify the sites for survey administration is quite challenging. The time frame indicates they will begin the analysis of the impact of the CWI roll out starting 12 months after the roll out day, at the same time as they make the final identification of those facilities that are participating in the hypoglycemic safety part of CWI. It seems likely that different VISN‟s will begin the actual intervention on variable dates which will make it harder to choose when the site has had a year of implementation. The high and low outliers in terms of improvement, as well as a sample of sites that seem stably high or stably low in rates of overtreatment, will be identified using criteria that are a combination of quantitative data and an effort to get a range of facility characteristics. Again, it would help to have some sort of base case assumption, although it is reasonable to consider adaptation.

Fourth, the interpretation of survey results is also challenging. A wide range of questions will be asked in this 25 minute survey administered to a sample of 30 individuals per site. These will be reviewed to identify factors that at least 75% of respondents think are important or very important in “affecting successful implementation of the Choosing Wisely Initiative.” But lots of data about the clinicians and their beliefs is also collected; I can‟t see where it is used except that a note appears at the end saying that they will look at “correlations between provider characteristics and performance status (low vs. high).” They note that they have used similar survey methods in multiple prior studies, but don‟t provide data around the results of those prior surveys and studies. The survey and interview together will help to inform the site visits to the high outlier sites. These site visits do not have a role in the analysis phase, but will be used to “obtain detailed information on site-level manifestation of the constructs associated with implementation success.” Moreover, “… data from the visits to high performing sites will be analyzed descriptively for identifying best practices, and the findings from these analyses will then become the basis of our specific recommendations for improving dissemination of the Choosing Wisely Initiative.”

There are some missing pieces – the rationale for the choice of the independent variables in Aim 2 is not clearly presented. Most places in the analysis discuss high and low outliers, but the 30 medical center estimate includes a similar number of VAMC drawn from among the places that start high or low and stay there. How this is operationalized is even less clear than how the criteria for picking the high and low outliers will be balanced. Similarly, the people who will be surveyed is not clear – although it

will include “clinical leaders and PCPs and other clinicians.” And the people who will be interviewed is not clear – the text notes “(the clinical pharmacy specialist, director of primary care, and primary care providers)” but which PCP‟s and which clinical pharmacists is not clear. The site visits will apparently involve clinic visits, a lot of interviews and probably a focus group, since this is referenced in the appendix, though not in the text. Except being called descriptive, analysis is not specified.

# Overall Impact, Potential for Sustainability, and Dissemination:

The impact of the study is likely limited, since the process is under way and the results are unlikely to change VA practice. It is likely to be sustained. Dissemination is a particular strength. The close relationship of the study team to operations personnel, several who sit on an actively engaged steering committee, is likely to lead to rapid dissemination within VA. Moreover, both Dr. Aron and Dr. Pogach, the steering committee chair, have extensive ties to non-VA diabetes organizations that are interested in this issue.

I think that the results will be used and that the processes that appear to be helpful *to the people at that site* are likely to be sustained. I am not as sure that the study will change anything about the process of further roll out. That there will be some rollout is a decision that has been made and it is unlikely that such mechanisms as CPRS reminders, order sets and feedback to clinicians will not be used. Since it is certain that there will be some tailoring to the site, and that the people at the site will believe that there needs to be some provision for tailoring, this will also be an option. I think that when a site chooses its intervention, their experience cannot be generalized to the sites that did not want to use that intervention.

# Personnel and Management:

They have involved operational leaders on the steering committee. Dr. Aron is an experienced HSR investigator who has been involved in studying a number of operational innovations; he is currently Director of the Office of Specialty Care Transformation/VA HSR&D QUERI Cleveland-Ann Arbor-East Orange Evaluation Center. Dr. Lowery is an experienced implementation researcher who has worked with Dr. Aron and is Associate Director of the Michigan COIN and Implementation Research Coordinator for the Diabetes QUERI. Dr. Aron has also brought in quantitative expertise from the East Orange VA.

# Human Subjects Protection:

There is awareness of the risks to patient privacy of the quantitative data analysis. They note that they have taken care of this type of data before, and describe appropriate safeguards. The discussion of using VINCI seems quite cursory and makes one wonder if they have used *that* resource in particular. Likewise, the investigators are aware that the professionals who respond to surveys and interviews have some risk of harm and therefore take some steps to preserve their anonymity. While they could have described steps in more detail, the fact they recognize it as an issue provides me with some reassurance that the details will be appropriate.

# Budget:

The challenging array of data collection activities leads to a tight budget. There seems little room to cut analytic, data collection or data interpretation time. One wonders if there will be sufficient time for the study team to generate reports for the scientific literature, given that Dr. Aron will have his hands full keeping track of the multiple moving parts of the project.

# Overall Impression.

An expert team has been interested in this area for some time and is working closely with operational leaders who have decided that changing this practice is a priority. This close coordination with Primary and Specialty Care, and the shared sense of the importance of this issue, makes it likely that the results of the research will be used. Moreover, they take care to use methods that can contribute to future assessments of the relationship between implementation strategies and outcomes.

However, this close collaboration has the potential to lead to choices that give unreliable results, particularly when the methods are so fluid. While it is reasonable to be adaptable to circumstances due to this being a natural experiment, the investigators do not make a good case for why they cannot provide a structured approach that they plan to implement. The analysis plan currently seems to be something of a set of tools that they will apply and interpret in the way that makes the most sense at the time. I worry this will cause them to make choices that will cause them to come to the conclusion that they presuppose is correct.

# Key Strengths.

- 1. They are looking at a common problem with significant morbid consequences that has the attention of VA leadership.
  2. The use of mixed methods is a good idea, even though the description is quite challenging.
  3. Each of the investigators has important expertise and experience with the problem. Dr. Aron, in particular, has been working on this area for some time and is intimately familiar with the various expert bodies working on this issue.

# Key Weaknesses.

1. Research plan is excessively fluid.
2. The key independent variable for Aim 1 is not defined. The rationale for the choice of independent variables in Aim 2 is also not provided. The site visits are poorly justified. The presence or absence of CFIR constructs is characterized without consideration that how one does things is important, not simply that they are done.
3. The natural experiment nature of the study creates substantial barriers to comparisons among sites, which are likely to be at different stages in the implementation process, even if they are using similar tools.

# Critique 3

1. **Alignment of proposed specific aims with QUERI and VHA goals:**

The proposal is aligned with the broad DM QUERI aim of promoting evidence-based approaches to treatment and to reduce the complications of DM. The proposal is directly aligned with VA efforts to implement the “Choosing Wisely” Initiative – which is supported by DM QUERI and PCS.

# Evidence base is adequate for Implementation:

The evidence base that strict glycemic control is not beneficial and potentially harmful in certain subgroups with DM is sufficient to lead to changes in CPGs (even among true believer organizations

such as the ADA). The evidence comes from several large RCTs and is convincing. Although the evidence is not directly from VA populations, there is no reason it would not apply to the VA population. A potential weakness in the evidence base and study logic is that the target population (and the outcome) is those who are *potentially overtreated* – as we don‟t know and won‟t know from the data collected if patients are having hypoglycemic episodes or hypoglycemic-related adverse events (e.g. fall and hip fracture) are reduced. Nevertheless, data are presented that hypoglycemic episodes, including those that are serious in nature occur with sufficient frequency to justify the effort to reduce even the proportion who are *potentially overtreated*.

The evidence base for the Choosing Wisely campaign is less well established, but it appears to be based on current implementation theory. It remains unclear whether the campaign is tailored to special issues related to de-implementation but investigators at least acknowledge other examples such as changes in data about medication effects (e.g., antiarrhythmics during acute coronary syndrome, estrogen) or changes in guideline recommendations (e.g., dropping need to monitor LFTs for patients on statins). The exact plan for implementation, other than creation of a toolkit and designation of clinical champions (who may receive support from PBM) remains unclear. Nevertheless, it is the national VA implementation plan for this problem and there is value in understanding its effectiveness.

# Research aims and methodology are appropriate:

The overall research aims have been focused compared to the original submission and the methodology is better matched to meeting these aims. The aims appear achievable and the design more straightforward.

The research aims are stated clearly, important, and are strength of the proposal. The aims address the intended intervention effects (overtreatment rates), unintended effects (under treatment) and organizational factors (i.e., quality commitment, teaching intensity, safety culture) related to patients likelihood of overtreatment. An important and novel aspect of the proposal is the focus on understanding factors unique to “de-implementation.” There is clear support from the DM QUERI (including monetary support), the Choosing Wisely Initiative, and Primary Care Operations. Patients are not represented in the proposal – due to IRB complexity – but the investigators plan to submit a separate proposal to address patient perspectives. While the rationale for not involving patients directly is understood, this is a relative weakness as patients‟ attitudes, priorities, and knowledge may be important barriers to achieving a reduction in over-treatment.

The overall plan is linked to a sensible theoretical framework that now addresses important contextual issues. The design will capitalize on natural variation in the success of implementation.

# Research Design:

- 1. Overall approach: The overall approach to evaluate a natural experiment is appropriate – using mixed methods analytic approaches. Analyses attempt to adjust for baseline inequalities likely to be observed between groups, and the effects of time. Evaluating the entire system (131 facilities with >100 patients with DM) with deep dives at selected facilities is a strength. Overall, the approach is consistent with best practices for evaluating organizational interventions. However, the design remains subject to factors beyond the investigators control – principally the number of sites that opt to adopt the choosing wisely diabetes campaign and the selection bias that may occur through non-random assignment.
  2. Patients/sample: There appear to be ample patients (>285,000 with DM and at elevated risk) in the 131 facilities. Power calculations presented show sufficient power to detect relatively small, clinically important effects.
  3. Investigators will identify high and low performers, then do a deep dive to try and understand factors related to reduction in overtreatment. Survey and interview data will be used to collect data on domains (e.g., safety culture) that may be related to the outcome of interest. Some of these measures appear to be relatively weak and could mask true associations. Also, because interviewers will know the performance of each facility, bias could be introduced in the collection and analyses of qualitative data.
  4. Measurement:

1. Provider surveys will now be focused on the subset of high and low performing facilities. This change in approach is sensible and should allow the investigator team to concentrate on maximizing the survey response.
2. The primary quantitative outcome measure –potential over-intensive glycemic control (assessed quarterly) – seems conceptually sound although this measure would be stronger if we had validation data showing the linkage to clinically meaningful outcomes. It would be more informative if outcomes related to downstream consequences of hypoglycemia were measured such as ED visits for hypoglycemia.

Timeline: The timeline is well summarize in a GANTT chart and appears sensible. Dissemination plans are adequate and standard.

# Human Subjects Protection:

Appropriate measures are described to protect confidentiality. Risk of harm is minimal. No concerns.

# Project Organization and Management:

The project leaders are stellar and have the content expertise (both in DM and implementation science) and research track record to successfully lead the project. The project management plan is sensible.

Other team members have the skills and experience to be successful in their respective roles.

# Budget:

Although relatively expensive since this project is restricted to evaluation of a VHA

central office initiative, the budget seems appropriate and adequately justified. It is likely that patient perspectives could be sought and incorporated into the evaluation for little additional expense.

# Impact and Sustainability:

It is clear that overly tight glycemic control is problematic for individuals overall and for Veterans. If the Choosing Wisely Campaigning is effective, it will decrease the rates of overtreatment and should decrease the rates of important adverse medication effects. There is the potential for downstream cost- savings (less medication, fewer complications of hypoglycemia) and improved veteran health status.

The proposal has the potential to: a) better understand the effectiveness of the national choosing wisely initiative, b) advance implementation science through a focus on differential measures required for de- implementation.

# Overall Impression:

Overall, this study proposes to evaluate the effects of a national initiative to implement revised guidelines for glycemic control in patients with diabetes. The initiative and study is particularly interesting because the change in guideline is for more permissive control in a large subset of patients. Strengths of the proposal in include the consistency with QUERI and National VA priorities, sound overall research design, and many best practice approaches to evaluation. Weaknesses include: non- experimental design for determining the effectiveness of the intervention, lack of involvement of patient stakeholder groups, and a target population and outcome of “potentially over-treated” without clear knowledge of rates of hypoglycemic episodes or its related harms. Overall, this is a much improved proposal that has the potential to contribute to understanding of large VA central office initiatives.

**Key Strengths and Weaknesses**: Strengths:

1. The proposal is aligned with the broad DM QUERI aim of promoting evidence-based approaches to treatment and to reduce the complications of DM. The study aims are important and include the novel concept of “de-implementation.”
2. Overall, the evaluation approach is consistent with best practices for evaluating organizational interventions.
3. The research has good support from the National PC office and QUERI.

Weaknesses:

1. An important potential weakness in the evidence base and study logic is that the target population (and the outcome) is those who are *potentially overtreated* – as we don‟t know and won‟t know from the data collected if patients are having hypoglycemic episodes.
2. While the rationale for not involving patients directly is understood, this is a relative weakness as patients‟ attitudes, priorities, and knowledge may be important barriers to achieving a reduction in over-treatment.

**MEETING ROSTER**

**HQ1 QUERI SDP REVIEW**

**Quality Enhancement Research Initiative Parent Office of Research & Development**

**QUERI SDP Review HQ1 R**

**February 20, 2014**

**CHAIRPERSON** SINNOTT, PATRICIA L., PHD HEALTH ECONOMIST

VA HEALTH ECONOMICS RESOURCES CENTER (HERC) VA PALO ALTO HEALTH CARE SYSTEM

MENLO PARK, CA 94025

**MEMBERS**

GOLDSTEIN, MICHAEL G, MD ASSOCIATE DIRECTOR

BAYER INSTITUTE FOR HEALTH CARE COMMUNICATION WEST HAVEN, CT

JACKSON, GEORGE LEE ASSISTANT PROFESSOR

HEALTH RESEARCH SCIENTIST, HSR&D DEPARTMENT OF VETERANS AFFAIRS (152) 508 FULTON STREET

DURHAM, NC 27705

KIRCHNER, JOANN E, MD

2200 FORT ROOTS DRIVE BLD 58 NORTH LITTLE ROCK, AR 72114

KREIN, SARAH , RN, PHD

CTR FOR CLINICAL MGMT RESEARCH, VA ANN ARBOR RESEARCH ASSOCIATE PROFESSOR, INTERNAL MEDICINE

DIVISION OF GENERAL MEDICINE UNIVERSITY OF MICHIGAN

ANN ARBOR, MI 48105

POST, EDWARD P, MD, PHD

VA HSR&D (152) 2215 FULLER RD. ANN ARBOR, MI 48105

SCHNURR, PAULA P., PHD HSR&D SMRB CHAIR

DEPUTY EXEC DIR VA NAT CTR FOR PTSD WHITE RIVER JUNCTION VA MEDICAL CENTER RESEARCH PROFESSOR OF PSYCHIATRY DARTMOUTH MEDICAL SCHOOL

WHITE RIVER JUNCTION, VT 05009

SHEKELLE, PAUL G., MD, PHD DIRECTOR

SOUTHERN CALIFORNIA EVIDENCE-BASED PRACTICE CENTER

RAND

SANTA MONICA, CA 90401

SMITH, BRIDGET M., PHD SOCIAL SCIENCE ANAYLST HSR&D, CMCCC

EDWARD HINES JR VA HOSPITAL RESEARCH ASSISTANT PROFESSOR NORTHWESTERN UNIVERSITY CHICAGO, IL 60141

SOX-HARRIS, ALEX , PHD

VA PALO ALTO HEALTH CARE SYSTEM PALO ALTO, CA 94304

TURVEY, CAROLYN L, PHD ASSOCIATE PROFESSOR DEPARTMENT OF PSYCHIATRY UNIVERSITY OF IOWA

IOWA CITY, IA 52242

WHITTLE, JEFFREY C., MD PROFESSOR

HEALTH SERVICES RESEARCH & DEVELOPMENT ZABLOCKI VA MEDICAL CENTER

DIRECTOR, SOUTHEAST WISCONSIN ALLIANCE FOR TRANSLATING RESEARCH INTO PRACTICE MILWAUKEE, WI 53295

WILLIAMS, JOHN W, MD ASSOCIATE PROFESSOR

DEPARTMENT OF MEDICINE & PSYCHIATRY HEALTH SERVICES RESEARCH & DEVELOPMENT DUKE VA MEDICAL CENTER

DUKE UNIVERSITY DURHAM, NC 27705

WILLIAMS, LINDA S, MD ASSOCIATE PROFESSOR

DEPARTMENT OF NEUROLOGY, INDIANA UNIVERSITY RESEARCH COORDINATOR, VA STROKE QUERI INVESTIGATOR, VA HSR&D CENTER FOR IMPLEMENTING EVIDENCE-BASED PRACTICE

INDIANAPOLIS, IN 46202

**SCIENTIFIC REVIEW ADMINISTRATOR**

KILBOURNE, AMY M, PHD ASSOCIATE PROFESSOR DEPARTMENT OF PSYCHIATRY

UNIVERSITY OF MICHIGAN, SCHOOL OF MEDICINE RESEARCH HEALTH SCIENCE SPECIALIST SMITREC, VA ANN ARBOR HEALTHCARE SYSTEM ANN ARBOR, MI 48105

MCIVOR, LINDA

SCIENTIFIC REVIEW ADMINISTRATOR HSR&D QUERI

DEPT OF VA, VETERANS HEALTH ADMINISTRATION WASHINGTON, DC 20420

Consultants are required to absent themselves from the room during the review of any application if their presence would constitute or appear to constitute a conflict of interest.
