# Supplementary material for: De-implementation of inappropriately tight control (of hypoglycemia) for health: protocol with an example of a research grant application
Source: Implement Sci. 2014 May 19;9:58. doi: 10.1186/1748-5908-9-58 (PMC4046046; doi:10.1186/1748-5908-9-58)
Supplement: Additional file 6 — Response to the critiques. [file 1748-5908-9-58-S6.pdf]

1. Introduction to the Proposal. We thank the reviewers for their comments and are gratified that *1. The proposed study is well aligned with the DM QUERI aim of promoting evidence-based approaches to treatments; and 2. The approach of "de-implementation" is novel and important to investigate.* After considerable reflection upon the very helpful comments of the reviewers, we have narrowed our questions and propose a study that is much simpler in design, yet still provides much important new information. Because of the extensive rewrite and the page limit reduction, we have not highlighted the specific changes in the body of the text.

3. Obtaining the appropriate sample size for this study may be problematic; and 4. The power calculations suggest that the study would be under-powered to even address Aim 1. This should be addressed.

First, we have re-focused the project as an assessment of a national initiative for hypoglycemia risk reduction that promotes a multi-pronged toolkit to facilitate patient evaluation and action rather than an assessment of the specific elements of one portion of that toolkit, which we previously referred to as the Multi Hypoglycemia Risk Reduction (MHRR) Intervention. Second, our primary analysis will use patient-level data rather than facility-level data. Even with patients clustered within facilities within VISNs, the large increase in sample size results in substantial increase in power to address the primary research hypotheses (see section 2.4.4.).

5. Research Question 2.1 and 2.2 should be dropped from this study.

We agree with the suggestion to drop Research Question 2.2 (How does de-implementation differ from implementation from a clinician perspective?), but we respectfully disagree with the suggestion to drop research question 2.1 (Which configurations of the MHRR intervention components and which factors are associated with greater reduction in overtreatment rates?) from the study, now designated 3.1 and 3.2. These questions are the *sine qua non* of implementation research: what works where and when? If we do not find an effect of the intervention, it is not necessarily because the intervention didn't work or isn't a good one, but very possibly that it didn't work under certain circumstances—i.e., that some sites had greater difficulty in surmounting organizational barriers to implementation than others. Like the proposed study, many implementation studies do not involve a sufficiently large number of sites for conducting traditional hypothesis testing at the organizational level. However, this should not preclude us from seeking to identify potential barriers and facilitators to implementation. The approach proposed in this study will not only provide useful information to our partners in the form of feedback from key stakeholders at the sites, but will also allow us to collect the data in such a way that we can contribute to a repository of data that we are collecting across multiple studies, which eventually should provide us with a sufficient N to do some hypothesis testing. As noted in the proposal, we have already used this approach in multiple studies and produced extremely useful findings for our partners. We have added information to the section describing our use of qualitative comparative analysis to address Reviewer #2's questions regarding what variables will be considered as candidates for inclusion in the model. We have also provided additional information on the site visits.

6. Drop Hypothesis 1.3 or justify that these medications will be available in VA.

We have dropped hypothesis 1.3 from the study. If we find that high performers are getting access to and prescribing alternative medications (which have been promoted by professional societies -- Sequist ER, et al. "Hypoglycemia and Diabetes: a Report of a Workgroup of the American Diabetes Association and The Endocrine Society," Diabetes Care, May 2013. 36(5):1384-95), we can revisit this issue.

7. The quantitative analysis and qualitative data collection methods are unclear (see critiques).

The methods sections have been substantially rewritten and clarified. Most importantly, by focusing on the initiative rather than the MHRR, we can utilize data that are routinely collected for operations for the quantitative analyses; these data are readily available (see 2.4.5.2). Assessment of how facilities were successful will be accomplished by assessing high and low performing sites and obtaining data from a combination of key informant interviews, surveys, and site visits. We will not be relying on clinical pharmacy specialists or clinic managers to be the sole source of data and we will not be collecting data on the MHRR at multiple time points.

8. It is unclear if there is an alternate plan to address variation in safety culture and ORCA, or lack thereof.

First, prior studies both in VA and private sector facilities have shown substantial variation in safety culture (e.g., see Hartmann CW et al. Relationship of Hospital Organizational Culture to Patient Safety Climate in the Veterans Health Administration. Med Care Res Rev 2009 66:320). Thus, we are confident that there will be variation again across facilities. We recognize that the VA Survey implicitly focuses on inpatients rather than outpatients, but several domains are relevant. We have dropped the ORCA survey from the analyses in Aim 2. Rather, we will be focusing on high and low performing sites and using items from Veterans Assessment and Improvement Laboratory (VAIL) VISN 22 Survey of Primary Care Team Experiences to assess safety culture.

We will also obtain more in depth assessment of aspects of safety culture as they relate to the Choosing Wisely Initiative specifically in the semi-structured interviews and site visits.

9. Study team FTE, in particular the PI at 10%, does not seem sufficient.

The budget includes substantially revised percent efforts in order to ensure commitment of appropriate expertise.

Additional responses to individual reviews

Critique 1. *Another methods issue they are going to want to consider is the recent change over time in their outcome measure at the various sites.*

We completely agree and have changed our approach accordingly. Specifically, we define low and high performance based on *change* (reduction in overtreatment rate) rather than absolute rate.

Lastly, I could not understand at all the text on pages 29-33 in terms of research question 2.1, "Which configurations of the MHRR intervention components and which factors are associated with greater reduction..."; "It is very hard for me to believe that they are either going to get the necessary spread of the intervention components across their 30 facilities (that is, the naturally occurring variation is going to provide them with an adequate distribution to try and estimate effects…": " consider cutting from the plan are propensity score analysis and instrumental variables approach".

We agree that a purely quantitative approach to assess this question would be difficult if not impossible. We have eliminated those analyses and focus on the qualitative comparative qnalysis as a way to address the question.

Critique 2. *"There is relatively little discussion of whether the MHRRI components are the ones that one should use…In their defense, they can argue that the strength of evidence is not all that important, since the VA has decided to invest considerable resources in the deimplementation project. Thus, an effort to understand what about the MHRRI did and did not work is warranted simply on that basis…"; and "I would argue that the MHRRI starts on 1/30/14 regardless of when individual facilities put in place any of the components that are planned. This would also simplify the analysis – was the program able to change slopes in VA overall. The idea that some facilities will be more or less successful is all part of the answer."*

These two comments were critically important in terms of clarifying the way we conceptualized the project. Our primary goal is to assess a national initiative which can be done quantitatively with precisely defined variables. We have dropped the purely quantitative approach to assessing aspects of the intervention (really the toolkit which is evolving) and instead use a qualitative approach.

They are clearly at the mercy of the VA's decision on how to implement MHRRI. They will get a non-randomly selected subset of VAMC and CBOC's that will choose to focus on this Choosing Wisely topic, while others focus on other topics – we are not told what they will be…

This is definitely the case in a natural experiment; however, the only other initiative topic (as of 12/31/13) on which facilities/VISNs could focus has now been defined --reduction in MRI use in evaluation of low back pain.

They are further handicapped by needing to cobble together a description of what each facility is doing based on reports from surveys of clinical pharmacy specialists and clinical managers. It appears that the surveys are going to continue to be repeated at some interval since they will define a facility as participating when it achieves 3 intervention components based on survey responses.

We have eliminated the need for repeated surveys by focusing only on comparing high and low performers.

This project seems to be too broadly focused. Again, I would feel better if there were some ideas going into the project regarding specific questions that should be addressed.

We have narrowed the focus and identified a number of specific questions.

Critique 3. *The investigators state (and I agree) that the intervention meets the definition for a complex intervention, but the intervention while multicomponent is not described in detail and appears to be a hodgepodge of elements. Some of the educational materials appear weak or overly complex and are constrained by VA's algorithmic approach to CPG development. Finally, a similar intervention piloted in the investigators VISN only produced modest results.*

We have clarified that our primary goal is to assess a national initiative. The multiple components of what is now referred to as the toolkit should not be viewed either as a "hodgepodge" or a single intervention, but rather a series of options that will address key elements of provider and patient shared decision-making based upon already developed VA-DoD tools, with different elements at different stages of maturity. Moreover, there are

groups in VA (Patient Care Services and Employee Education System) working on other materials so that this toolkit is evolving, in collaboration with Health and Human Services. Our new approach to analysis now uses patient level data which gives us sufficient power to identify even small differences.

While the rationale for not involving patients directly is understood, this is a relative weakness as patients' attitudes, priorities and knowledge may be important barriers to achieving a reduction in over-treatment.

We agree that patients are a critical part of any strategy to reduce overuse. The theoretical cornerstone of the campaign will be shared decision-making with patients around individualized, targeted glycemic goals. This will be an emphasis not only of VA's initiative, but also Health and Human Services' National Action Plan. However, evaluation of patient factors would be complicated by the fact that many patients who are at higher risk for hypoglycemic events may comprise a vulnerable population by virtue of age, cognitive impairment, and alcohol/substance use and/or serious mental conditions, which might compromise decision-making, and necessitate the inclusion of family members. The complexity of this issue, from our perspective, is best addressed first by pilot studies and we plan to submit an RRP to that effect. Provider perceptions of patients' attitudes will be assessed, but direct survey or interview of patients is beyond the scope of this proposal.

The overall plan is linked to a sensible theoretical framework, although surprisingly it omits contextual issues such as degree of local change (e.g., provider turnover), provider morale, and competing priorities – issues known to be important to implementation in primary care settings.

We plan to obtain data related to these and other factors in our qualitative assessment of high and low performers.

An important potential weakness in the evidence base and study logic is that the target population (and the outcome) is those who are potentially overtreated – as we don't know and won't know from the data collected if patients are having hypoglycemic episodes.

The initiative addresses risk. The same approach is used in treatment of LDL-cholesterol and blood pressure. We don't know if individual patients are suffering from ill effects at the time treatment is started. Rather, they are at risk for future adverse events. If tight glycemic control had clear benefit in our target population, then the identification of those having ill effects would be far more important. However, in the current situation, people are put at risk for little or no benefit.

Critique 4. *The aims and methods are generally appropriate but I do have some specific concerns. 1) some of the variables and outcomes are not well defined...*

We have defined the measures and use published methods (relying on last lab value in the relevant time period. (Tseng et al. JAMA Internal Medicine online 12/9/13). Measurement of adverse events, specifically hypoglycemia, is problematic for a variety of reasons including undercoding. We make no claims to be able to accurately determine the effect of the initiative on actual hypoglycemic events.

4) I am concerned that the surveys and role of the pharmacists to provide the lists of overtreated patients at each facility may prove a) to be too burdensome and b) may be fraught with missing data.

We agree and have eliminated this part of the project.

5) The proposal notes that there will be a manual review process to assess patients for receipt of non-VA hypoglycemic medications...

We use only data from VA's Computer Data Warehouse. (*Tseng et al. JAMA Internal Medicine online 12/9/13).*

6) The top/bottom 5% of the facilities will be selected for further study to identify the barriers and facilitators to implementation, but from the proposal, it appears that facilities will be assessed in the context of the performance of their VISN… I am concerned that if a VISN is high or low performing, facilities selected may not be true outliers when compared with the spectrum of VHA facilities.

We have clarified our methods (now published) for identifying low and high performers and positive deviants.

I am concerned that the dissemination plan lacks sufficient detail…neither front line primary care providers nor endocrinologists are listed in the table of end users.

We have broadened stakeholder representation and provided more specifics. We note the strong support in new letters from Dr. Schectman (Primary Care) and Dr Pogach (Specialty Care).
